# Upregulation of CD36, a Fatty Acid Translocase, Promotes Colorectal Cancer Metastasis by Increasing MMP28 and Decreasing E-Cadherin Expression

**DOI:** 10.3390/cancers14010252

**Published:** 2022-01-05

**Authors:** James Drury, Piotr G. Rychahou, Courtney O. Kelson, Mariah E. Geisen, Yuanyuan Wu, Daheng He, Chi Wang, Eun Y. Lee, B. Mark Evers, Yekaterina Y. Zaytseva

**Affiliations:** 1 Department of Toxicology and Cancer Biology, University of Kentucky, Lexington, KY 40536, USA; james.drury12@uky.edu (J.D.); courtney.kelson@uky.edu (C.O.K.); mge253@uky.edu (M.E.G.); 2 Department of Surgery and Markey Cancer Center, University of Kentucky, Lexington, KY 40536, USA; piotr.rychahou@uky.edu (P.G.R.); mark.evers@uky.edu (B.M.E.); 3 Biostatistics and Bioinformatics Shared Resource Facility, Markey Cancer Center, University of Kentucky, Lexington, KY 40536, USA; ywu244@uky.edu (Y.W.); daheng.he@uky.edu (D.H.); chi.wang@uky.edu (C.W.); 4 Department of Pathology and Laboratory Medicine, University of Kentucky, Lexington, KY 40536, USA; eylee@uky.edu

**Keywords:** colorectal cancer, metastasis, fatty-acid metabolism, CD36, MMP28, E-cadherin

## Abstract

**Simple Summary:**

Colorectal cancer is the second leading cause of cancer-related death in the world. Upregulation of fatty acid metabolism is a hallmark of cancer and recent studies demonstrate that blocking fatty acid uptake is a promising therapeutic strategy. We have previously shown that CD36, a transporter of fatty acid, promotes colorectal cancer tumor growth. We have also demonstrated that high expression of CD36 is associated with cancer cells that are prone to metastasis. Here, in studying the role of CD36 in colorectal cancer, we found that CD36 promotes colorectal cancer invasion in vitro and metastasis in vivo and that overexpression of CD36 upregulates expression of the matrix metalloproteinase MMP28. Our data demonstrates that MMP28 cleaves and decreases expression of E-cadherin, a marker for epithelial-to-mesenchymal transition in colorectal cancers. This newly defined CD36–MMP28–E-cadherin axis provides new therapeutic targets for the treatment of colorectal cancer.

**Abstract:**

Altered fatty acid metabolism continues to be an attractive target for therapeutic intervention in cancer. We previously found that colorectal cancer (CRC) cells with a higher metastatic potential express a higher level of fatty acid translocase (CD36). However, the role of CD36 in CRC metastasis has not been studied. Here, we demonstrate that high expression of CD36 promotes invasion of CRC cells. Consistently, CD36 promoted lung metastasis in the tail vein model and GI metastasis in the cecum injection model. RNA-Seq analysis of CRC cells with altered expression of CD36 revealed an association between high expression of CD36 and upregulation of MMP28, a novel member of the metallopeptidase family of proteins. Using shRNA-mediated knockdown and overexpression of CD36, we confirmed that CD36 regulates MMP28 expression in CRC cells. siRNA-mediated knockdown of MMP28 decreases invasion of CRC cells, suggesting that MMP28 regulates the metastatic properties of cells downstream of CD36. Importantly, high expression of MMP28 leads to a significant decrease in active E-cadherin and an increase in the products of E-cadherin cleavage, CTF1 and CTF2. In summary, upregulation of CD36 expression promotes the metastatic properties of CRC via upregulation of MMP28 and an increase in E-cadherin cleavage, suggesting that targeting the CD36–MMP28 axis may be an effective therapeutic strategy for CRC metastasis.

## 1. Introduction

Colorectal cancer (CRC) is the leading cause of non-smoking-related cancer deaths and the second leading cause of all cancer-related deaths in the United States, and indeed the world [1,2]. Patients diagnosed with late-stage CRC (Stage III–IV), which exhibit local invasion and distant metastatic disease, have nearly a 10-fold decrease in their 5-year survival rate when compared to earlier stage, localized disease [3,4]. Despite advances in the treatment of primary CRC, effective therapeutic strategies for late-stage CRC are lacking. 

Altered fatty acid metabolism is recognized as a hallmark of cancer and multiple studies suggest targeting this pathway as a potential therapeutic strategy for cancer including CRC [5,6,7]. Fatty acid translocase (CD36) plays a significant role in dietary fatty acid regulation as an exogenous fatty acid transporter [8,9]. CD36 can be membrane bound, where it can bind low-density lipoproteins and extracellular long-chain and ultra-long-chain free fatty acids [8,10]. CD36 has been implicated in the promotion of primary tumor proliferation and disease progression in multiple malignancies, including ovarian, glioblastoma, and breast cancers [11,12,13,14]. Recently, our laboratory has shown the important role of CD36 in enhancing cellular proliferation and progression of primary CRC and identified upregulation of CD36 as a potential mechanism of resistance to FASN-targeted therapy [15]. 

While compelling evidence exists to support CD36’s role in the promotion of primary tumor growth and progression in several cancers, some evidence also suggests that CD36 may also play a critical role in cancer metastasis. CD36 was shown to promote oral carcinoma migration and invasion in vitro and metastasis in vivo [16]. In fact, enhanced presence of tumor cells expressing high levels of CD36 and increased metastatic potential is associated with a poor prognosis and clinical outcome in both glioblastoma and oral carcinoma [13,16]. CD36 also enhances migration and invasion in gastric cancer cells in vitro as well as local invasion and metastasis in ovarian cancer xenografts [11,12,17]. Furthermore, CD36 has been shown to promote cell growth and metastasis as well as epithelial-to-mesenchymal transition (EMT) in cervical cancer [18,19]. However, the potential role of CD36 in CRC metastasis has not been previously studied.

EMT is the fundamental process through which an epithelial cell acquires a more mesenchymal phenotype [20,21]. Aside from being utilized during normal tissue development, wound healing, and tissue repair, EMT has also been identified as one of the crucial steps involved in cancer metastasis [22,23,24]. Various molecular pathways and markers are implicated in the regulation of EMT in CRC, including Snail/Slug, Wnt/β-catenin and, particularly, the downregulation of E-cadherin, a critical component of cell-cell adhesion junctions [25]. Loss of E-cadherin is a key characteristic of EMT initiation in various cancers, including CRC [26,27]. E-cadherin expression has previously been shown to be a good prognostic marker for patients with CRC [28]. Furthermore, loss of E-cadherin increases CRC cell invasion and is correlated with poor survival rates [29,30]. Lower expression of E-cadherin in CRC patient tumors is associated with tumor differentiation, invasion depth, tumor stage and lymph node metastasis [31]. Although E-cadherin has several known transcriptional regulators, including Snail and Slug, it is suggested that E-cadherin is strictly regulated post-transcriptionally in CRC, either through stabilization or cleavage [32,33].

The matrix metallopeptidase (MMP) family of proteins plays an important role in the degradation of several extracellular matrix (ECM) components [34,35]. MMP28, one of the newest members of this family of proteins to be identified, is associated with EMT in lung carcinoma [36]. Furthermore, MMP28 is associated with a poor prognosis and lower survival rates in gastric cancer and increased expression of MMP28 is associated with an increase in invasion and colony formation of gastric cancer cells in vitro and in vivo [37,38]. Lastly, MMP28-induced EMT in lung carcinoma is associated with a significant loss of E-cadherin expression [36,39].

Since previous studies have implicated CD36’s involvement in the metastatic process and regulation of EMT in cancer, the goal of this study was to evaluate the contribution of CD36 to CRC metastasis. We found that upregulation of CD36 promotes invasion and colony formation of CRC cells in vitro. Consistently, high expression of CD36 is associated with a significant increase in metastasis in vivo. To address the potential mechanisms of how CD36 regulates metastasis, we found that overexpression of CD36 is associated with MMP28 expression in established CRC cell lines and isogenic patient-derived xenografts. Our results also show that knockdown of MMP28 reduces CRC cell invasion in vitro. Lastly, we found that CD36 and MMP28 expression are inversely associated with E-cadherin expression in CRC cells. We show that a high level of MMP28 expression is associated with an increase in the cleavage of E-cadherin and an increase in the cleavage products C-terminus fragment 1 and 2 (CTF1 and CTF2). 

Together these findings implicate that CD36 promotes metastasis via upregulation of MMP28 and E-cadherin cleavage and, thus, may be a viable therapeutic target for the treatment of metastatic CRC.

## 2. Materials and Methods

Established Colon Cancer and Isogenic Cell Lines. Established cell lines, HCT116, HT29 and HT29LuM3, were maintained in McCoy’s 5A medium supplemented with 10% FBS (Sigma-Aldrich, St. Louis, MO, USA) and 1% penicillin–streptomycin. HT29 LuM0 and LuM3 cell lines were established as previously described [40]. All cell lines were authenticated using STR DNA profiling (Genetica, Cincinnati, OH, USA).

Stable CD36 knockdown HCT116 and HT29LuM3 cell lines were established using CD36 shRNA from Sigma-Aldrich, St. Louis, MO, USA (TRCN0000057000 [shRNA#1]; TRCN0000419016 [shRNA#2]). pLKO.1-puro non-mammalian shRNA was used as a non-targeted control (NTC). Cells were selected with 10 mg/mL puromycin. Knockdown was confirmed via quantitative real-time polymerase chain reaction (qRT-PCR) after cell selection and prior to performing in vitro and in vivo experiments. Overexpression cell lines were established by transfecting HCT116 and HT29 LuM0 cells with either pLenti-C-Myc-DDK-P2A-Puro-CD36 overexpression (OriGene #RC221976L1V) or pLenti-C-Myc-DDK-P2A-Puro-Empty-Vector (OriGene #PS1000064) lentiviral transduction particles. MMP28 knockdown cell lines were generated by transfecting CRC cells with scrambled siRNA control (Invitrogen #12935200) or siMMP28 siRNA#1 and #2 (Invitrogen, HSS149127 and HSS149128) in combination with Lipofectamine RNAiMAX Transfection Reagent (Thermo Fisher #13778150). MMP28 knockdown was verified by qRT-PCR and Western blotting. Cells were transfected for 48 h prior to commencement of in vitro experiments. 

qRT-PCR. Total RNA was isolated using an RNeasy mini kit (QIAGEN). cDNA was synthesized using a high-capacity cDNA reverse transcription kit (Applied Biosystems, Bedford, MA, USA; #4368814). qRT-PCR was carried out using a TaqMan Gene Expression Master Mix (Applied Biosystems, Bedford, MA, USA; #4369016) according to manufacturer’s protocol and TaqMan probes for human CD36 (#4331182-Hs00169627_m1), MMP28 (#4331182-Hs00425232_g1), E-cadherin/CDH1 (#4331182-Hs01023895_m1) and GAPDH (#4331182-Hs02786624_g1) from Thermo Fisher, Waltham, MA, USA.

RNA-Seq and Gene Set Enrichment Analysis. mRNA samples were analyzed via RNA-Seq by BGI Genomics, Cambridge, MA. Gene set enrichment analysis was performed by the Biostatistics and Bioinformatics Shared Resource Facility at the University of Kentucky, Lexington, KY. The gene set enrichment analysis was performed using GSEA software (GSEA 4.0.3) and the KEGG pathways in the Molecular Signature Database (MSigDB) [41]. 

Trans-well Invasion Assay. HCT116 CD36 overexpression and CD36 knockdown cell lines were plated and starved for 48 h on 100 mm petri dishes with McCoy’s 5A medium without FBS supplementation. Corning 24-well BioCoat™ Matrigel^®^ Invasion Chambers (Corning #354480) were rehydrated for 2 h with McCoy’s 5A medium not supplemented with FBS at 37 °C. HCT116 cell lines were then trypsinized and counted using a Beckman Coulter Vi-Cell BLU Cell Viability Analyzer (Beckman Coulter #C19196) and plated at 50,000 cells/chamber serum free with McCoy’s 5A medium. Invasion chambers were then placed in wells containing 400 μL of McCoy’s 5A medium supplemented with 10% FBS as a chemoattractant. Cell were allowed to invade for 48 h. Chambers were then aspirated, the inside wiped with a cotton swab, and chambers stained with 0.1% crystal violet (Millipore sigma #C6158-50G) for 10 min. Invaded cells were counted in four representative fields (counting grit) per well with an inverted macroscope. Chambers were also imaged and then de-stained with 200 μL of extraction buffer from the CytoSelect Cell Invasion Assay Kit (Cell Biolabs Inc., San Diego, CA, USA; #CBA-111-T). A total of 50 μL of extract from each well was plated on a 96-well plate and absorbance was measured at 560 nm on a microplate reader for cell invasion quantification. 

Colony Formation Assay. 6-well cell culture plates were first layered with 2% soft agar using SeaPlaqueTM GTG Agarose (Lonza #50115) melted in Milli-Q water. HCT116 and HT29 LuM0 CD36 overexpressing cells were trypsinized and counted as described above. Cells were mixed with a 1.5% soft agar and 2X McCoy’s 5A medium solution and plated at a concentration of 5000 cells/well. McCoy’s 5A medium supplemented with 10% FBS was placed on top of both agarose layers and the media levels monitored. Colonies were allowed to grow for 14 days and then stained with 0.1% crystal violet (Millipore sigma #C6158-50G) for 2 h at room temperature. Wells were then washed with Milli-Q water to remove the excess crystal violet stain, imaged, and the colony diameter measured.

Confocal Microscopy. HT29-GFP-Luc, LuM0 and LuM3 cells were plated at a concentration of 10,000 cells/chamber onto u-Slide 8-well-chambered coverslips (Ibidi #80826) in 400 μL McCoy’s 5A medium supplemented with 10% FBS for 48 h. Cells were then fixed with 10% Formalin for 10 min followed by permeabilization with 1% Triton X. Cells were then blocked with 1% bovine serum albumin (BSA) for 20 min at room temperature. Cells were incubated with primary antibodies for CD36 (Santa Cruz # sc-7309) and MMP28 (Abcam #ab175937) in 1% BSA for 2 h at room temperature. Cells were then washed 3X with PBS and incubated with fluorescent secondary antibodies (Thermo Fisher#) in 1% BSA for 1 h at room temperature. The chambers were washed 3 times with PBS and incubated with Hoechst and Phalloidin stains for 20 min. Chambers were then imaged using a Nikon A1 Confocal Microscope. 

FA Uptake Assay. HT29-GFP-Luc, LuM0 and LuM3 cells were plated at 10,000 cells/well on an 8-well coverslip u-slide (Ibidi #80826) and treated with CD36 neutralizing antibody (Cayman Chemical #10009893) at a concentration of 2 μg/mL for 24 h. Mouse IgA (Abcam #37322) was used as a control. After incubation with neutralizing antibody, cells were then treated with fluorescent FA analogue BODIPY™ 558/568 C12 (Thermo Fisher # D3835) for 10 min in serum-free McCoy’s 5A medium supplemented with 10% fatty acid-free BSA. Cells were washed twice with PBS and fixed with PBS containing 5% formalin for 20 min at 37 °C. Cell were then imaged via confocal microscopy using a Nikon A1 Confocal Microscope.

Tail-Vein Injections. For intravenous injection of CRC cells, NSG mice (NOD.Cg-Prkdc Il2rg /SzJ) were anesthetized with isoflurane (induction 4%, maintenance 2%). The viability of cells used for inoculation was >95% as determined by Vi-CELL™ XR (Beckman Coulter). The HT29LuM3-GFP-Luc NTC (*n* = 5) and shCD36 (*n* = 5) cell lines were aliquoted in PBS at a concentration of 1.0 × 10^6^ cells/100 μL and injected via the tail vein into 6–8-week-old mice. Gentle pressure was applied to the inoculation site until there was no visible sign of bleeding. After 9 weeks, bioluminescent imaging using Lago (Spectral Instruments Imaging; Tucson, AZ, USA) was employed to monitor the distribution and development of lung metastasis. Prior to imaging, animals were anesthetized with 2% isoflurane inhalation and images were acquired 10 min after i.p. injection of D-luciferin (150 mg/kg; 100 µL per mouse). Bioluminescent signal was quantified in Aura software (Spectral Instruments Imaging; Tucson, AZ, USA). GFP fluorescence imaging was performed using an LT-9500 Illumatool/TLS (Lightools Research, Encinitas, CA, USA), equipped with an excitation source (470 nm) and filter plate (515 nm).

Cecum Injections. Cecum injections were performed as previously described [42]. HCT116 CD36 overexpression (*n* = 5) and control (*n* = 5) cells were aliquoted in PBS at a concentration of 1.0 × 10^6^ cells/50 μL and injected into the cecal wall of NU/NU mice. After 10 weeks, the mice were sacrificed and gross examination/imaging of the tumor burden within the cecum, colon and intestine was performed.

## 3. Results

### 3.1. CD36 Promotes Invasion and Colony Formation in HCT116 Cells

**Figure 1:**
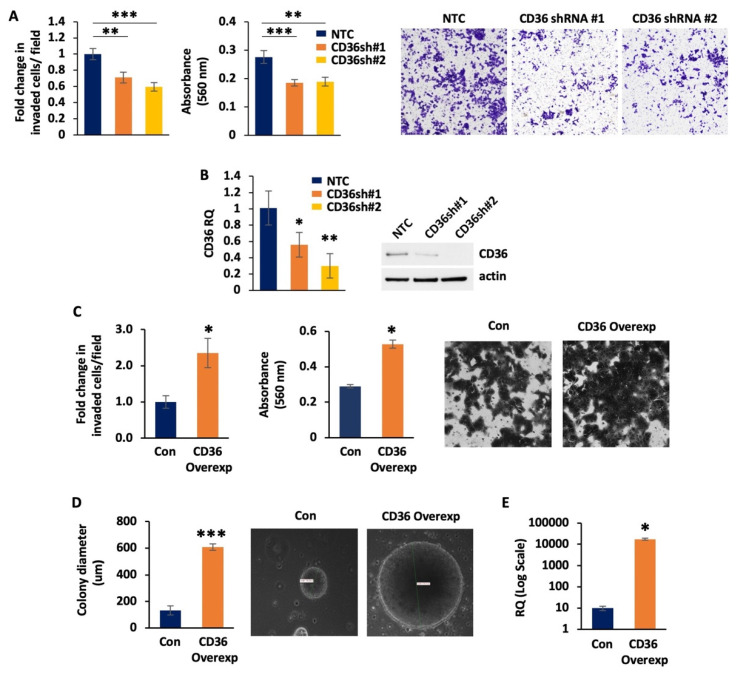
CD36 promotes invasion and colony formation in HCT116 cells. (**A**) Matrigel trans-well invasion assay. Fold change of the invaded cells/field, absorbance at 560 nm of the de-stained Matrigel trans-well invasion chambers and raw images of the HCT116 NTC and CD36 shRNA #1 and #2 cell lines are shown. (**B**) q-RT-PCR and Western blot analyses demonstrating the levels of CD36 knockdown in HCT116 NTC/CD36shRNAs cells. (**C**) Matrigel trans-well invasion assay. Average count of invaded cells/field, absorbance at 560 nm of the de-stained Matrigel trans-well invasion chambers and raw images of the HCT116 p-Lenti-Control and p-Lenti-CD36 overexpression cell lines are shown. (**D**) Quantification and representative images of the HCT116 p-Lenti-Control and p-Lenti-CD36 overexpression soft agar colony formation assays. (**E**) q-RT-PCR demonstrating the levels of CD36 overexpression in the HCT116 p-Lenti-Control and p-Lenti-CD36 overexpression cell lines. All functional assays were performed at least 3 times using multiple replicates. * *p* < 0.05, ** *p* < 0.01, *** *p* < 0.001, SEM.

We have previously shown that CD36 promotes CRC proliferation and survival in vitro and in vivo [15]. Here, we show that shRNA-mediated knockdown of CD36 in HCT116 cells leads to a significant reduction in invasion as compared to non-targeted control HCT116 cells (Figure 1A). Figure 1B shows the mRNA and protein levels of CD36 knockdown in shRNA#1 and shRNA#2 HCT116 cells. In contrast, when CD36 is overexpressed in HCT116 cells, we observe a significant increase in cell invasion and colony formation (Figure 1C,D). Figure 1E shows the mRNA levels of CD36 overexpression in the tested cells.

Together, these data suggest that CD36 promotes invasion and colony formation of CRC cells.

### 3.2. High Expression of CD36 Is Associated with a More Metastatic Phenotype in Isogenic CRC Cell Lines 

**Figure 2:**
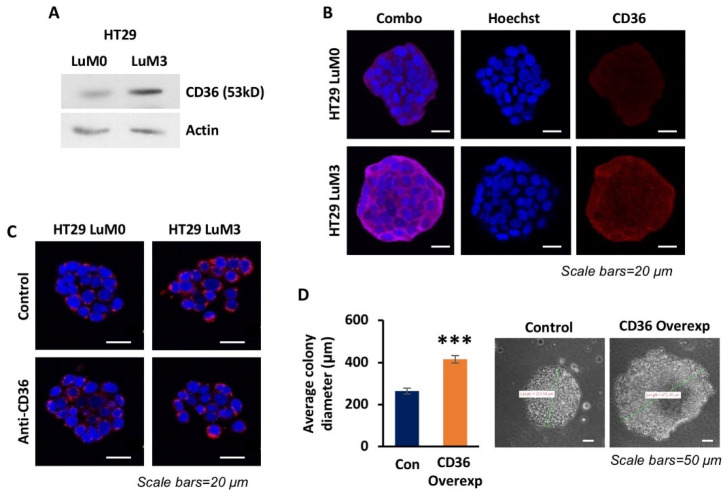
CD36 expression is associated with more metastatic CRC cell lines. (**A**) Western blot analysis of the HT29 LuM0 and LuM3 GFP-Luciferase cell lines. (**B**) Confocal microscopy images of HT29 LuM0 and LuM3 GFP-Luciferase cells for CD36 expression (red), nucleus (blue) and actin filaments (purple). (**C**) Confocal microscopy images of the HT29 LuM0 and LuM3 GFP-Luciferase fatty acid uptake assay control or treated with anti-CD36 blocking antibody for 24 h (lipid analogue—red; DAPI—blue). (**D**) Quantification and representative images of the HT29 LuM0 p-lenti-control and p-lenti-CD36 overexpression soft agar colony formation assays. *N* = 3, *** *p* < 0.001, SEM.

To further investigate any potential role CD36 may play in metastasis of CRC, we utilized the HT29 LuM3-GFP-Luciferase trained cell line, a cell line which was serially injected via the tail vein in mice and has a significantly higher propensity to initiate lung colonies than the parental HT29 cell line [40]. We have previously shown that CD36 is significantly upregulated in the HT29 LuM3 cell line as compared to parental HT29 [15]. Figure 2A,B demonstrate higher CD36 expression in the HT29 LuM3 cell line compared to parental HT29 using Western blot and confocal microscopy, respectively. Additionally, confocal microscopy of fatty acid uptake using BODIPY™ 558/568 C_12_ demonstrates that HT29 LuM3 cells uptake more free fatty acids than parental HT29 cells in a CD36-dependent manner (Figure 2C). Furthermore, overexpressing CD36 in the parental HT29 cells significantly increases colony formation and diameter (Figure 2D). Together these data suggest that CD36 contributes to the metastatic phenotype and an increase in extracellular fatty acid uptake of the HT29 LuM3 cell line [40] and promotes colony formation when overexpressed in HT29 LuM0 cells. 

### 3.3. CD36 Promotes Lung Colonization and Orthotopic Metastasis In Vivo 

**Figure 3:**
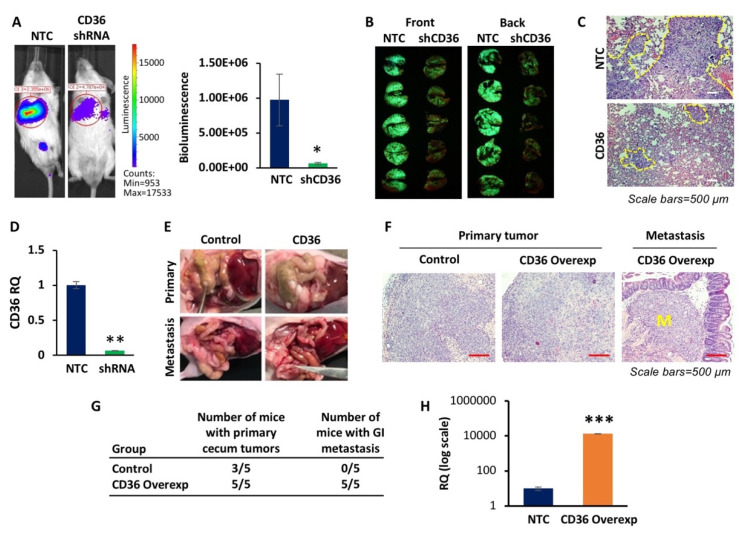
CD36 promotes lung colonization and metastasis in vivo. (**A**) Representative images and quantification of the bioluminescence imaging in tail-vein-injected mice of HT29 LuM3 GFP-Luciferase cells, NTC (*n* = 5) and CD36shRNA (*n* = 5). (**B**) GFP imaging and (**C**) H&E staining of lung tissues of resected lungs of the tail-vein-injected mice. (**D**) qRT-PCR quantification of CD36 in the HT29 LuM3 GFP-Luciferase NTC and CD36sh RNA cell lines. (**E**) Images from the HCT116 control and CD36 overexpression cecum-injected mice, showing increased tumor burden and colon metastasis in the CD36 overexpression mice. (**F**) Hematoxylin and eosin staining of the resected primary and metastatic tissues from mice injected with control and CD36 overexpression HCT116 cells. M—metastasis. (**G**) The rate of occurrence of primary cecum tumor and GI metastasis in mice based on gross examination. (**H**) qRT-PCR quantification of CD36 in the HCT116 p-Lenti-Control and CD36-Overexpression cell lines. * *p* < 0.05, ** *p* < 0.01, *** *p* < 0.001; SEM.

To more thoroughly investigate the role CD36 may play in CRC metastasis, we tested the effect of CD36 on colonization and metastasis in vivo. Utilizing the HT29 LuM3 model, we established the HT29 LuM3-GFP-Luciferase non-targeted control (NTC) and CD36 knockdown (shCD36) cell lines and injected these cells via the tail vein into NU/NU (*n* = 5 each) mice and monitored for lung colony formation. Mice injected with the HT29 LuM3 shCD36 cell line exhibited significantly lower luciferase reporter bioluminescence signaling as compared to mice injected with HT29 LuM3 NTC cells (Figure 3A). Furthermore, resected lungs from both NTC- and shCD36-injected mice show that mice injected with HT29 shCD36 cells have substantially lower GFP signaling and tumor burden than those injected with HT29 NTC (Figure 3B,C). The levels of CD36 mRNA expression in these cells are shown in Figure 3D. 

To better recapitulate the condition of local invasion and metastasis with CRC, we also used the orthotopic cecum injection mouse model. The cecum injection model is a well-established in vivo model that replicates human disease with a higher level of accuracy than other ectopic models [42]. We injected 1 × 10^6^ HCT116 p-Lenti-Control or p-Lenti-CD36 overexpressing cells in 50 µL of PBS into the cecum wall of NU/NU (*n* = 5) mice. Our data shows that mice injected with p-Lenti-CD36 overexpressing HCT116 cells exhibited an increase in the number of primary tumors formed compared to mice injected with p-Lenti-Control cells. More importantly, gross examination of mice identified multiple metastatic nodules in all mice injected with p-Lenti-CD36 overexpressing cells, but not in mice injected with p-Lenti-Control cells (Figure 3E–G). Representative images of the H&E staining of the primary tumors and GI metastasis are shown in Figure 3F. The level of CD36 overexpression in HCT116 cells is shown in Figure 3H.

Together, the in vivo results further support our in vitro data and demonstrate that CD36 promotes metastasis in CRC. 

### 3.4. High Expression of CD36 Increases MMP28 Expression

**Figure 4:**
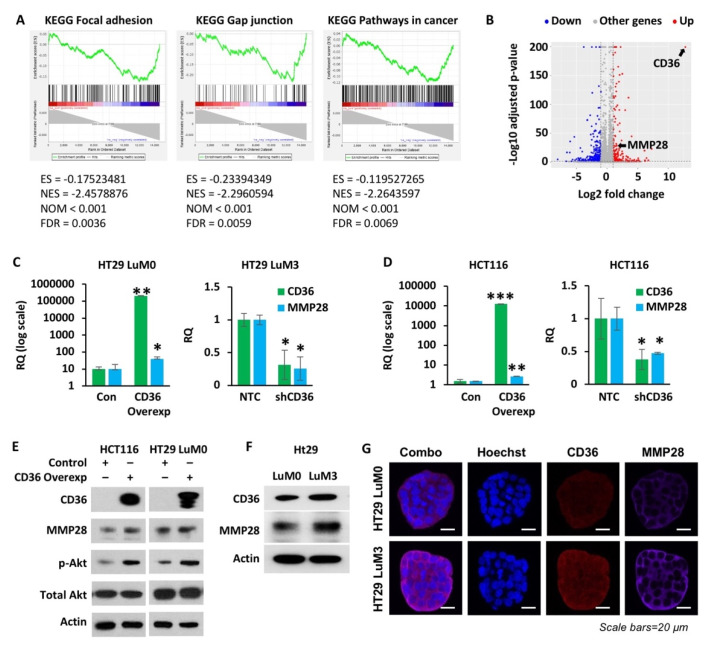
CD36 regulates expression of MMP28. (**A**) Representative gene set enrichment analysis (GSEA) plots generated from RNA-Seq expression data of the HT29 p-Lenti Control and HT29 p-Lenti CD36 overexpression cell lines. The top 10 enriched pathway sets are provided in Figure S2. Bar codes indicate the location of gene set members in the ranked list of all genes. ES, enrichment score; NES, normalized enrichment score; NOM, nominal *p*-value; FDR, false discovery adjusted *p*-value. (**B**) Volcano plot of the HT29 p-Lenti Control and HT29 p-Lenti CD36 overexpression cell lines, showing increased levels of CD36 mRNA associated with an increase in MMP28 mRNA expression. (**C**) qRT-PCR analysis of CD36 and MMP28 mRNA in the HT29, p-Lenti Control and p-Lenti CD36 overexpression cell lines, and in the HT29 LuM3 GFP-Luciferase NTC and shCD36 cell lines. (**D**) qRT-PCR analysis of CD36 and MMP28 mRNA in the HCT116, p-Lenti Control and p-Lenti CD36 overexpression, and HCT116 NTC and CD36 shRNA cell lines. (**E**) Western blot analysis of the HCT116 and HT29 LuM0 p-Lenti Control and p-Lenti CD36 overexpression cell lines for CD36, MMP28, p-Akt and total Akt. (**F**) Western blot analysis of CD36 and MMP28 in HT29 LuM0 and HT29 LuM3 GFP-Luciferase cell lines. (**G**) Confocal microscopy of the HT29 LuM0 and HT29 LuM3 GFP-Luciferase cell lines for actin, CD36 and MMP28. * *p* < 0.05, ** *p* < 0.01, *** *p* < 0.001; SEM.

We have performed RNA-Seq analysis on parental HT29 p-Lenti-Control and p-Lenti-CD36 overexpression cell lines. The full list of DEGs in these two cell lines is included as Figure S1. Gene set enrichment analysis shows an enrichment of genes involving focal adhesion, gap junction and cancer-associated pathways in HT29 p-Lenti-CD36 overexpression cell line as compared to the HT29 p-Lenti-Control (Figure 4A). The top 10 enriched pathway sets are provided in Figure S2. Interestingly, we found that CD36 expression is associated with matrix metallopeptidase 28 (MMP28) (Figure 4B). MMP28 is the newest member matrix metallopeptidase to be identified and is involved in ECM degradation [36]. MMP28 is associated with metastasis in lung and gastric cancer [36,39], but has not yet been studied in CRC. To confirm the RNA-Seq data, we performed qRT-PCR analysis of the HT29 and HCT116 cells (p-Lenti-Control and p-Lenti-CD36 overexpression) and HT29 LuM3 and HCT116 cells (NTC and CD36 shRNA). As shown in Figure 4C, overexpression of CD36 in HT29 cells leads to significant upregulation of MMP28 mRNA. In contrast, shRNA-mediated knockdown of CD36 in HT29 LuM3 cells leads to a decrease in MMP28 mRNA expression. Consistent with these results, HCT116 cells with CD36 overexpression also exhibit higher levels of MMP28 mRNA expression and, inversely, HCT116 CD36 shRNA cells show a significant decrease in MMP28 mRNA expression (Figure 4D). Furthermore, the HCT116 and HT29 LuM0 cell lines with CD36 overexpression also display higher protein expression of MMP28, and the pro-survival marker p-Akt (Figure 4E) [43]. To further support the association between CD36 and MMP28, we show that HT29 LuM3 cells, which express higher levels of CD36, also express higher levels of MMP28 protein compared to the parental HT29 LuM0 cell line, as shown by Western blot and confocal microscopy (Figure 4F,G).

Together these data demonstrate that upregulation of CD36 upregulates expression of MMP28 at the mRNA and protein levels. 

### 3.5. MMP28 Promotes CRC Cell Invasion and Reduces Expression of E-Cadherin In Vitro

Previously published data suggest that MMP28 plays a critical role in the regulation of metastasis in lung and gastric cancers. Specifically, MMP28 has been shown to promote EMT in lung carcinoma and increased expression of MMP28 is associated with a loss of E-cadherin [36,39]. Loss of E-cadherin is a well-established marker for EMT initiation and poor clinical outcome in various cancers, including CRC [26]. 

**Figure 5:**
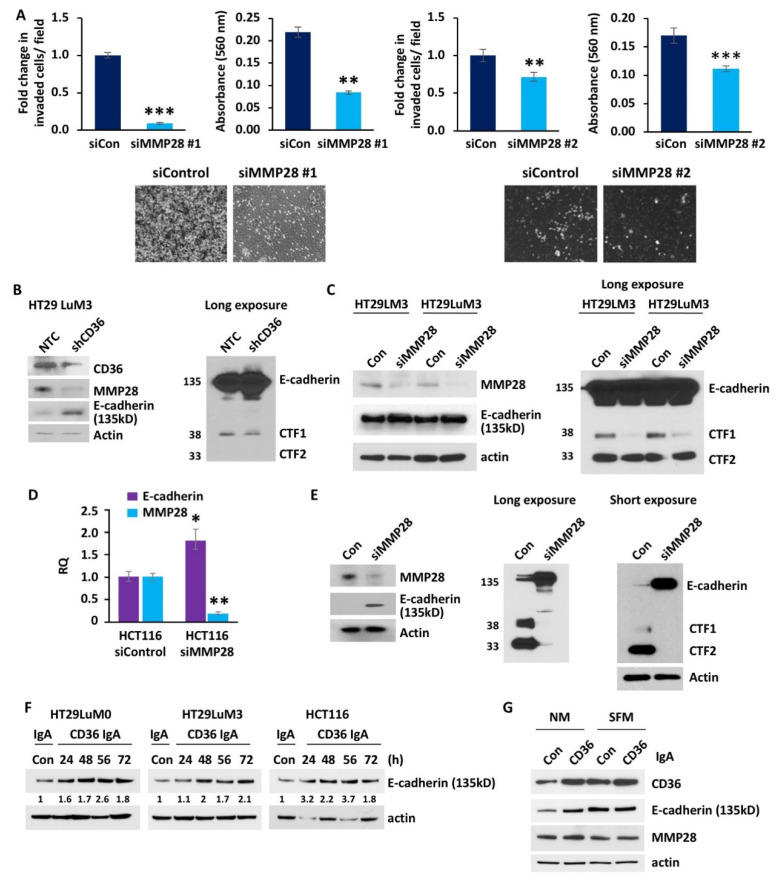
MMP28 reduces CRC cell invasion and decreases expression of functional E-cadherin in vitro. (**A**) Fold change in invaded cells/field, absorbance at 560 nm of the de-stained Matrigel trans-well invasion chambers and raw images of the Matrigel trans-well invasion chambers of the HCT116, siControl and siMMP28 cell lines (*n* = 3). (**B**) Western blot analysis of the HT29 LuM3 NTC and shCD36 cell lines for CD36, MMP28 and E-cadherin. The long exposure of the same Western blot for E-cadherin is also shown. (**C**) Western blot for MMP28 and E-cadherin expression in HT29LM3 and HT29LuM3 cells. Western blot including the E-cadherin cleavage products CTF1 and CTF2 is shown on a separate blot (long exposure). (**D**) qRT-PCR analysis of the control and MMP28 siRNA transfected HCT116 cell lines for MMP28 and E-cadherin. (**E**) Western blot for MMP28 and E-cadherin expression in HCT116 cells. Western blot including the E-cadherin cleavage products CTF1 and CTF2 is shown on separate blots (long and short exposure). (**F**) Western blot analysis showing the effect of CD36 blocking antibody on E-cadherin expression in HT29 LuM0, HT29LuM3 and HCT116 cells. Expression of E-cadherin is quantified based on band intensity and normalized to actin. (**G**) Western blot analysis showing the effect of CD36 blocking antibody on e-cadherin and MMP28 expression in HCT116 cells treated with IgA or IgCD39 for 48 h in normal medium or SFM (* *p* < 0.05, ** *p* < 0.01, *** *p* < 0.001).

To further elucidate the role of MMP28 in CRC metastasis, we established a transient knockdown of MMP28 in HCT116 cells using two different MMP28 siRNAs. HCT116 siMMP28 #1 and siMMP28 #2 cells display a significantly lower ability to invade across a Matrigel trans-well as compared to control cells transfected with scrambled siRNA (Figure 5A). Furthermore, a decrease in MMP28 mRNA expression due to transfection with MMP28 siRNA leads to a significant increase in mRNA expression of E-cadherin when compared to cells transfected with scrambled siRNA control (Figure 5B). Western blot analysis of the HT29-LuM3-GFP-Luciferase NTC and CD36 shRNA knockdown cell lines demonstrates a reduction in MMP28 expression and an increase in E-cadherin expression (135 kD) (Figure 5C). Additionally, this analysis shows a decrease in the product of E-cadherin cleavage, CTF1(38kD), in the CD36 knockdown cell line (Figure 5C). siRNA-mediated knockdown of MMP28 in HT29LM3 cells trained to metastasize to liver [44] and HT29LuM3 cells also leads to upregulation of E-cadherin expression and a decrease in CTF1 (Figure 5D). To further investigate the effect of MMP28 on E-cadherin cleavage, we performed Western blot analysis of HCT116 cells transfected with MMP28 siRNA versus HCT116 control cells. We found that MMP28 is not only associated with a decrease in functional E-cadherin (135 kD) but also associated with an increase in two cleavage products of E-cadherin, CTF1 and CTF2 (33 kD). Inhibition of MMP28 expression is associated with almost complete abolishment of E-cadherin cleavage (Figure 5D), suggesting that MMP28 plays a crucial role in the regulation of E-cadherin, potentially via direct cleavage. These data further support the hypothesis that MMP28 regulates expression of E-cadherin downstream of CD36 and indicates MMP28 may be involved in direct cleavage of E-cadherin. 

The levels of E-cadherin expression can be regulated transcriptionally or by E-cadherin cleavage and shedding [33]. We have not identified any modes of transcriptional regulation, such as through the E-cadherin regulators Slug or Snail [32,33], in response to altered expression of CD36 or MMP28.

To confirm that CD36 regulates E-cadherin via FA uptake, we treated HT29LuM0, HT29LuM3 and HCT116 cells with anti-CD36 blocking antibody. Inhibition of exogenous FA uptake increased the expression of E-cadherin starting at 24 h treatment in HT29LuM0 and HCT116 cells and 48 h treatment in HT29LuM3 cells (Figure 5F). We have previously shown that exposure of CRC cells to serum-free medium (SFM) upregulates CD36 expression [15]. Here, we show that both SFM and anti-CD36 blocking antibody treatment of HCT116 cells upregulates CD36 expression. However, blocking of CD36 with antibody upregulates the expression of E-cadherin in normal medium, but not in SFM (Figure 5G), further suggesting that the effect of CD36 on E-cadherin expression is FA uptake dependent. No significant changes in MMP28 expression were observed. 

Together, these data suggest that CD36 increases the invasive and metastatic potential of CRC cells via induction of MMP28 and transcriptional downregulation and cleavage of E-cadherin. 

### 3.6. Overexpression of CD36 Is Associated with an Increase in MMP28 Expression and Reduction of E-Cadherin In Vivo and Human CRC Specimens 

**Figure 6:**
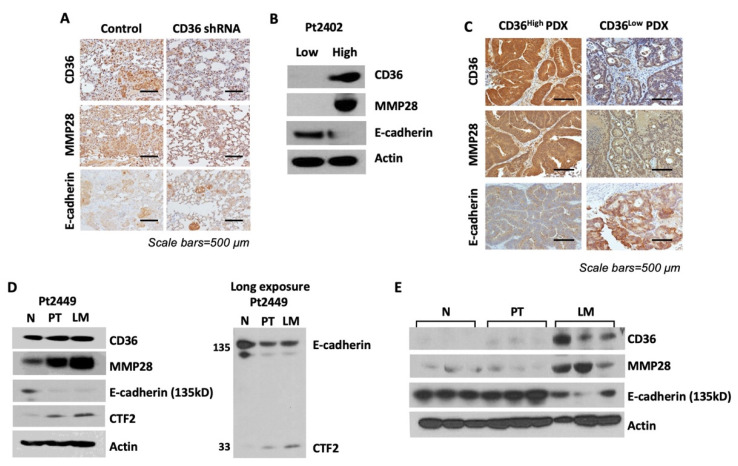
Overexpression of CD36 is associated with an increase in MMP28 expression and reduction in the level of E-cadherin in vivo and human CRC specimens. (**A**) IHC analysis of the tissues from tail-vein injection of the HT29 LuM3 GFP-Luciferase NTC and shCD36 cell lines shown in Figure 3A. (**B**) Western blot analysis and (**C**) IHC analysis of the tissues from Pt2402 PDX, CD36 low and CD36 high, the isogenic tumors for CD36, MM28 and E-cadherin. (**D**) Western blot analysis of matched normal (N), primary tumor (PT) and liver metastasis (LM) tissues for CD36, MMP28, E-cadherin and CTF2. (**E**) Western blot analysis of CD36, MMP28 and E-cadherin in unmatched N, PT and LM patient tissues.

To support our data on the CD36–MMP28–E-cadherin axis in CRC metastasis, we analyzed the expression of CD36, MMP28 and E-cadherin in lung tissues resected from mice injected with HT29 LuM3, NTC and CD36 shRNA cells (Figure 3A). IHC analysis demonstrates that a reduction in CD36 and MMP28 expression is associated with an increase in E-cadherin expression in tumor lesions with CD36 knockdown, as compared to NTC tumor tissues (Figure 6A). 

Our laboratory has previously shown that isogenic patient-derived xenograft (PDX) tumors, which were established from isolating tumor cells expressing high levels of CD36 (CD36^high^), have a higher propensity to grow subcutaneous tumors in vivo [15]. To further confirm the relationship between CD36, MMP28 and E-cadherin, we analyzed tissues from Pt2402 CD36^high^ and CD36^low^ PDXs. Western blot analysis and IHC staining confirms that low expression of CD36 is associated with low expression of MMP28 and an increase in the level of E-cadherin (Figure 6B,C). 

To translate our in vitro and in vivo findings to human tissues, we analyzed the expression of CD36, MMP28 and E-cadherin in matched normal mucosa, primary CRC and liver metastasis specimens. Our data show that as we transition from normal tissue to a primary tumor and then metastasis, we see an increase in the expression of both CD36 and MMP28 (Figure 6D). Furthermore, an analysis of matched normal mucosa, primary CRC and CRC liver metastasis demonstrates and an increase in expression of CD36 and MMP28 and a decrease in the expression of E-cadherin as we move from normal tissues to primary and metastatic tumors. In agreement with our in vitro data, these human tissues also show an increase in the cleavage product of E-cadherin, CTF2, as disease progresses, suggesting that MMP28, through CD36 regulation, may directly cleave E-cadherin (Figure 6D,E).

In summary, our data suggest that CD36 upregulates MMP28 expression, inducing E-cadherin loss in vivo and in human tissues.

## 4. Discussion

Previous investigation from our laboratory has revealed that CD36 plays an important role in the survival and proliferation of CRC cells and its specific upregulation may be a potential compensation mechanism when de novo fatty acid synthesis is inhibited in CRC [15]. Aside from its pro-survival and tumorigenic properties, CD36 has been implied in the promotion of invasion and metastasis of multiple types of cancer [11,13,16,17,18]. Despite this knowledge, the role of CD36 in the process of invasion and metastasis of CRC has not yet been investigated.

In parallel with previous reports that suggest CD36 promotes invasion and metastasis of ovarian and gastric cancer cells, we found that CD36 promotes colony formation and trans-well invasion of CRC cells in vitro [11,17]. Consistent with these findings, we show that CRC cells, which exhibit a more metastatic phenotype, express higher levels of CD36 and uptake more exogenous fatty acids in a CD36-dependent fashion. 

One of the premier studies investigating the role of CD36 in metastasis was that of Pascual et al., which described the pro-metastatic characteristic of CD36 in oral carcinoma. It was reported that a subpopulation of oral carcinoma cells that express high levels of CD36 were unique in their ability to initiate metastasis in vivo [16]. Additionally, they found that knockdown or inhibition of CD36 did not affect oral carcinoma primary xenograft tumor growth, but almost completely abolished local invasion [16]. In contrast to their findings, our previous study showed that CD36 does play an important role in primary CRC tumor growth, in particular when endogenously synthesized fatty acids are limited [15]. However, our current study is in agreement with their findings on metastasis, suggesting that CD36 is also critical for CRC invasion and metastasis. Consequently, our findings here are also in parallel with several other studies showing that CD36 promotes metastasis in vivo [11,13,16,17]. 

It is worth noting that due to the role of CD36 in the survival and proliferation of CRC cells, it is possible that this may also be responsible for the effect of CD36 on CRC metastasis. Therefore, it is critically important to identify a potential mechanistic pathway that distinguishes the pro-survival characteristics of CD36 from any pro-metastatic ones. Previously we described that CD36 is associated with increased levels of the pro-survival marker survivin in primary CRC [15]. We also showed that an increase in the expression of CD36 increases phosphorylation of Akt [15]. The Akt pathway is implicated in regulation of both cancer cell survival and metastasis [43]. A previously published study has implicated that CD36 promotes metastatic disease in gastric cancer via the Akt/GSK-3β/β-catenin pathway [17]. Consistent with this study, we show that overexpressing CD36 increases cell invasion and colony formation and is associated with increased levels of p-Akt in established CRC cell lines. However, an increase in phosphorylation of Akt alone is not sufficient to describe a metastatic advantage for CRC. 

As previously mentioned, the process of EMT is recognized as one of the crucial steps involved in cancer metastasis and a hallmark of cancer [22,23,45]. The remodeling of the extracellular matrix is critical for cells to escape their primary environment and enter the systemic circulation (blood or lymphatic system) where they then travel to distant sites and colonize new metastatic tumors [22]. It has been previously shown that increased fatty acid uptake via CD36 is associated with EMT progression in hepatocellular carcinoma cells [46]. Furthermore, CD36 has been shown to promote EMT in cervical cancer through interactions with the TGF-β pathway [18]. More specifically, treatment of cervical cancer cells with TGF-β increased CD36 expression and exhibited a significant loss in the expression of E-cadherin [18]. Loss of E-cadherin is a critical marker for EMT and a well-established hallmark of cancer [22]. Interestingly, our studies show that overexpression of CD36 promotes loss of E-cadherin through a relatively novel member of the MMP family of proteins, MMP28. 

A few members of the MMP family of proteins have been linked to EMT in multiple diseases [47,48,49]. Particularly, MMP-3 and 7 have been shown to promote EMT in breast cancer cells in vitro through direct cleavage of E-cadherin [47,49]. In most studies, those tumors expressing higher levels of various MMPs result in a poor prognosis for patient survival [48]. MMP28 is associated with EMT in lung carcinoma [36]. Additionally, MMP28 is associated with invasion and colony formation in gastric cancer cells and increased MMP28 expression is a poor prognostic factor for gastric cancer patients [37,38]. Our studies show here, for the first time, that overexpression of CD36 increases MMP28 expression and that MMP28 promotes CRC cell invasion. We also show here that expression of both CD36 and MMP28 is associated with a loss of the critical EMT marker E-cadherin in CRC cell lines. Along with this loss of E-cadherin, MMP28 expression is associated with increased levels of the cleaved C-terminal fragments of E-cadherin—CTF1 and CTF2 [50]. CTF1 contains both the transmembrane and cytoplasmic domains of E-cadherin and is produced after cleavage [51]. CTF1 can then be processed further, resulting in the release of CTF2, which is just the cytoplasmic domain of E-cadherin [50,51]. Our data show that MMP28-mediated E-cadherin cleavage is cell-type dependent and seems to be more prominent in HCT116 cells. 

Aside from simply being cleavage products of E-cadherin, the CTFs themselves can act as downstream signaling molecules, such as interacting with and preventing β-catenin degradation as well as aiding in the translocation of β-catenin to the nucleus, promoting transcription of various downstream genes [52]. Because of the prominent role the Wnt/β-catenin pathway plays in CRC, further investigation into the MMP28–E-cadherin–CTF association described here may yield greater information on the mechanisms of CRC progression and metastasis.

Clinically, obesity has been linked to increased risk of metastasis in many cancer types, including CRC [53]. However, the link between high-fat diet, obesity and metastasis is not well understood. A 2017 study showed that dietary lipids enhanced the ability of the CD36 subpopulation of oral carcinoma cells to metastasize and treatment with anti-CD36 neutralizing antibodies inhibited metastasis initiation and induced significant regression of established oral carcinoma metastases [16]. Consistent with these data, using anti-CD36 neutralizing antibody, we show that the effect of CD36 on E-cadherin loss is, at least in part, mediated by FA uptake. However, we also noted that blocking of CD36 with antibody did significantly impact expression of MMP28 and E-cadherin shedding as compared to CD36 and MMP28 downregulation via shRNA and siRNA approaches, respectively. The potential explanation for this is that CD36 regulates MMP28 primarily via intracellular mechanisms independent of its function as a FA transporter. Indeed, several studies showed that besides functioning on the membrane as a FA transporter, CD36 may play other roles such as facilitating intracellular traffic of FAs and increasing the rate of intracellular esterification and, thus, regulating cellular signaling and metabolism without catalyzing the translocation of FAs across the plasma membrane [54,55,56]. To support the intracellular role of CD36, our published work shows a significant increase in cytosolic CD36 staining in tumor tissues as compared to normal mucosa [15]. 

Results of the current study show that CD36 regulates E-cadherin through both transcriptional regulation and proteolytic processing via MMP28, suggesting that upregulation of MMP28 expression by overexpression of CD36 followed by E-cadherin shedding may be independent of FA uptake via CD36. CD36-targeted therapies, including CD36 neutralizing antibodies, have been in development, but knowledge gaps about the multiple roles of this protein in cancer cells as well as in the tumor microenvironment must be filled before further advancements can be made towards clinical practice [57]. Furthermore, our results presented in the current manuscript warrant additional mechanistic studies to delineate FA uptake dependent and independent of CD36 on metastasis. Another limitation of the current study is that the direct effect of a CD36-blocking antibody was not tested in functional assays or in vivo. We plan to delineate the link between high-fat diet, CD36-mediated uptake of FA uptake and CRC metastasis in our future studies.

Aside from showing a direct relationship between MMP28 and E-cadherin, we more importantly show that MMP28 downregulation via reduced expression of CD36 significantly upregulates E-cadherin, not only in established CRC cell lines, but also in isogenic PDXs and patient tissues. To our knowledge, this is the first study to demonstrate that CD36 upregulates MMP28 expression and loss of E-cadherin. Our study is also the first to suggest that MMP28 may be directly involved in the cleavage of E-cadherin in CRC. Therefore, the data presented here show that CD36 might play an important role in the regulation of metastasis in CRC through the upregulation of MMP28 and loss of E-cadherin. 

## 5. Conclusions

Late-stage CRC, categorized by advanced disease with distant metastasis, remains one of the deadliest cancers in the United States and indeed the world [1]. New therapeutic strategies to identify and target tumor cells with higher metastatic potential are needed to improve the efficacy of treatment and increase overall patient survival. Together, this study highlights the potential of both CD36 and MMP28 as therapeutic targets for CRC. Further investigation into the exact mechanistic regulation of MMP28 by CD36 and MMP28’s interaction with E-cadherin is needed to potentially develop new, more effective treatment strategies for patients with late-stage CRC or other solid malignancies.

## Data Availability

All data included in the manuscript or in the supplemental material are available upon request.

## Supplementary Materials

The following supporting information can be downloaded at: https://www.mdpi.com/article/10.3390/cancers14010252/s1, Figure S1: List of DEGs in HT29 control vs. HT29 CD36 overexpression cells, Figure S2: Positively and negatively enriched pathways in HT29 control vs. HT29 CD36 overexpression cells.
