# Supplementary material for: Upregulation of CD36, a Fatty Acid Translocase, Promotes Colorectal Cancer Metastasis by Increasing MMP28 and Decreasing E-Cadherin Expression
Source: Cancers (Basel). 2022 Jan 5;14(1):252. doi: 10.3390/cancers14010252 (PMC8750155; doi:10.3390/cancers14010252)
Supplement: Supplementary file 1 [file cancers-14-00252-s001.zip › Figure S1.pdf]

My Notebook
Manuscripts/CD36 Metastasis Paper 7-12-21/Westerns

PDF Version generated by

Yekaterina Zaytseva (yyzayt2@uky.edu)

on

Jul 22, 2021 @02:19 PM EDT

Table of Contents

HCT116 HT29 LuM0 CD36 Over 12-8-20 2
HCT116 HT29 LuM0 CD36 Over 12-18-20 5
HCT116 siMMP28 3-25-21 8
HCT116 siMMP28 6-7-21 11
HCT116 siMMP28 e-cad cleaved 6-8-21 18
HT29 LuM0 LuM3 4-22-21 25
LuM0 LuM3 3-1-21 28
LuM3 shCD36 5-4-21 30
Pt 2449 Matched Tissues 6-15-21 35
Pt 2377 Matched Tissues 6-27-21 39
Pt 2402 CD36 +/- 6-15-21 49
Pt 2402 CD36 +/- 1-29-21 52
Tissues with Pt 2377 6-29-21 56

HCT116 HT29 LuM0 CD36 Over 12-8-20

James Drury (jmdr236@uky.edu) - Jul 12, 2021, 12:07 PM EDT

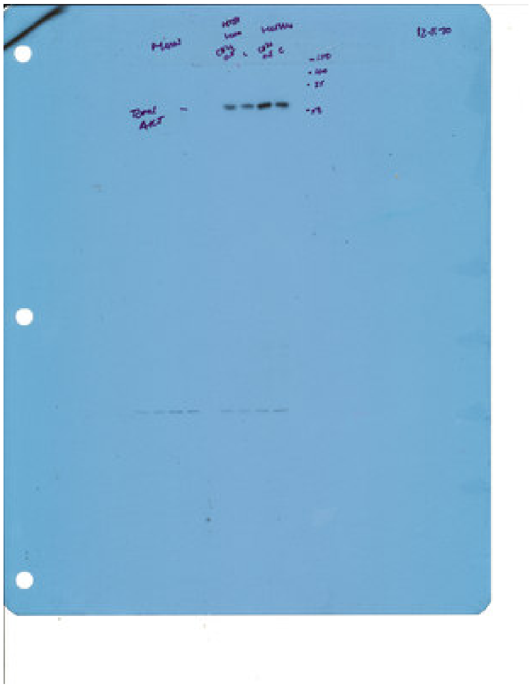

HCT116_HT29_CD36_Over_12-8-2020_0001.jpg(1.1 MB) - download

James Drury (jmdr236@uky.edu) - Jul 12, 2021, 12:07 PM EDT

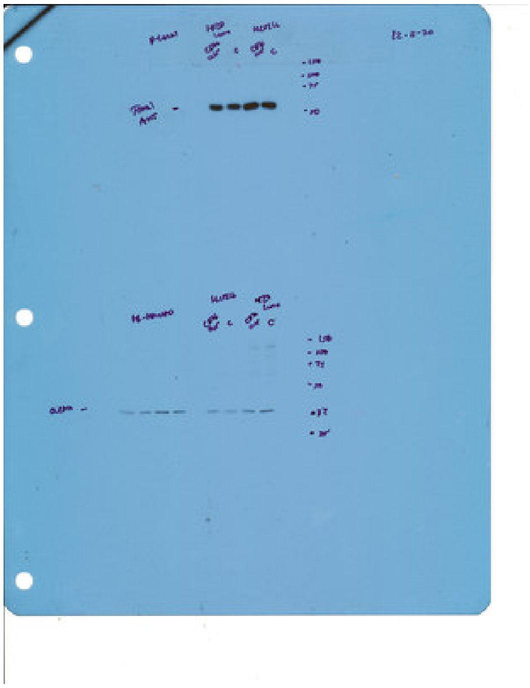

HCT116_HT29_CD36_Over_12-8-2020_0002.jpg(1.2 MB) - download

James Drury (jmdr236@uky.edu) - Jul 12, 2021, 12:07 PM EDT

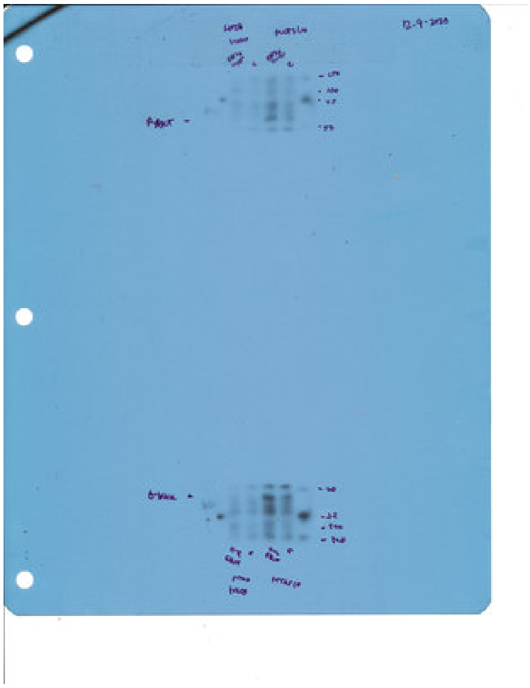

HCT116_HT29_CD36_Over_12-8-2020_0003.jpg(1.2 MB) - download

James Drury (jmdr236@uky.edu) - Jul 12, 2021, 12:07 PM EDT

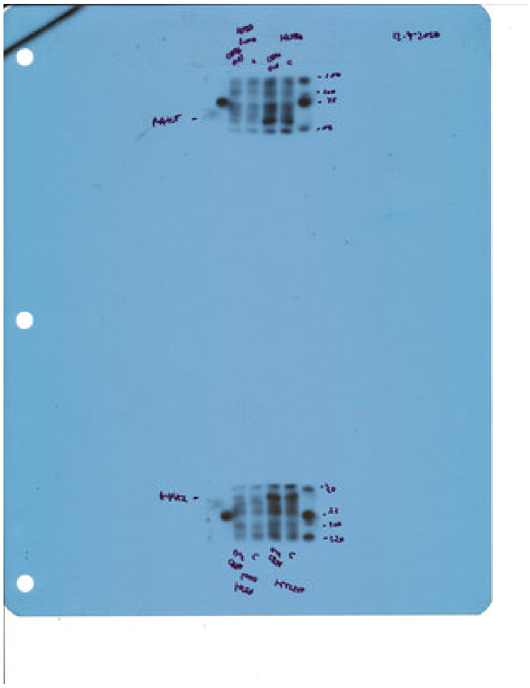

HCT116_HT29_CD36_Over_12-8-2020_0004.jpg(1.2 MB) - download

James Drury (jmdr236@uky.edu) - Jul 12, 2021, 12:08 PM EDT

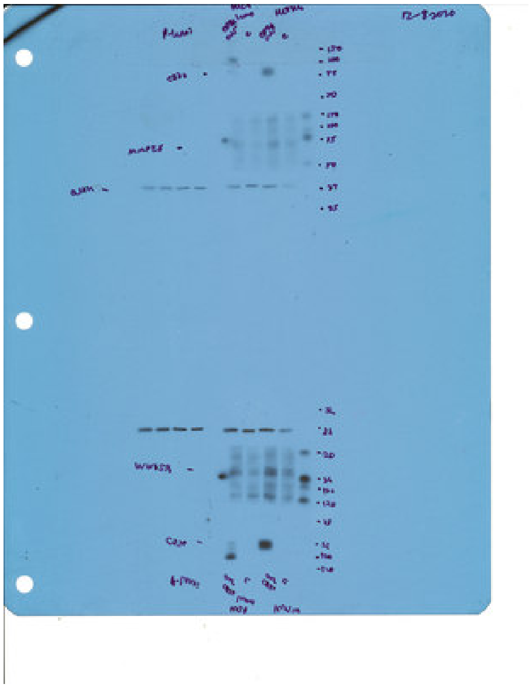

HCT116_HT29_CD36_Over_12-8-2020_0005.jpg(1.2 MB) - download

James Drury (jmdr236@uky.edu) - Jul 12, 2021, 12:08 PM EDT

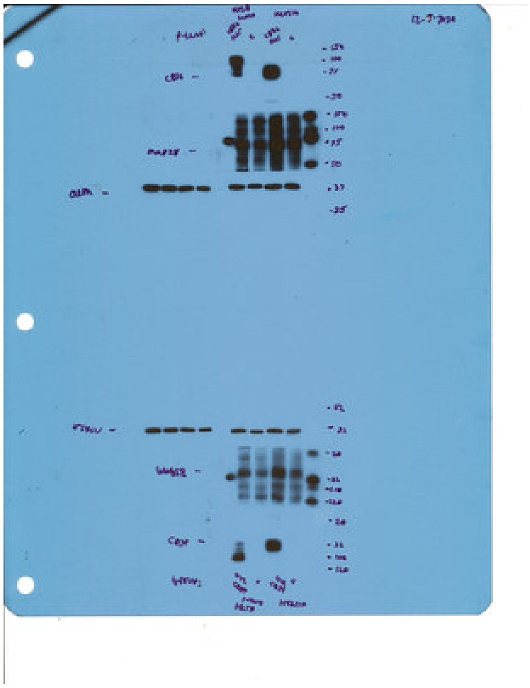

HCT116_HT29_CD36_Over_12-8-2020_0006.jpg(1.2 MB) - download

HCT116 HT29 LuM0 CD36 Over 12-18-20

James Drury (jmdr236@uky.edu) - Jul 12, 2021, 12:09 PM EDT

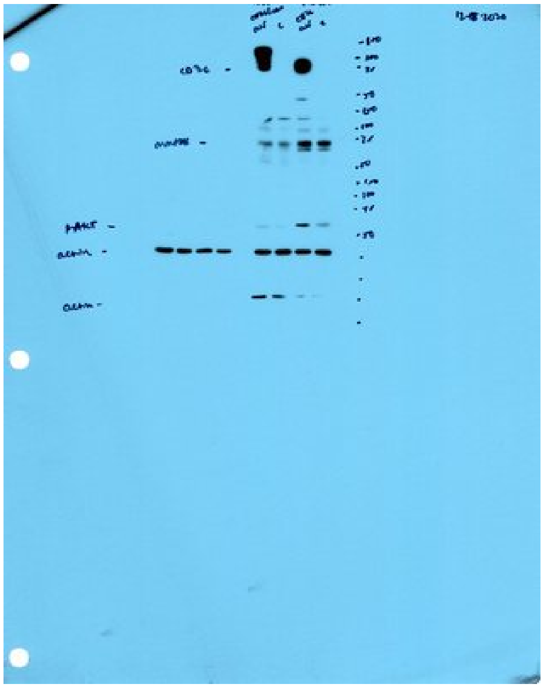

img12182020_002.jpg(663.3 KB) - download

James Drury (jmdr236@uky.edu) - Jul 12, 2021, 12:09 PM EDT

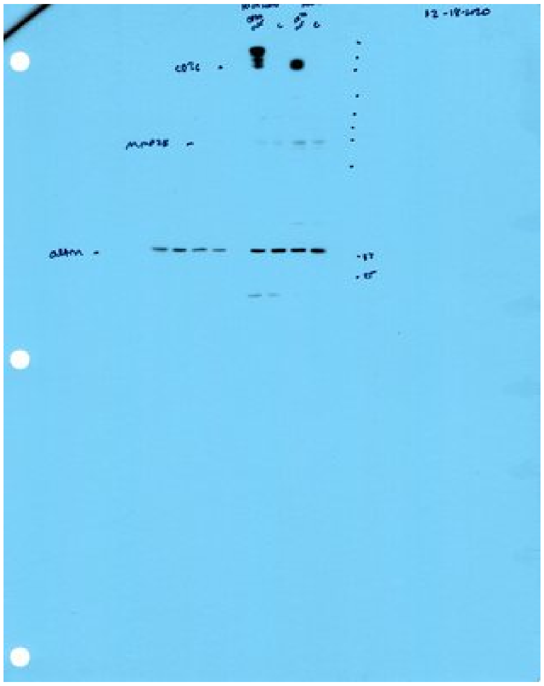

img12182020_003.jpg(653.8 KB) - download

James Drury (jmdr236@uky.edu) - Jul 12, 2021, 12:09 PM EDT

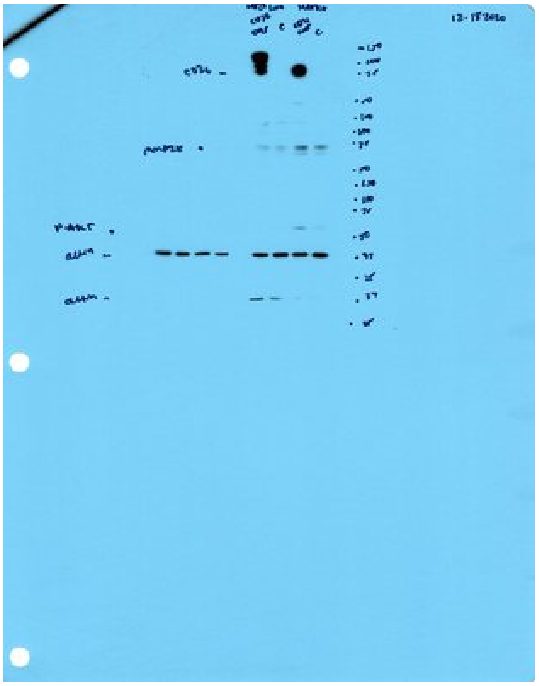

img12182020_004.jpg(631.7 KB) - download

James Drury (jmdr236@uky.edu) - Jul 12, 2021, 12:09 PM EDT

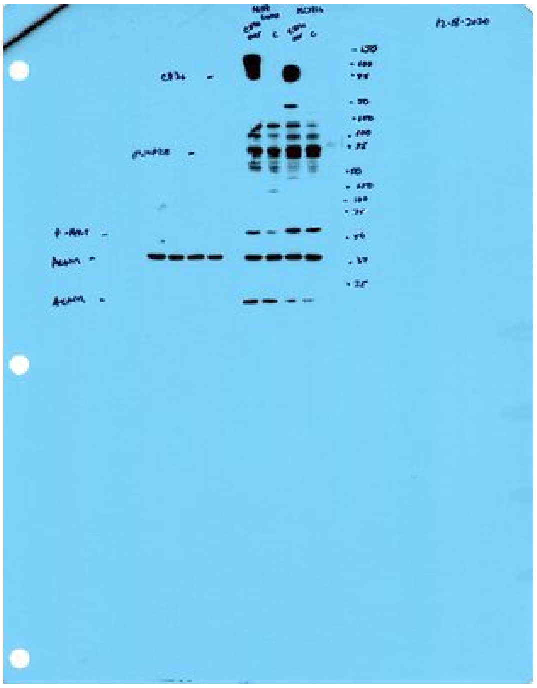

img12182020_005.jpg(632.9 KB) - download

James Drury (jmdr236@uky.edu) - Jul 12, 2021, 12:09 PM EDT

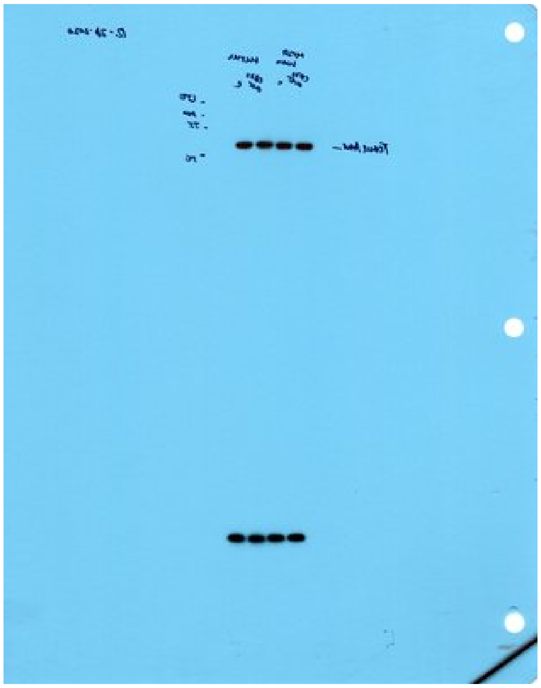

img12212020_006.jpg(813.3 KB) - download

HCT116 siMMP28 3-25-21

James Drury (jmdr236@uky.edu) - Jul 12, 2021, 12:13 PM EDT

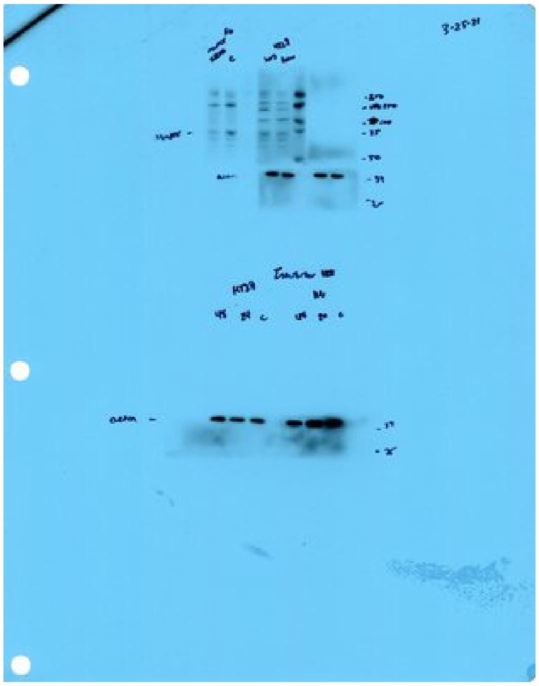

img03252021_040.jpg(527.4 KB) - download

James Drury (jmdr236@uky.edu) - Jul 12, 2021, 12:13 PM EDT

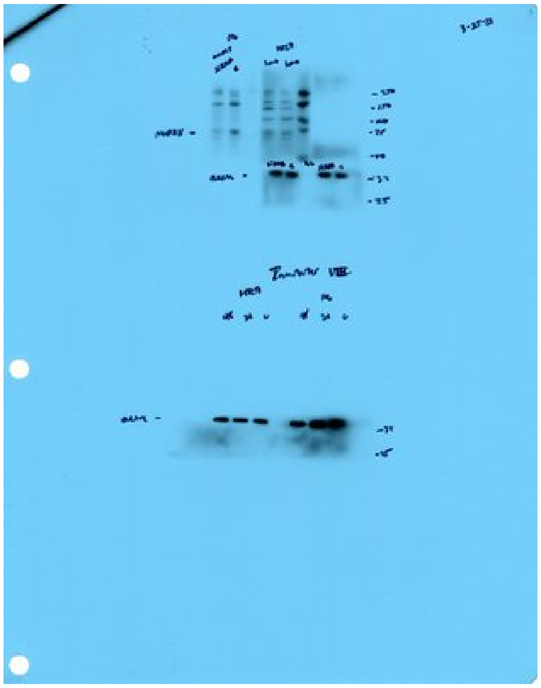

img03252021_041.jpg(662.8 KB) - download

James Drury (jmdr236@uky.edu) - Jul 12, 2021, 12:13 PM EDT

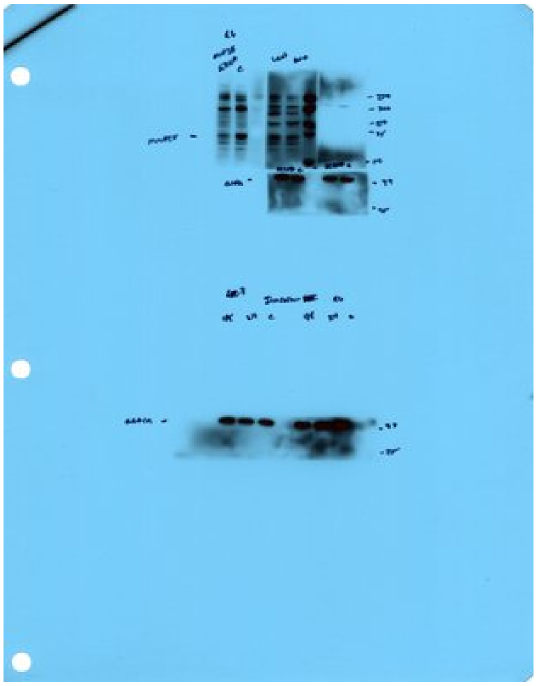

img03252021_042.jpg(496 KB) - download

James Drury (jmdr236@uky.edu) - Jul 12, 2021, 12:13 PM EDT

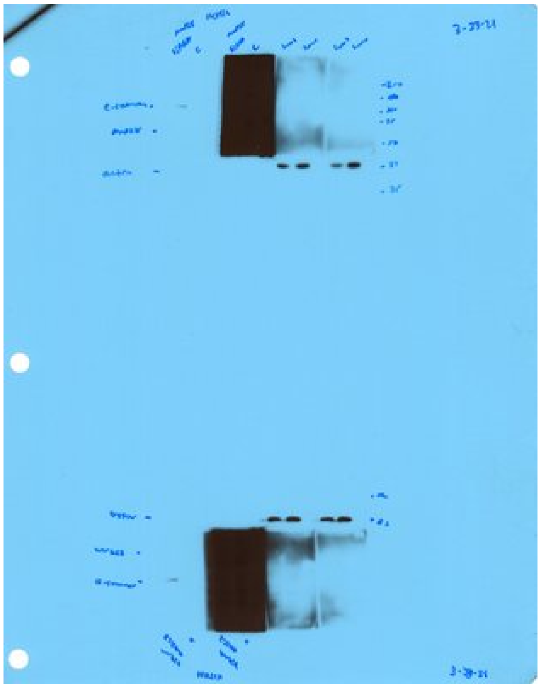

img03252021_043.jpg(624.7 KB) - download

James Drury (jmdr236@uky.edu) - Jul 12, 2021, 12:13 PM EDT

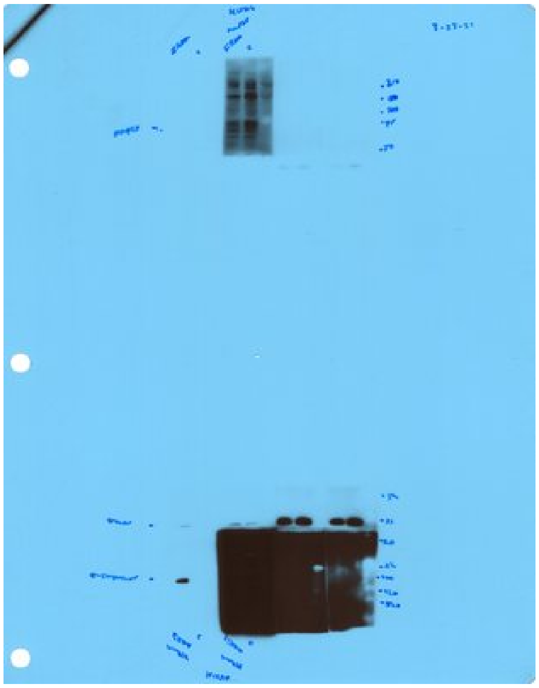

img03252021_044.jpg(449.8 KB) - download

James Drury (jmdr236@uky.edu) - Jul 12, 2021, 12:13 PM EDT

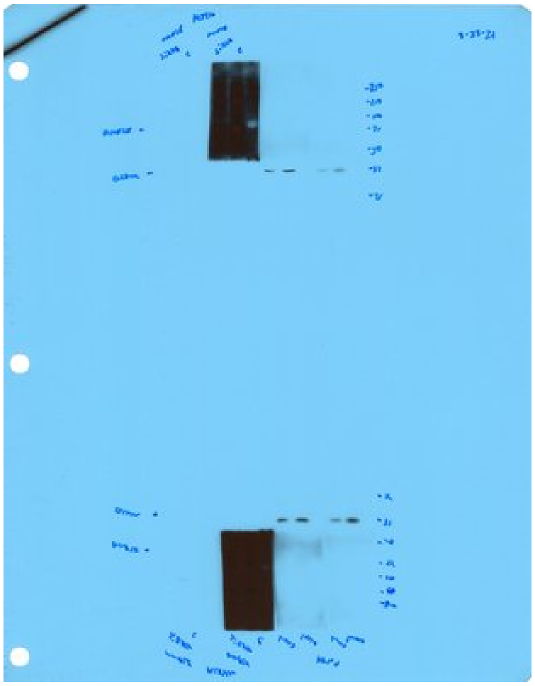

img03252021_045.jpg(498.5 KB) - download

HCT116 siMMP28 6-7-21

James Drury (jmdr236@uky.edu) - Jul 12, 2021, 12:13 PM EDT

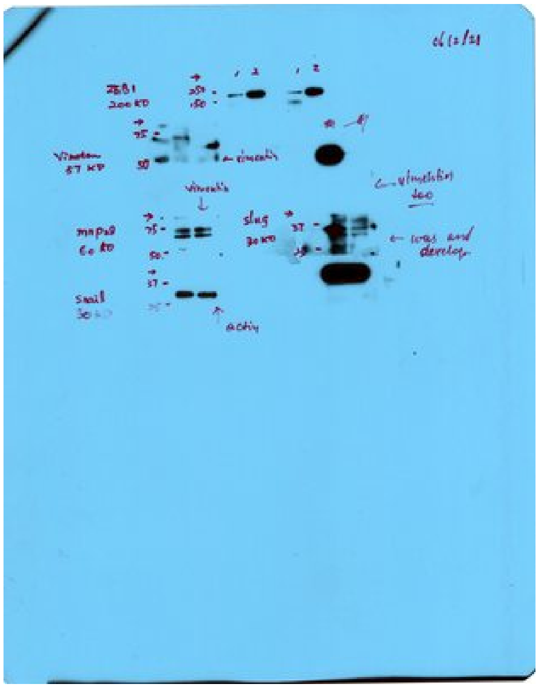

img06082021_096.jpg(562.5 KB) - download

James Drury (jmdr236@uky.edu) - Jul 12, 2021, 12:13 PM EDT

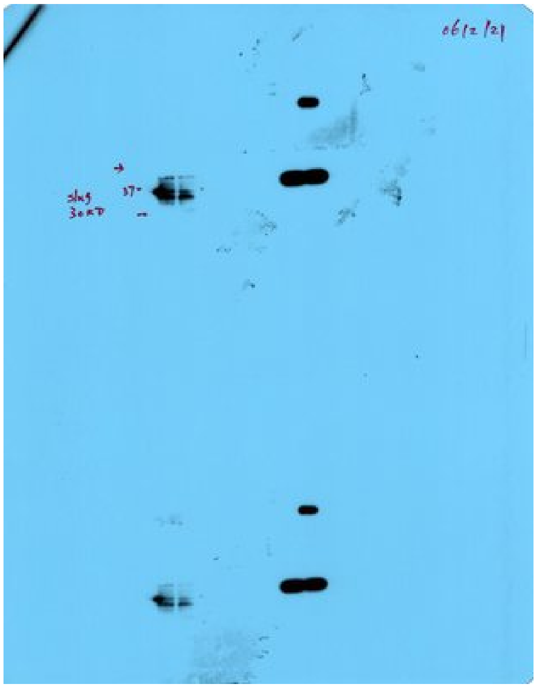

img06082021_097.jpg(530.5 KB) - download

James Drury (jmdr236@uky.edu) - Jul 12, 2021, 12:13 PM EDT

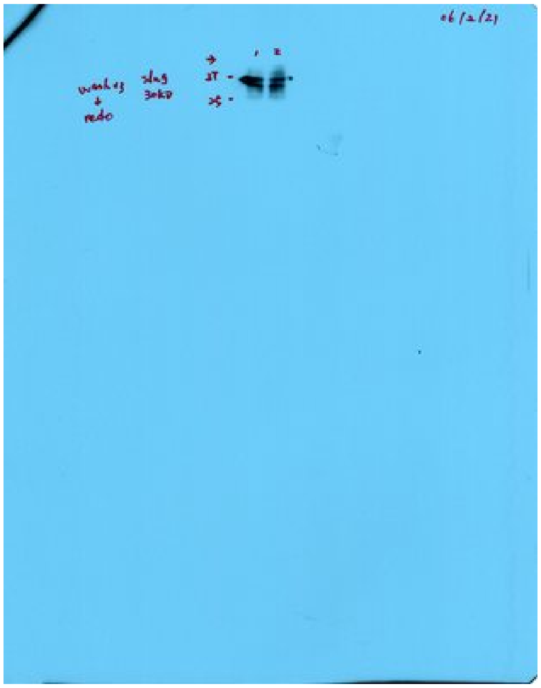

img06082021_098.jpg(679.8 KB) - download

James Drury (jmdr236@uky.edu) - Jul 12, 2021, 12:13 PM EDT

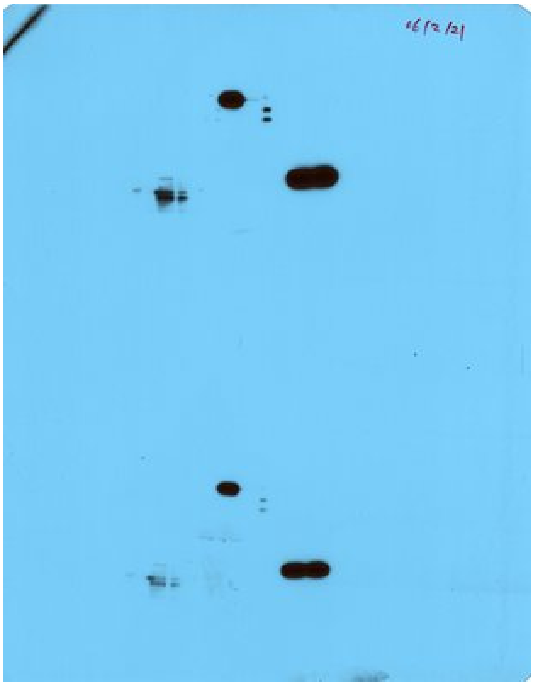

img06082021_099.jpg(499.2 KB) - download

James Drury (jmdr236@uky.edu) - Jul 12, 2021, 12:13 PM EDT

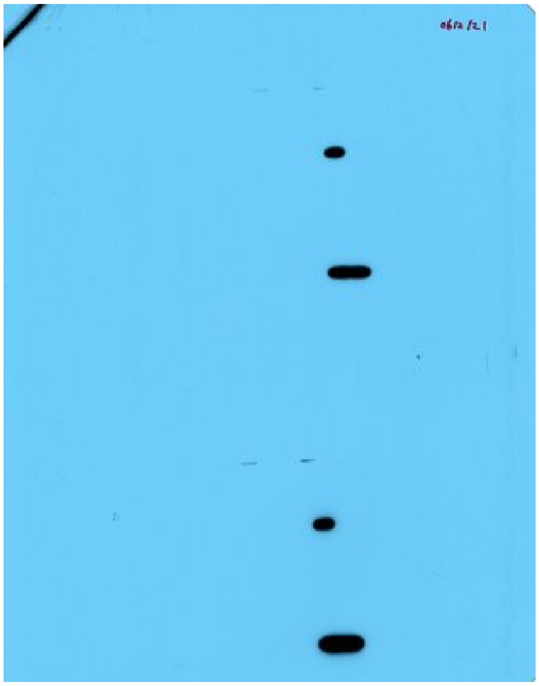

img06082021_100.jpg(549 KB) - download

James Drury (jmdr236@uky.edu) - Jul 12, 2021, 12:13 PM EDT

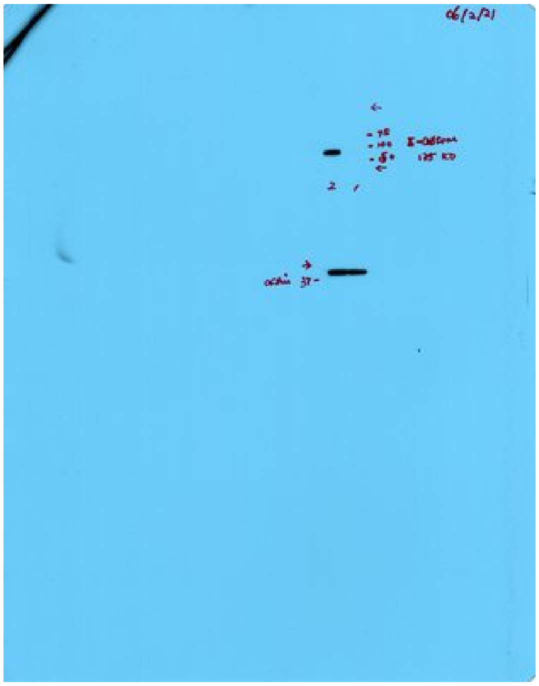

img06082021_101.jpg(547.8 KB) - download

James Drury (jmdr236@uky.edu) - Jul 12, 2021, 12:13 PM EDT

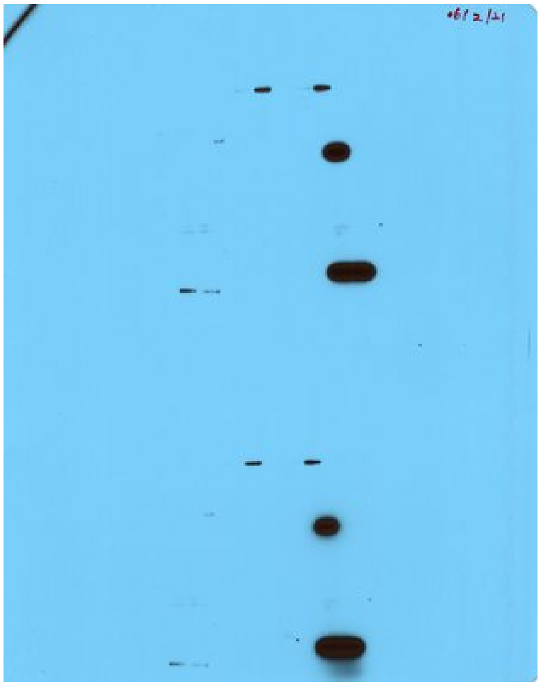

img06082021_102.jpg(497 KB) - download

James Drury (jmdr236@uky.edu) - Jul 12, 2021, 12:13 PM EDT

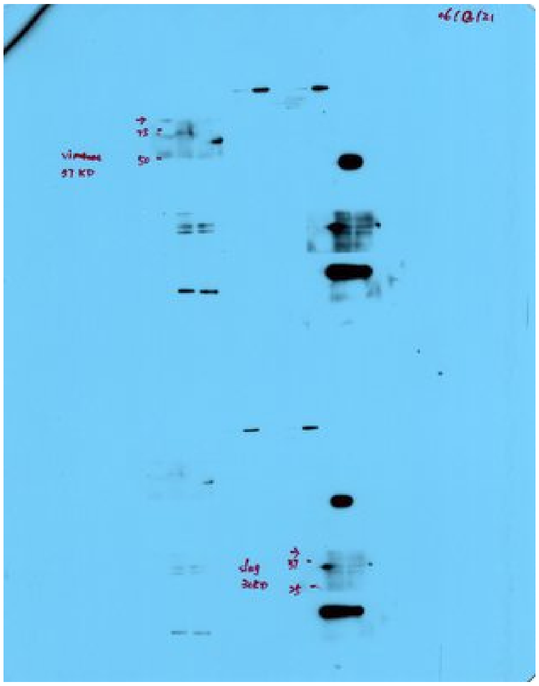

img06082021_103.jpg(527.5 KB) - download

James Drury (jmdr236@uky.edu) - Jul 12, 2021, 12:13 PM EDT

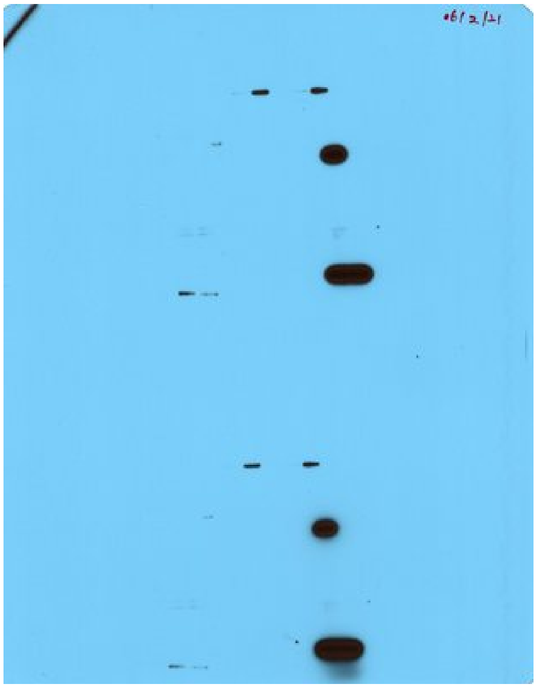

img06082021_104.jpg(511.7 KB) - download

James Drury (jmdr236@uky.edu) - Jul 12, 2021, 12:13 PM EDT

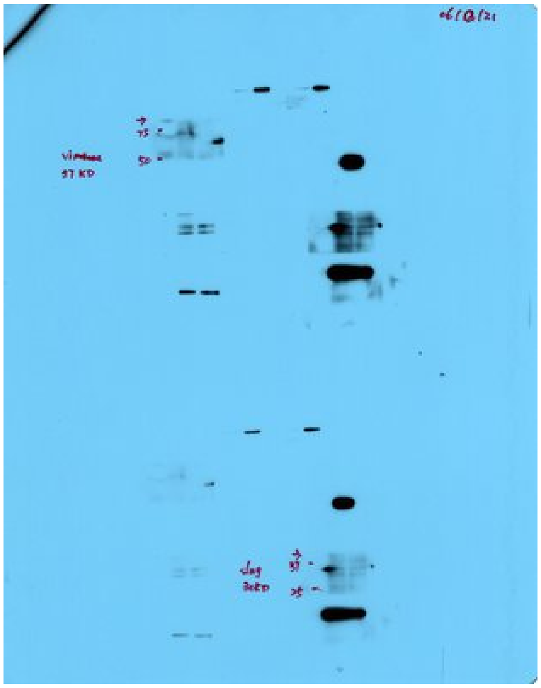

img06082021_105.jpg(529.7 KB) - download

James Drury (jmdr236@uky.edu) - Jul 12, 2021, 12:14 PM EDT

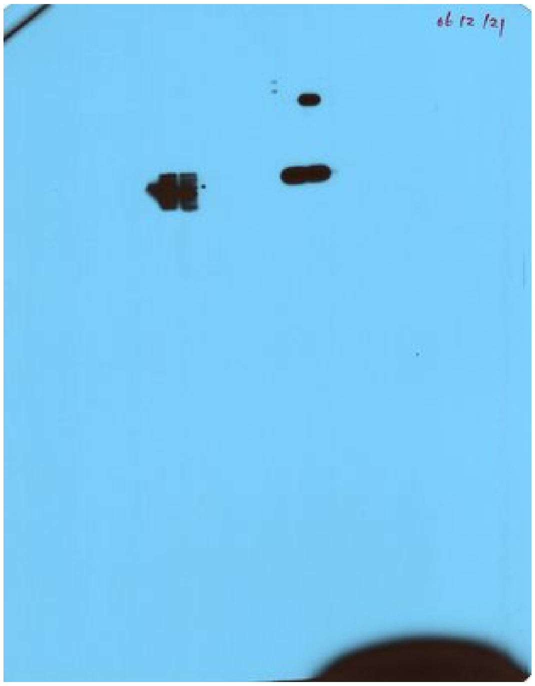

img06082021_106.jpg(455.9 KB) - download

James Drury (jmdr236@uky.edu) - Jul 12, 2021, 12:14 PM EDT

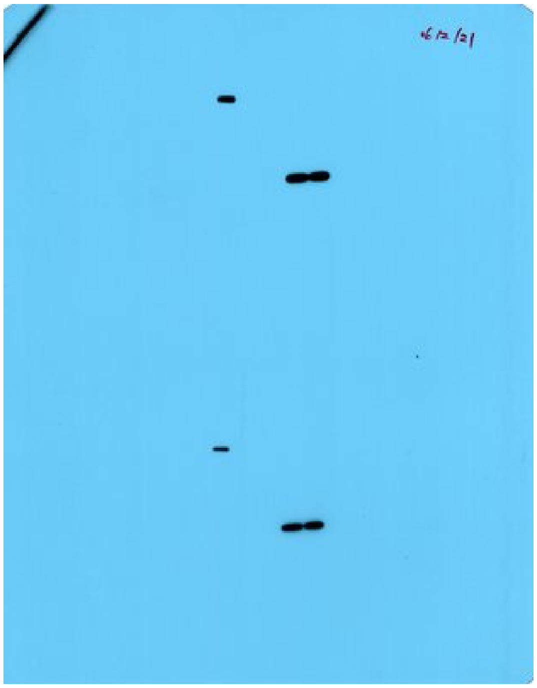

img06082021_107.jpg(520.3 KB) - download

James Drury (jmdr236@uky.edu) - Jul 12, 2021, 12:14 PM EDT

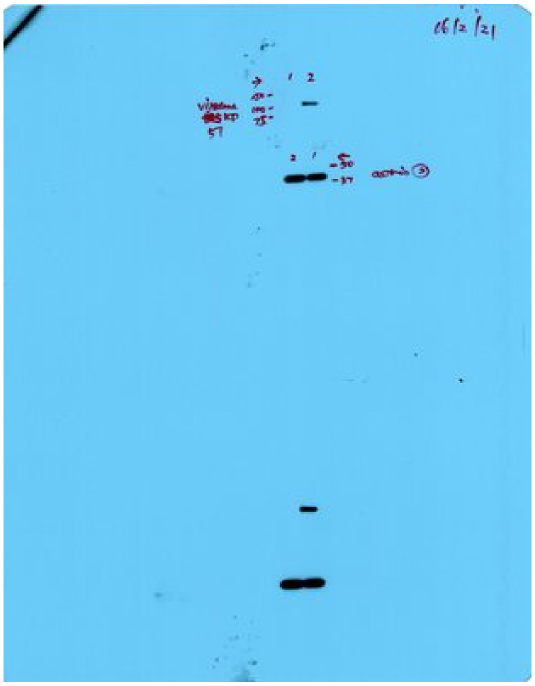

img06082021_108.jpg(551.2 KB) - download

James Drury (jmdr236@uky.edu) - Jul 12, 2021, 12:14 PM EDT

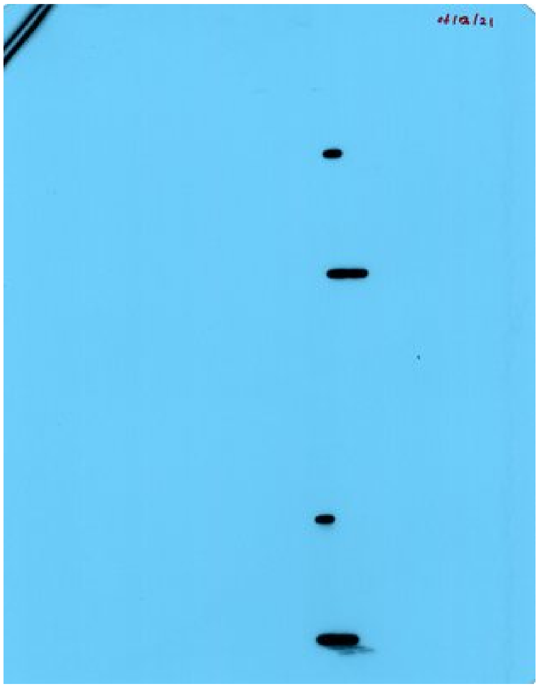

img06082021_109.jpg(564.4 KB) - download

HCT116 siMMP28 e-cad cleaved 6-8-21

James Drury (jmdr236@uky.edu) - Jul 12, 2021, 12:14 PM EDT

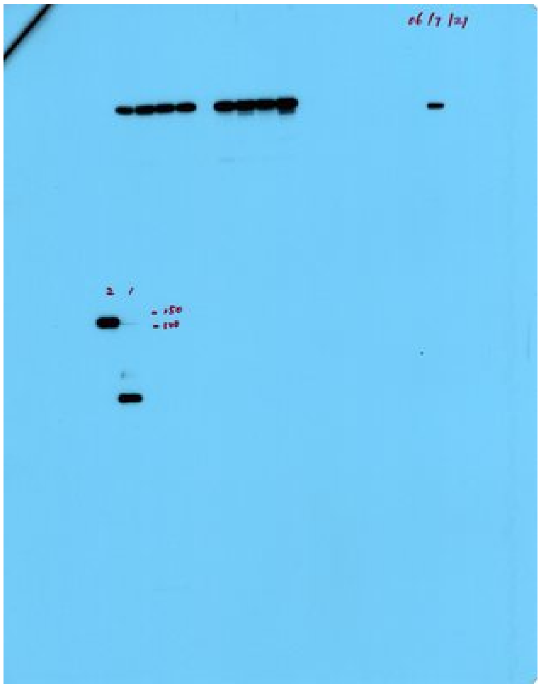

img06082021_085.jpg(501.5 KB) - download

James Drury (jmdr236@uky.edu) - Jul 12, 2021, 12:14 PM EDT

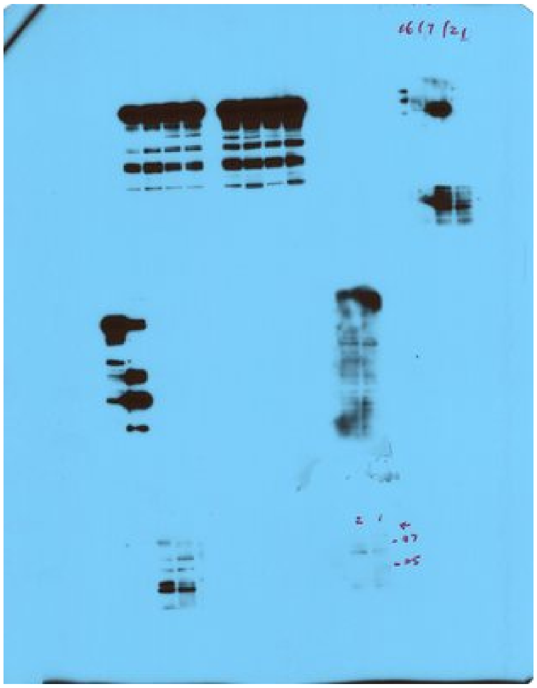

img06082021_086.jpg(520.2 KB) - download

James Drury (jmdr236@uky.edu) - Jul 12, 2021, 12:14 PM EDT

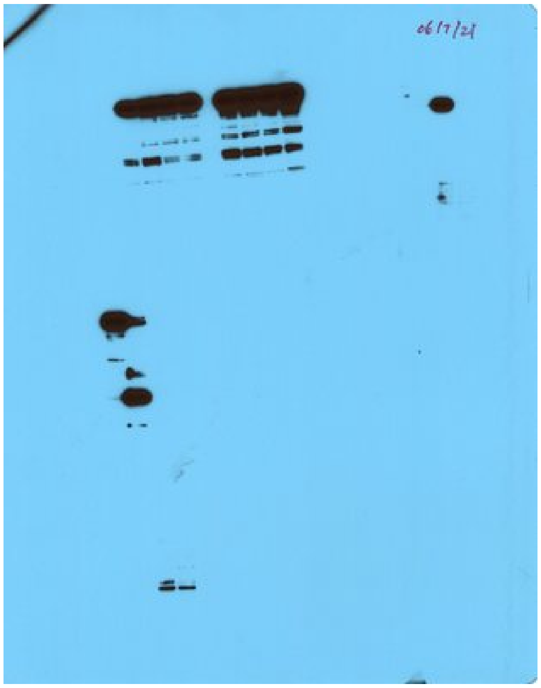

img06082021_087.jpg(640.9 KB) - download

James Drury (jmdr236@uky.edu) - Jul 12, 2021, 12:14 PM EDT

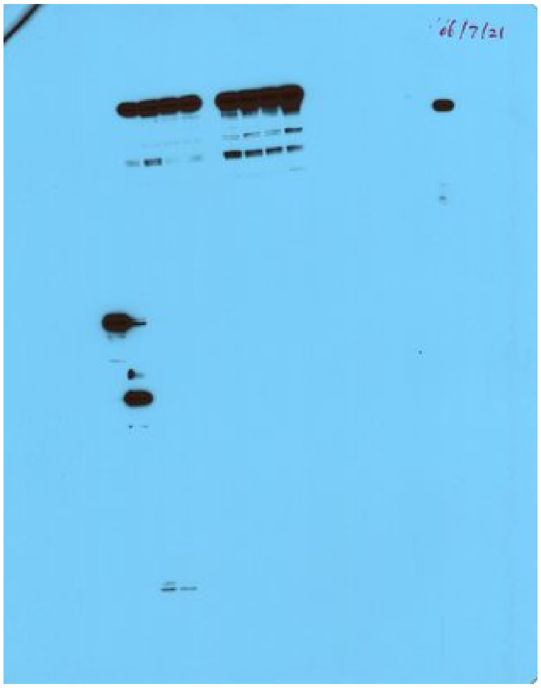

img06082021_088.jpg(498 KB) - download

James Drury (jmdr236@uky.edu) - Jul 12, 2021, 12:14 PM EDT

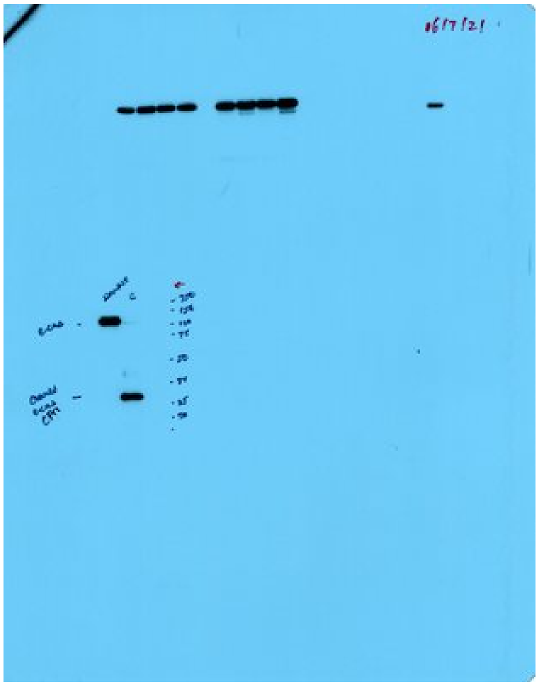

img06082021_089.jpg(533.2 KB) - download

James Drury (jmdr236@uky.edu) - Jul 12, 2021, 12:14 PM EDT

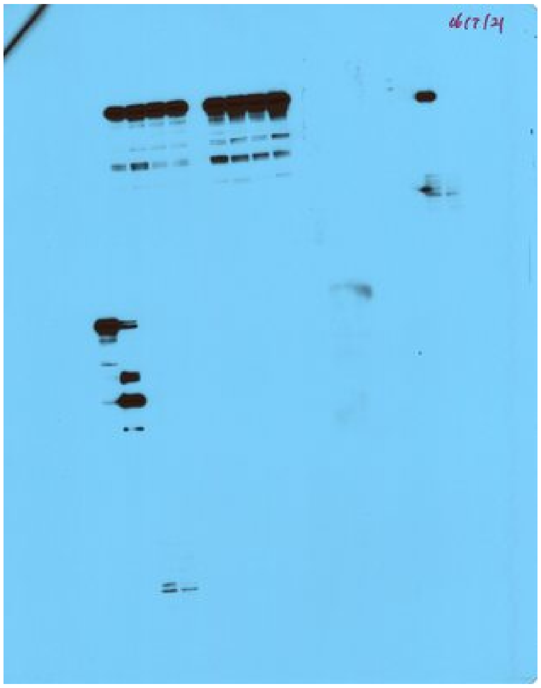

img06082021_090.jpg(482 KB) - download

James Drury (jmdr236@uky.edu) - Jul 12, 2021, 12:14 PM EDT

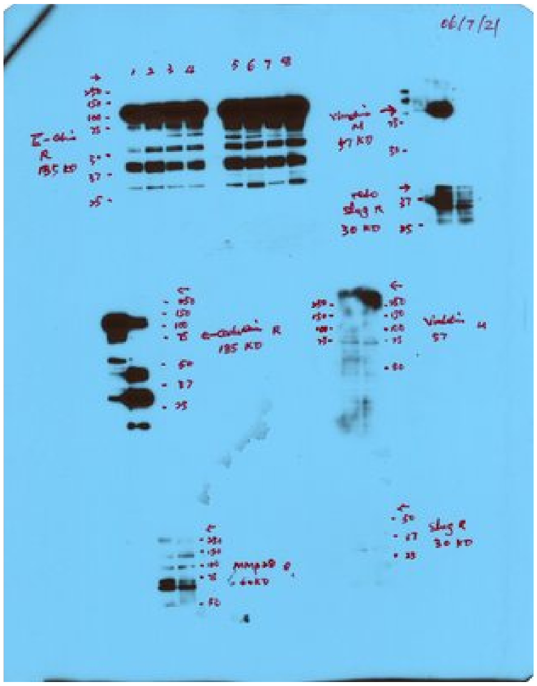

img06082021_091.jpg(592.1 KB) - download

James Drury (jmdr236@uky.edu) - Jul 12, 2021, 12:14 PM EDT

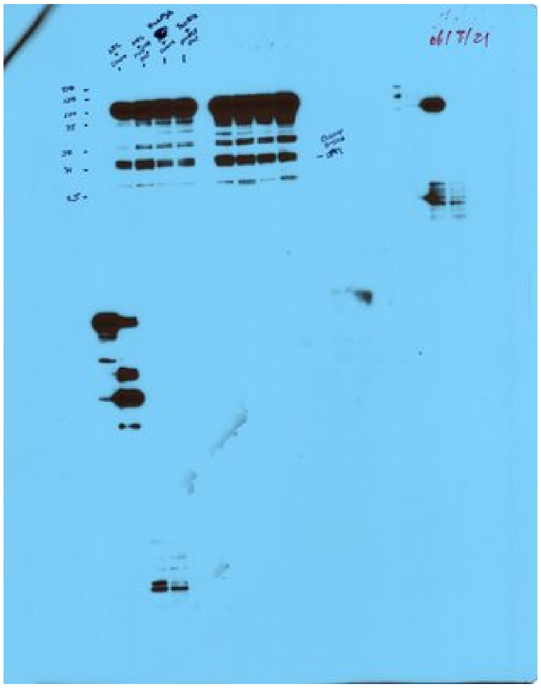

img06082021_092.jpg(514.4 KB) - download

James Drury (jmdr236@uky.edu) - Jul 12, 2021, 12:14 PM EDT

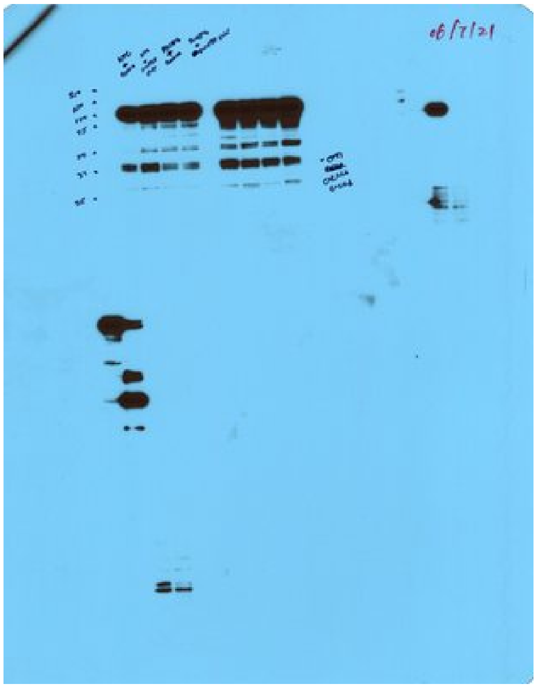

img06082021_093.jpg(516.1 KB) - download

James Drury (jmdr236@uky.edu) - Jul 12, 2021, 12:15 PM EDT

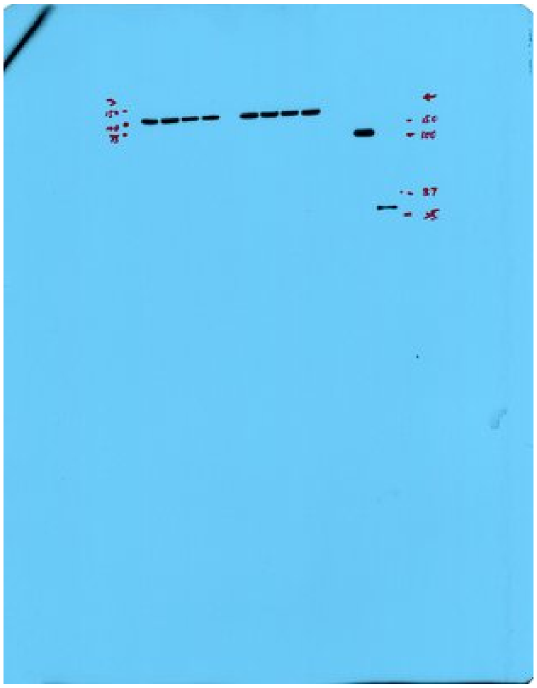

img06082021_094.jpg(528 KB) - download

James Drury (jmdr236@uky.edu) - Jul 12, 2021, 12:15 PM EDT

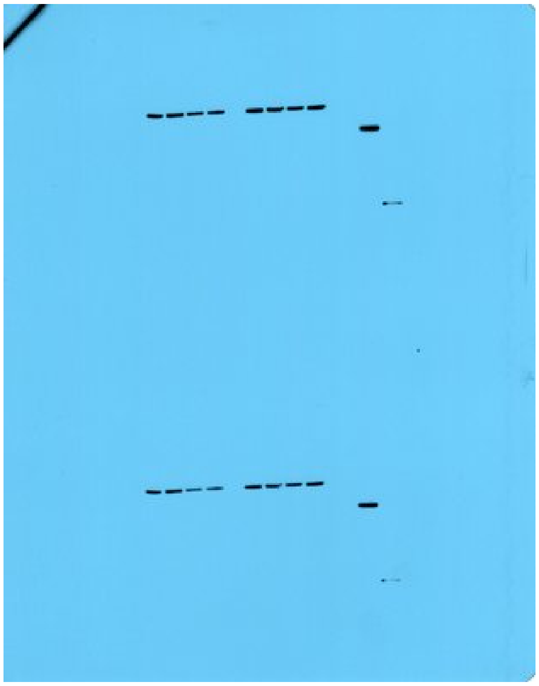

img06082021_095.jpg(663 KB) - download

James Drury (jmdr236@uky.edu) - Jul 12, 2021, 12:15 PM EDT

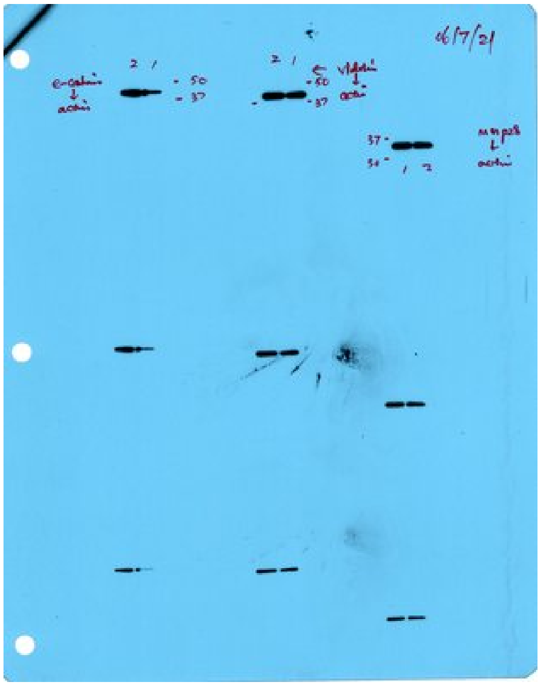

img06102021_110.jpg(614 KB) - download

James Drury (jmdr236@uky.edu) - Jul 12, 2021, 12:15 PM EDT

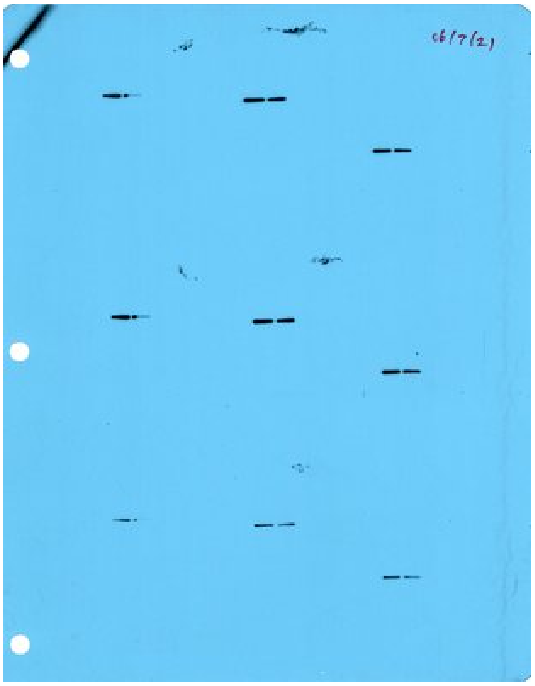

img06212021_130_actin.jpg(584.9 KB) - download

HT29 LuM0 LuM3 4-22-21

James Drury (jmdr236@uky.edu) - Jul 12, 2021, 12:15 PM EDT

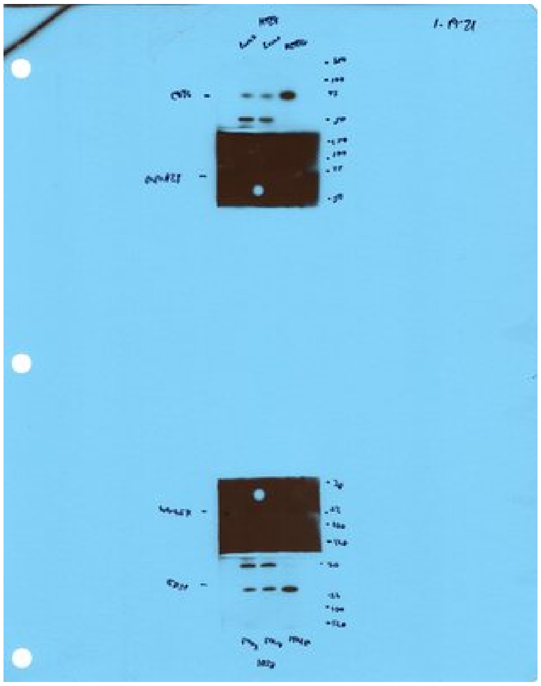

img04232021_046.jpg(619.6 KB) - download

James Drury (jmdr236@uky.edu) - Jul 12, 2021, 12:15 PM EDT

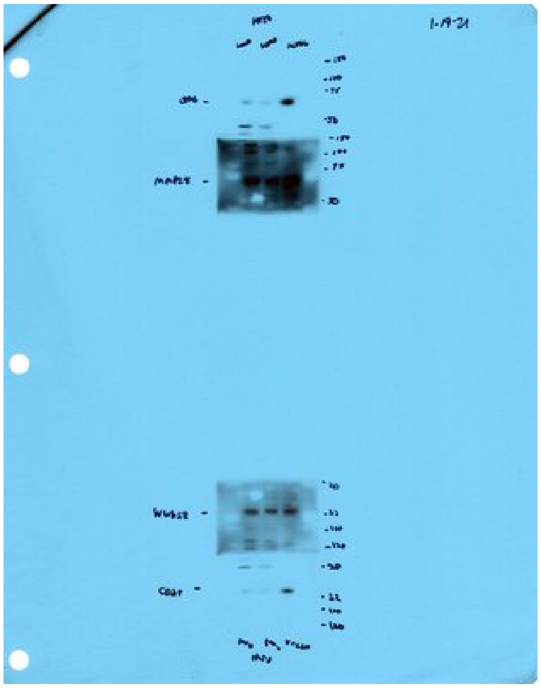

img04232021_047.jpg(831.6 KB) - download

James Drury (jmdr236@uky.edu) - Jul 12, 2021, 12:15 PM EDT

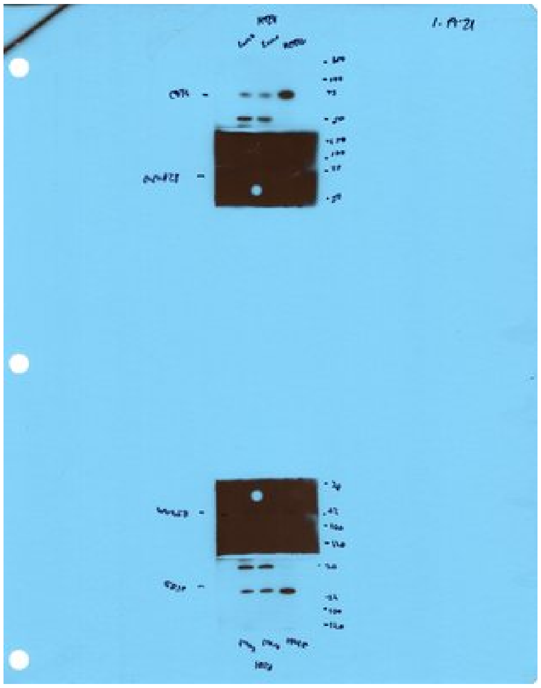

img04232021_048.jpg(791.4 KB) - download

James Drury (jmdr236@uky.edu) - Jul 12, 2021, 12:15 PM EDT

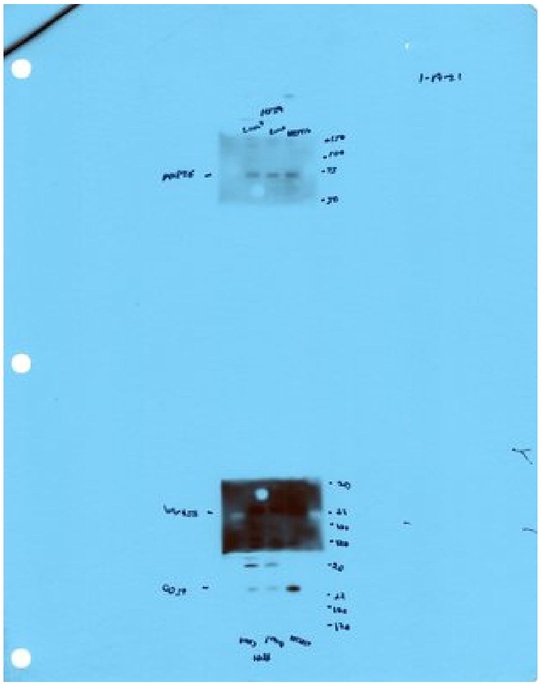

img04232021_049.jpg(618.9 KB) - download

James Drury (jmdr236@uky.edu) - Jul 12, 2021, 12:15 PM EDT

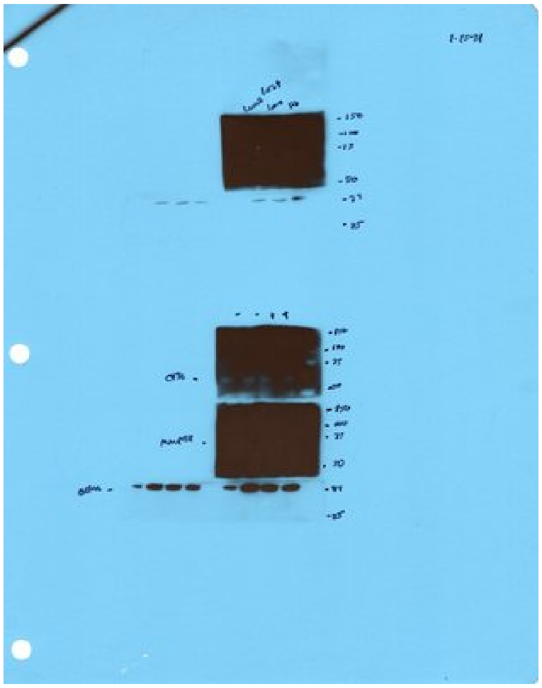

img04232021_050.jpg(632 KB) - download

LuM0 LuM3 3-1-21

James Drury (jmdr236@uky.edu) - Jul 12, 2021, 12:17 PM EDT

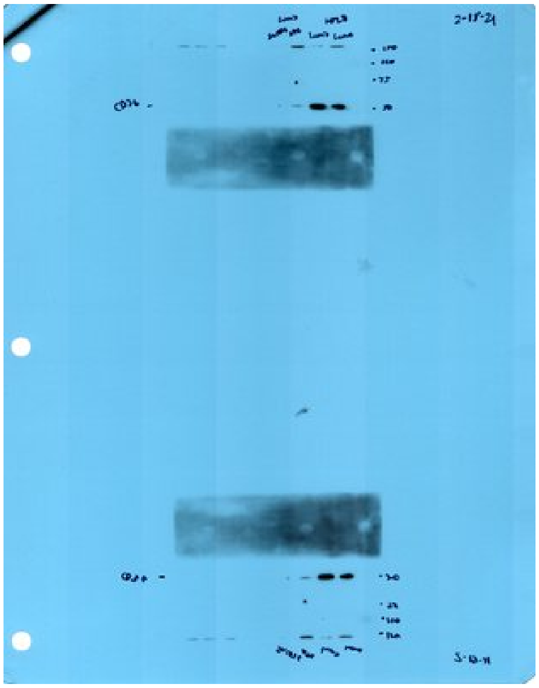

img03012021_023.jpg(596.3 KB) - download

James Drury (jmdr236@uky.edu) - Jul 12, 2021, 12:17 PM EDT

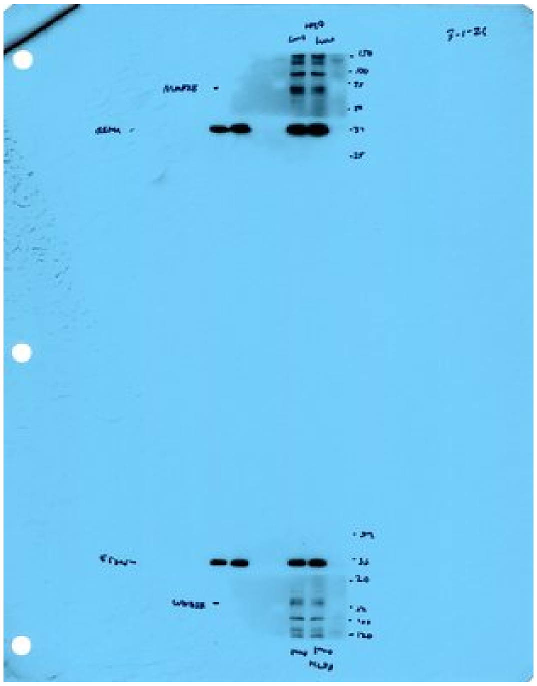

img03012021_024.jpg(587.8 KB) - download

James Drury (jmdr236@uky.edu) - Jul 12, 2021, 12:17 PM EDT

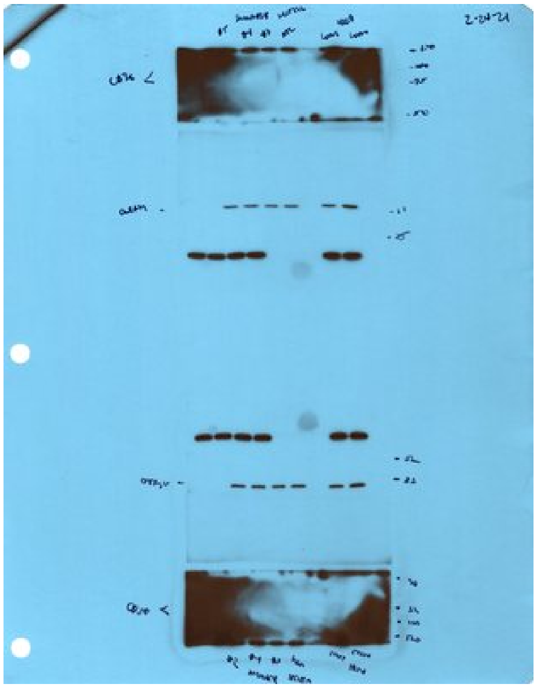

img03012021_025.jpg(698.4 KB) - download

LuM3 shCD36 5-4-21

James Drury (jmdr236@uky.edu) - Jul 12, 2021, 12:17 PM EDT

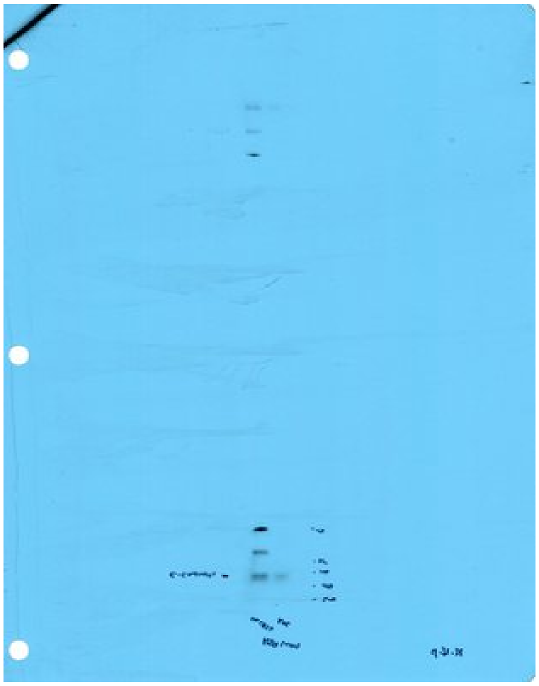

img05042021_051.jpg(732.7 KB) - download

James Drury (jmdr236@uky.edu) - Jul 12, 2021, 12:17 PM EDT

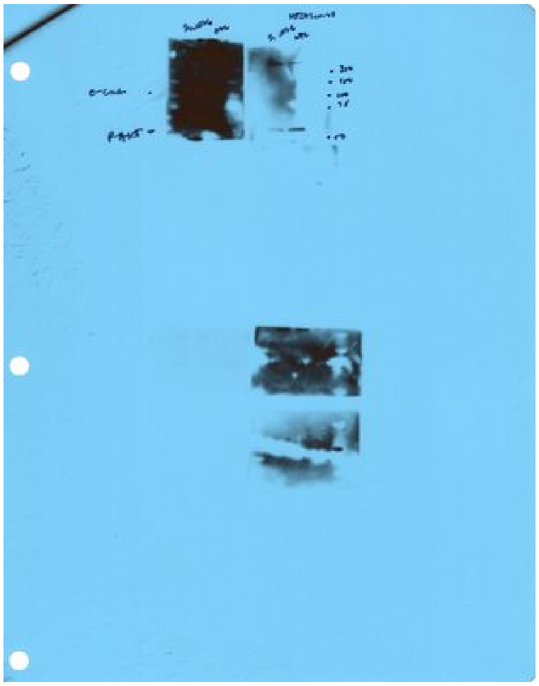

img05042021_052.jpg(695.1 KB) - download

James Drury (jmdr236@uky.edu) - Jul 12, 2021, 12:17 PM EDT

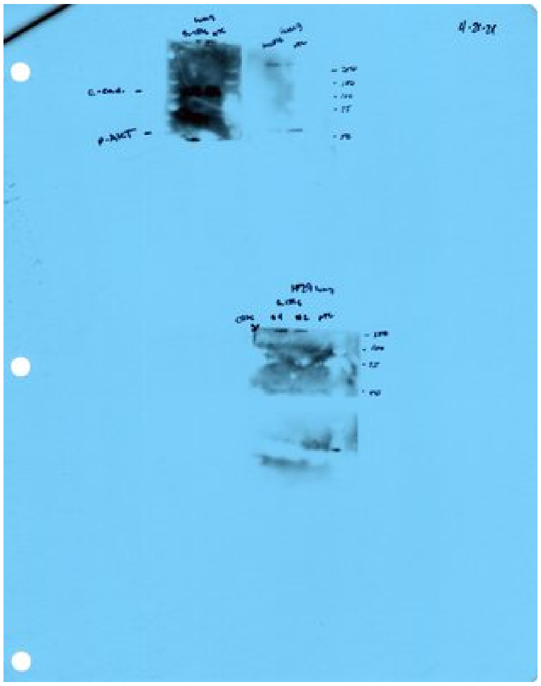

img05042021_053.jpg(572.4 KB) - download

James Drury (jmdr236@uky.edu) - Jul 12, 2021, 12:17 PM EDT

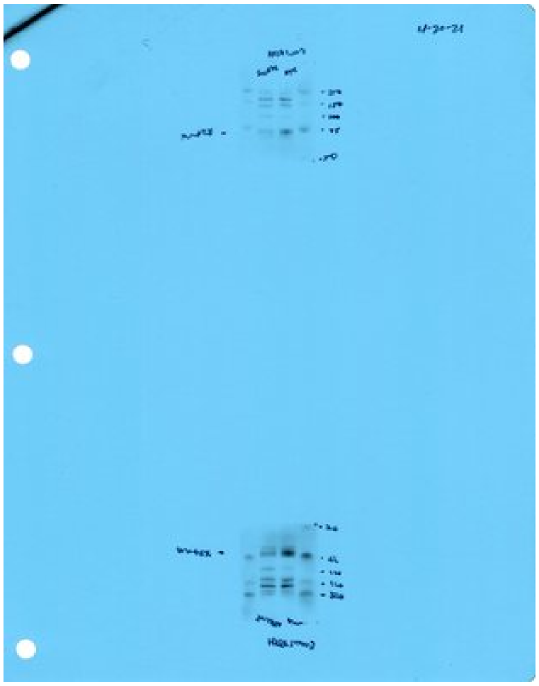

img05042021_054.jpg(727.3 KB) - download

James Drury (jmdr236@uky.edu) - Jul 12, 2021, 12:17 PM EDT

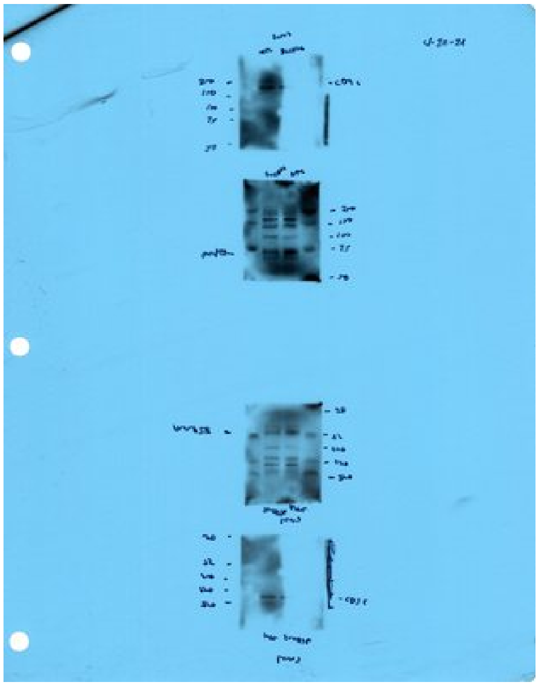

img05042021_055.jpg(731.7 KB) - download

James Drury (jmdr236@uky.edu) - Jul 12, 2021, 12:17 PM EDT

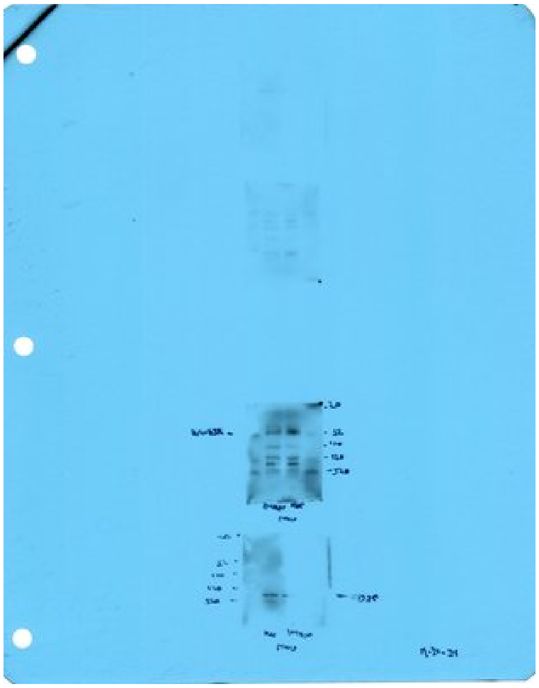

img05042021_056.jpg(569.6 KB) - download

James Drury (jmdr236@uky.edu) - Jul 12, 2021, 12:17 PM EDT

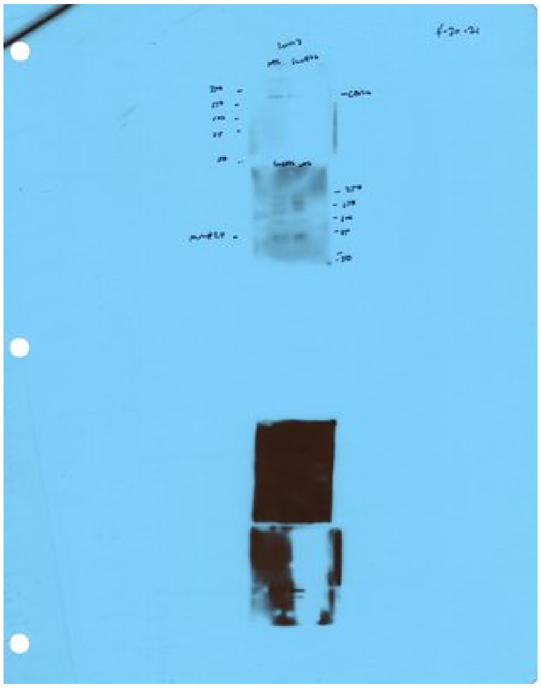

img05042021_057.jpg(684.9 KB) - download

James Drury (jmdr236@uky.edu) - Jul 12, 2021, 12:17 PM EDT

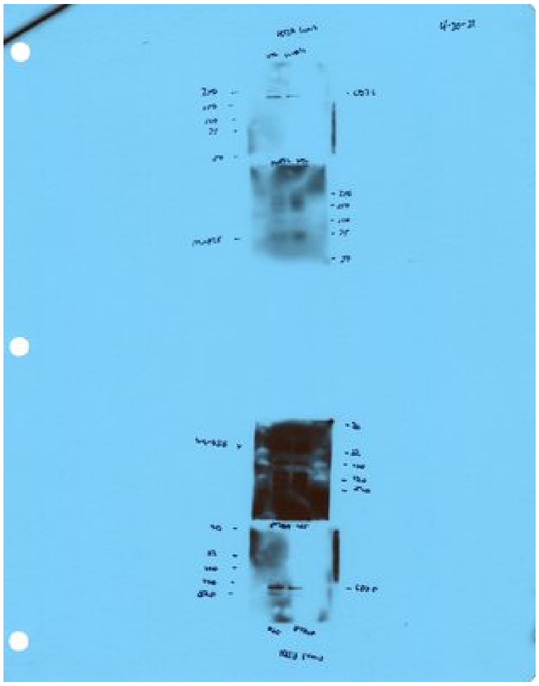

img05042021_058.jpg(693.7 KB) - download

James Drury (jmdr236@uky.edu) - Jul 12, 2021, 12:17 PM EDT

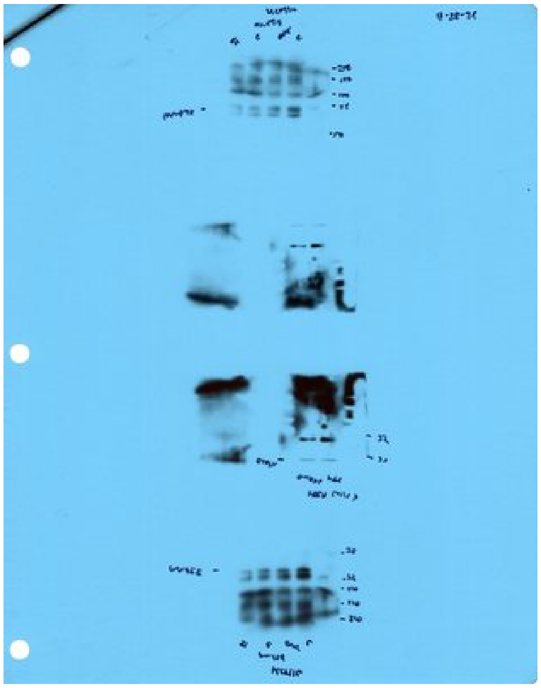

img05042021_059.jpg(729.9 KB) - download

James Drury (jmdr236@uky.edu) - Jul 12, 2021, 12:17 PM EDT

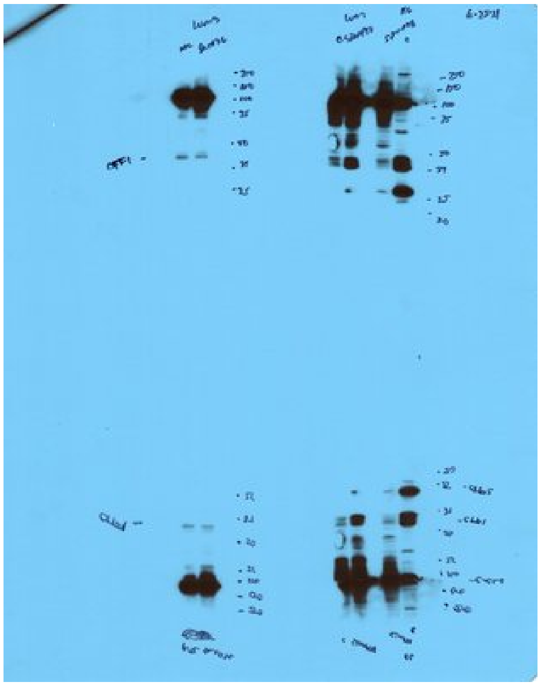

img06272021_132.jpg(673.2 KB) - download

Pt 2449 Matched Tissues 6-15-21

James Drury (jmdr236@uky.edu) - Jul 12, 2021, 12:17 PM EDT

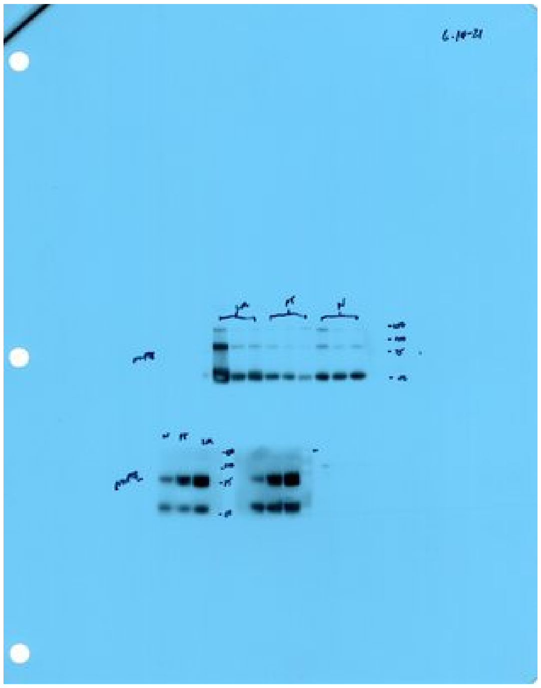

img06152021_123.jpg(474.8 KB) - download

James Drury (jmdr236@uky.edu) - Jul 12, 2021, 12:17 PM EDT

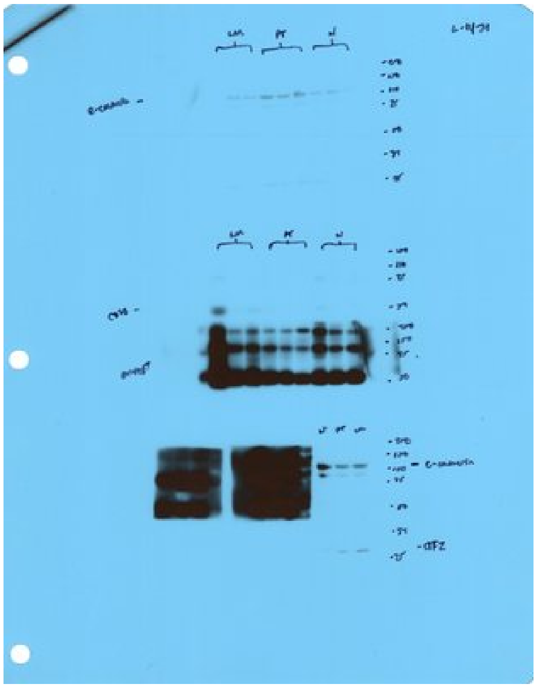

img06152021_124.jpg(474 KB) - download

James Drury (jmdr236@uky.edu) - Jul 12, 2021, 12:18 PM EDT

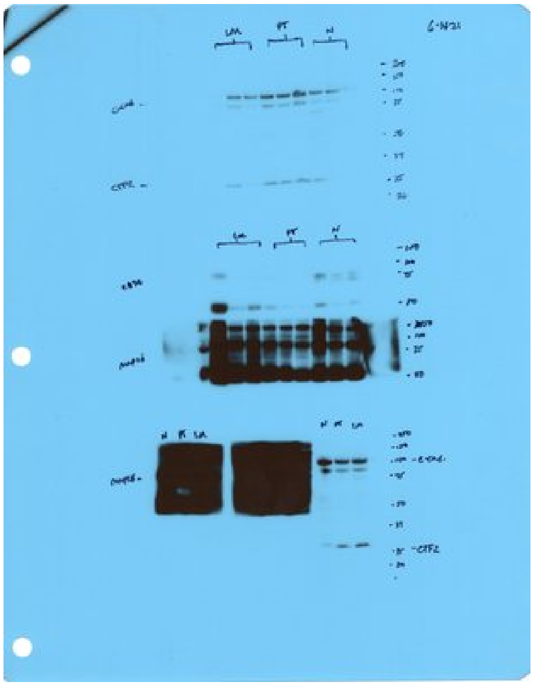

img06152021_125.jpg(484.7 KB) - download

James Drury (jmdr236@uky.edu) - Jul 12, 2021, 12:18 PM EDT

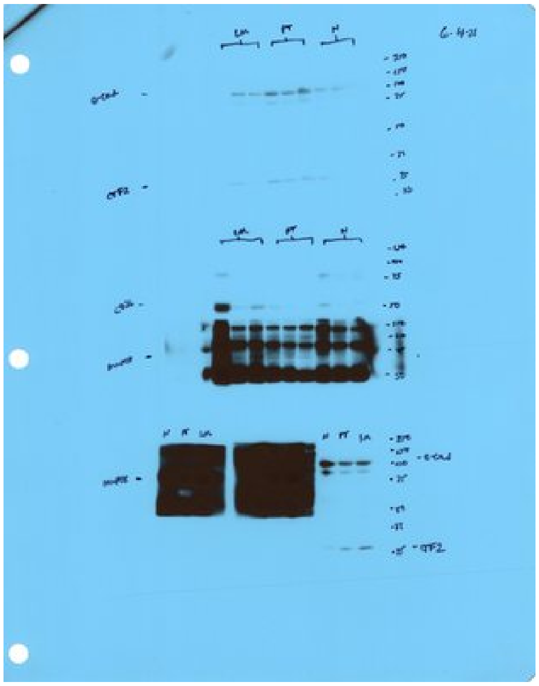

img06152021_126.jpg(591.9 KB) - download

James Drury (jmdr236@uky.edu) - Jul 12, 2021, 12:18 PM EDT

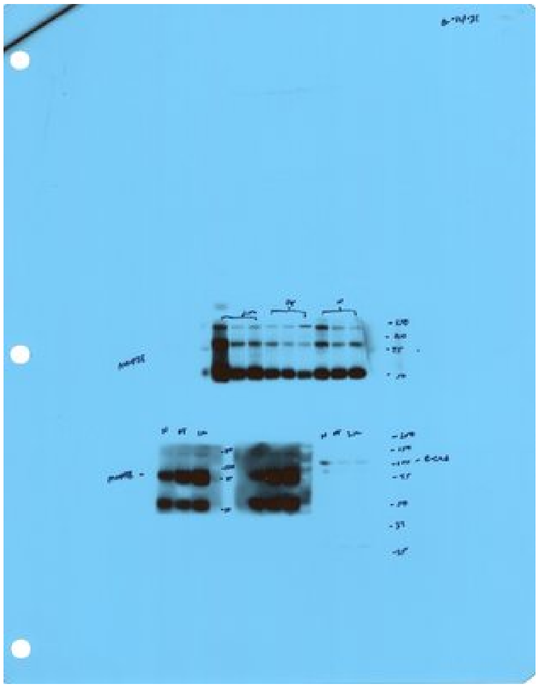

img06152021_127.jpg(438.4 KB) - download

James Drury (jmdr236@uky.edu) - Jul 12, 2021, 12:18 PM EDT

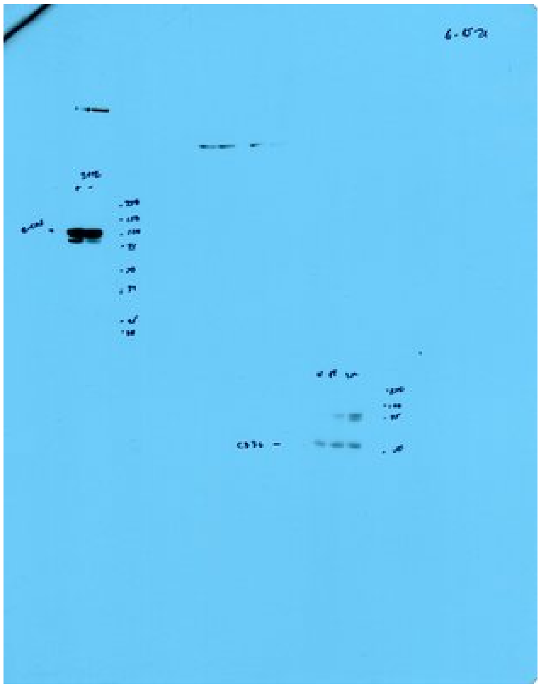

img06152021_128.jpg(516.5 KB) - download

James Drury (jmdr236@uky.edu) - Jul 12, 2021, 12:18 PM EDT

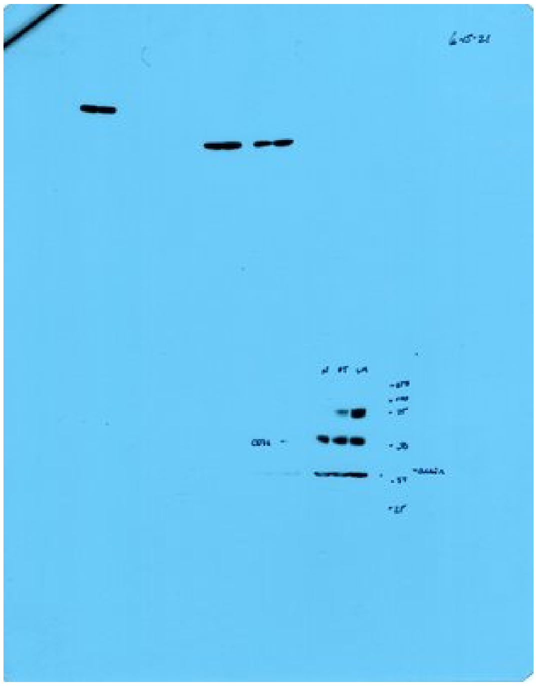

img06152021_129.jpg(496.8 KB) - download

Pt 2377 Matched Tissues 6-27-21

James Drury (jmdr236@uky.edu) - Jul 12, 2021, 12:18 PM EDT

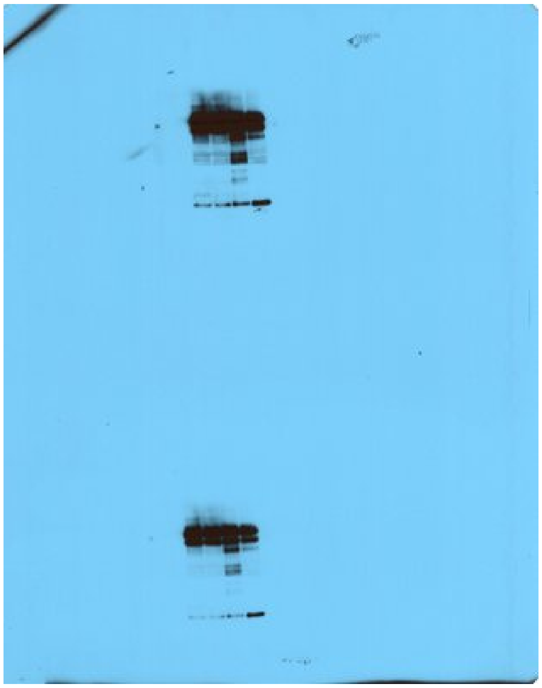

img06272021_134.jpg(501.8 KB) - download

James Drury (jmdr236@uky.edu) - Jul 12, 2021, 12:18 PM EDT

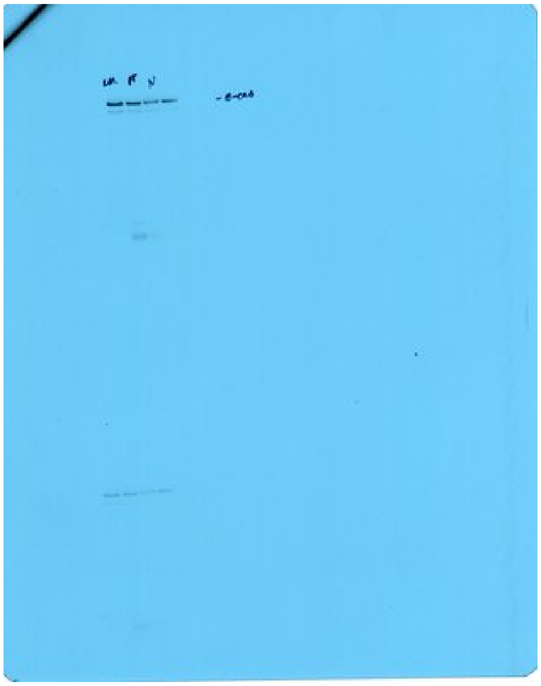

img06272021_135.jpg(524 KB) - download

James Drury (jmdr236@uky.edu) - Jul 12, 2021, 12:18 PM EDT

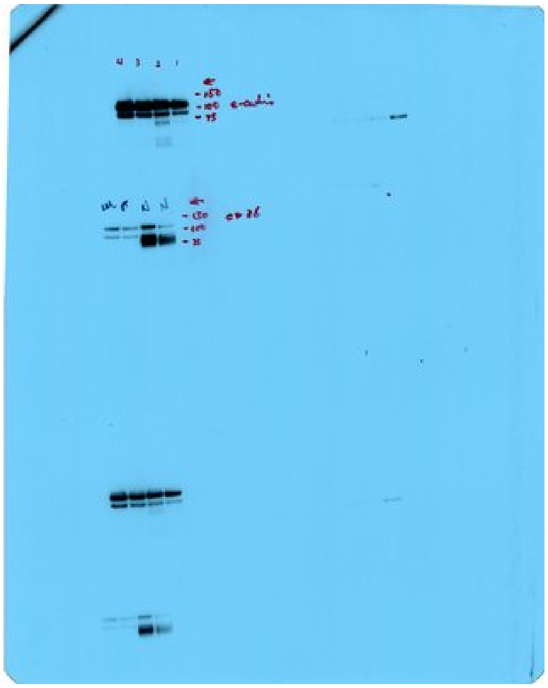

img06272021_136.jpg(541.4 KB) - download

James Drury (jmdr236@uky.edu) - Jul 12, 2021, 12:18 PM EDT

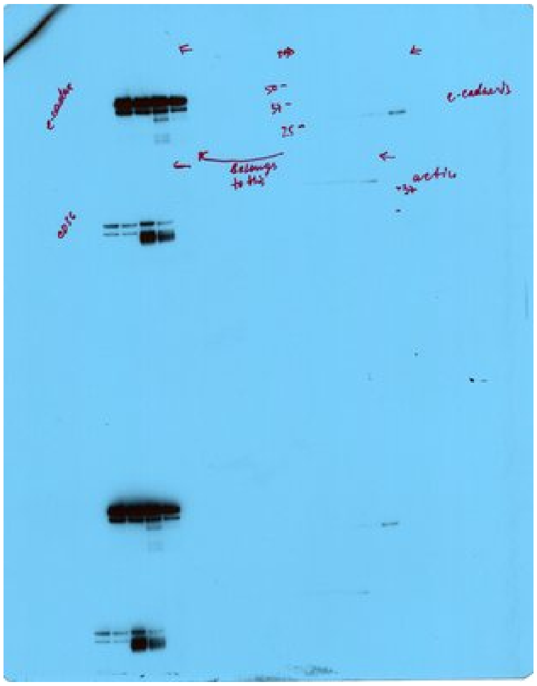

img06272021_137.jpg(515.2 KB) - download

James Drury (jmdr236@uky.edu) - Jul 12, 2021, 12:18 PM EDT

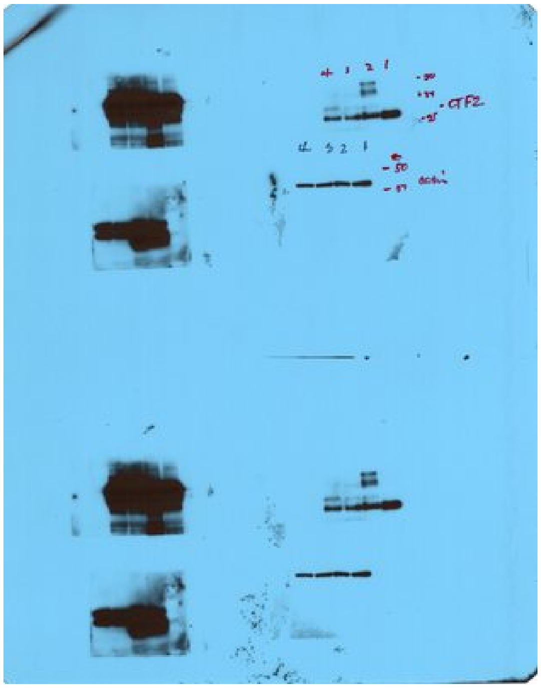

img06272021_138.jpg(517.9 KB) - download

James Drury (jmdr236@uky.edu) - Jul 12, 2021, 12:18 PM EDT

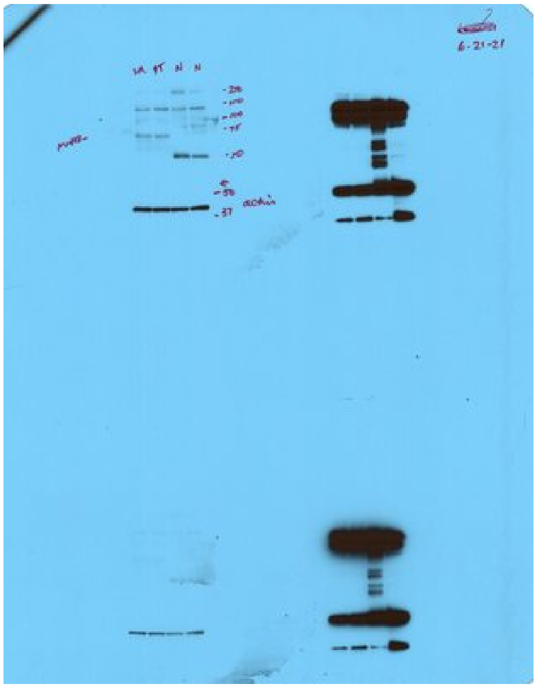

img06272021_139.jpg(546.5 KB) - download

James Drury (jmdr236@uky.edu) - Jul 12, 2021, 12:18 PM EDT

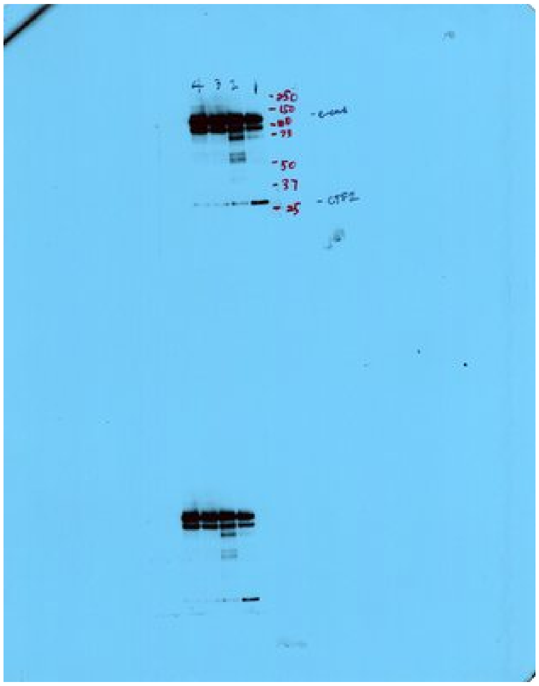

img06272021_140.jpg(531.8 KB) - download

James Drury (jmdr236@uky.edu) - Jul 12, 2021, 12:18 PM EDT

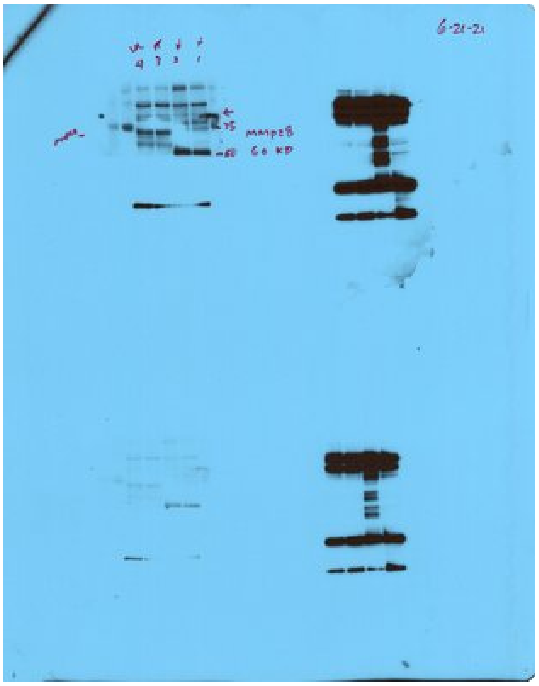

img06272021_141.jpg(527.6 KB) - download

James Drury (jmdr236@uky.edu) - Jul 12, 2021, 12:18 PM EDT

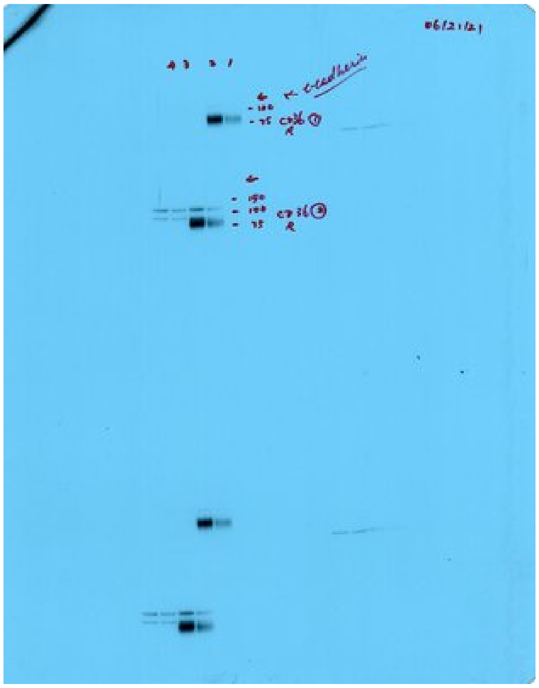

img06272021_142.jpg(569.2 KB) - download

James Drury (jmdr236@uky.edu) - Jul 12, 2021, 12:18 PM EDT

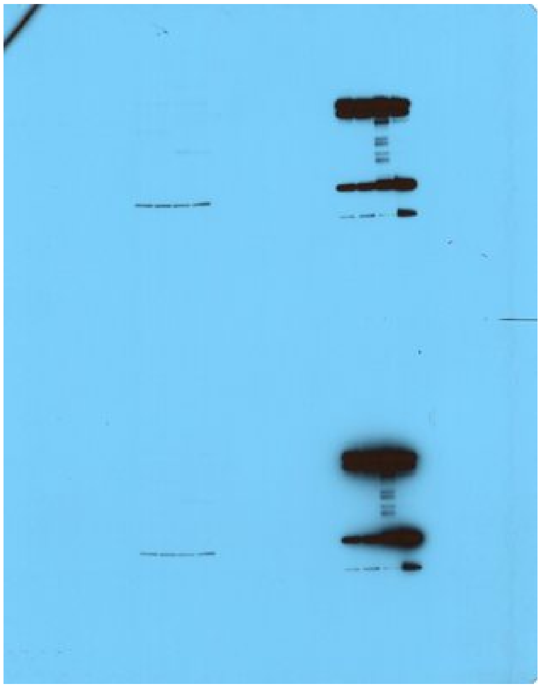

img06272021_143.jpg(661.3 KB) - download

James Drury (jmdr236@uky.edu) - Jul 12, 2021, 12:18 PM EDT

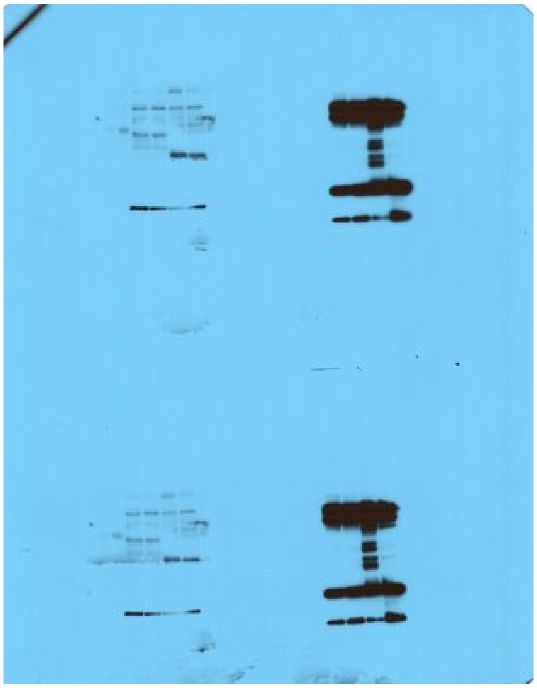

img06272021_144.jpg(528.4 KB) - download

James Drury (jmdr236@uky.edu) - Jul 12, 2021, 12:18 PM EDT

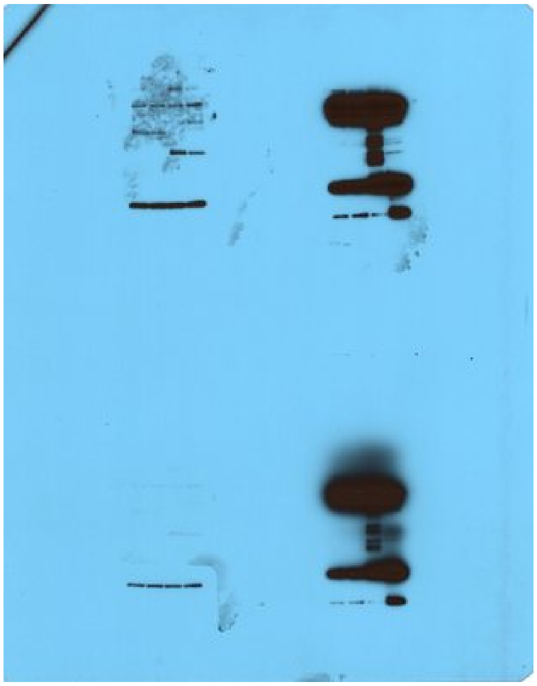

img06272021_145.jpg(508.5 KB) - download

James Drury (jmdr236@uky.edu) - Jul 12, 2021, 12:18 PM EDT

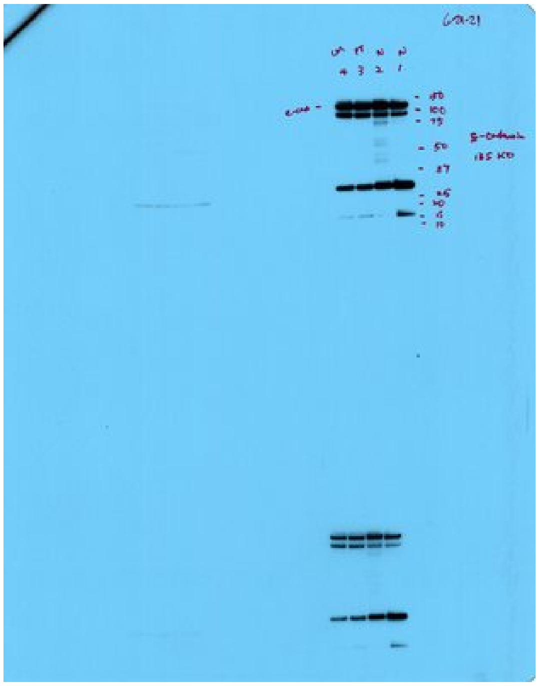

img06272021_146.jpg(706 KB) - download

James Drury (jmdr236@uky.edu) - Jul 12, 2021, 12:18 PM EDT

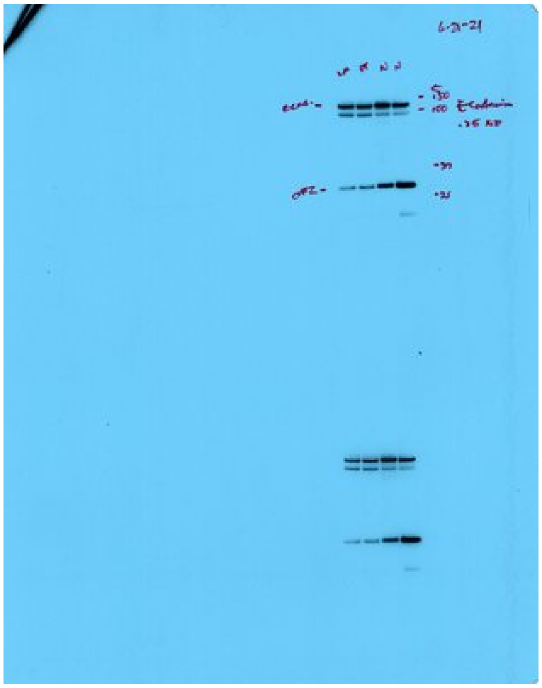

img06272021_147.jpg(583.7 KB) - download

James Drury (jmdr236@uky.edu) - Jul 12, 2021, 12:18 PM EDT

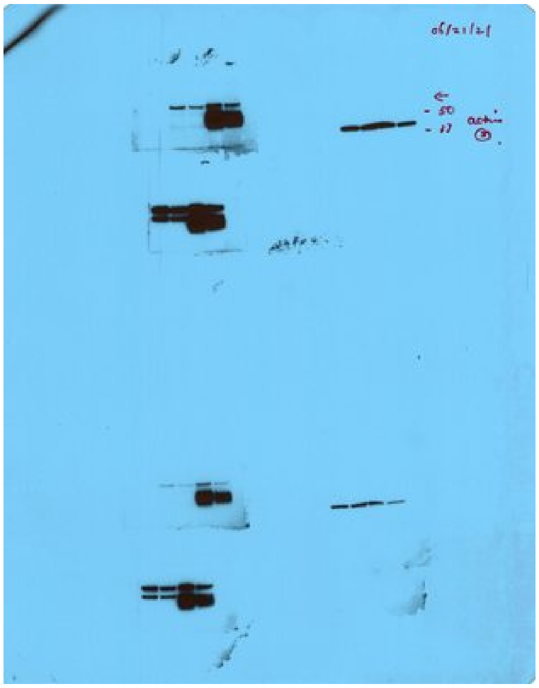

img06272021_148.jpg(537.1 KB) - download

James Drury (jmdr236@uky.edu) - Jul 12, 2021, 12:18 PM EDT

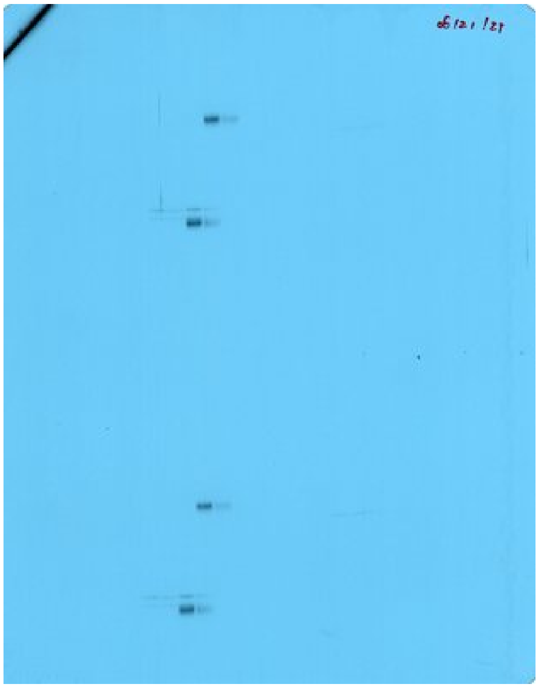

img06272021_149.jpg(548.1 KB) - download

James Drury (jmdr236@uky.edu) - Jul 12, 2021, 12:18 PM EDT

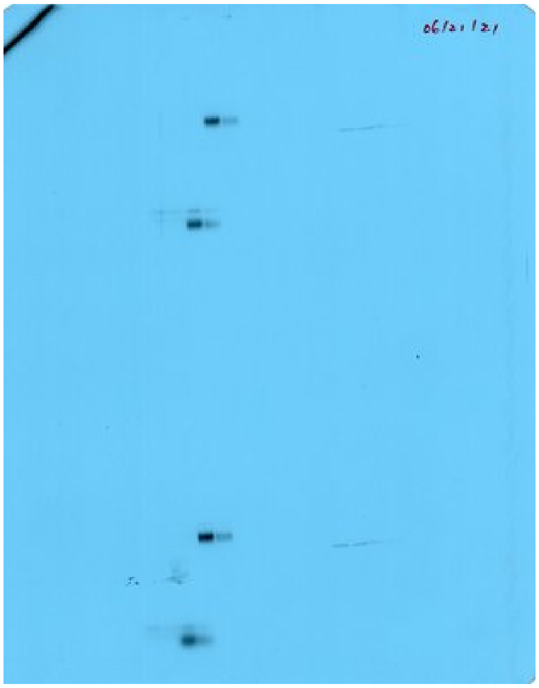

img06272021_150.jpg(545.6 KB) - download

James Drury (jmdr236@uky.edu) - Jul 12, 2021, 12:18 PM EDT

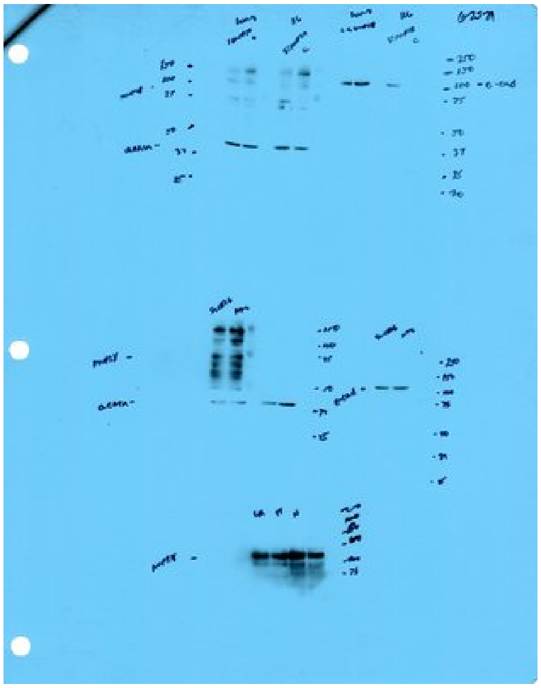

img06272021_131.jpg(554.3 KB) - download

James Drury (jmdr236@uky.edu) - Jul 12, 2021, 12:18 PM EDT

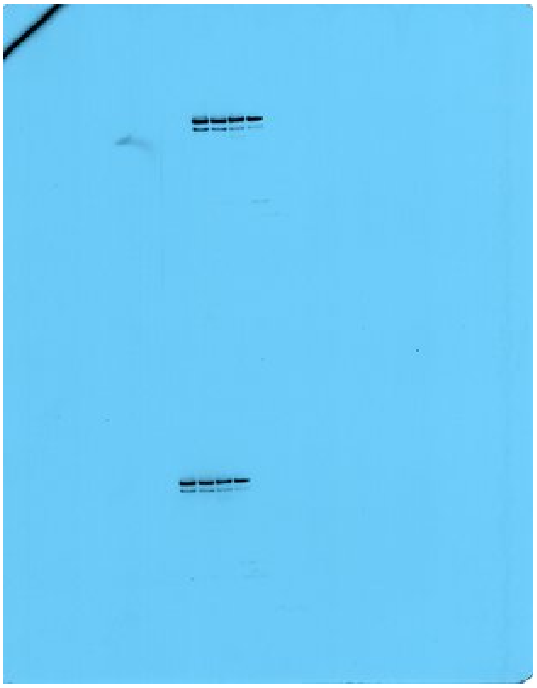

img06272021_133.jpg(519.3 KB) - download

Pt 2402 CD36 +/- 6-15-21

James Drury (jmdr236@uky.edu) - Jul 12, 2021, 12:20 PM EDT

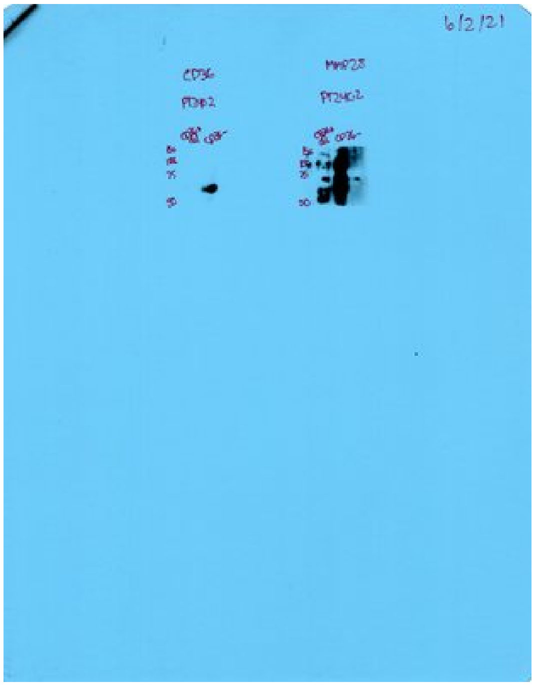

img06152021_117.jpg(558.4 KB) - download

James Drury (jmdr236@uky.edu) - Jul 12, 2021, 12:20 PM EDT

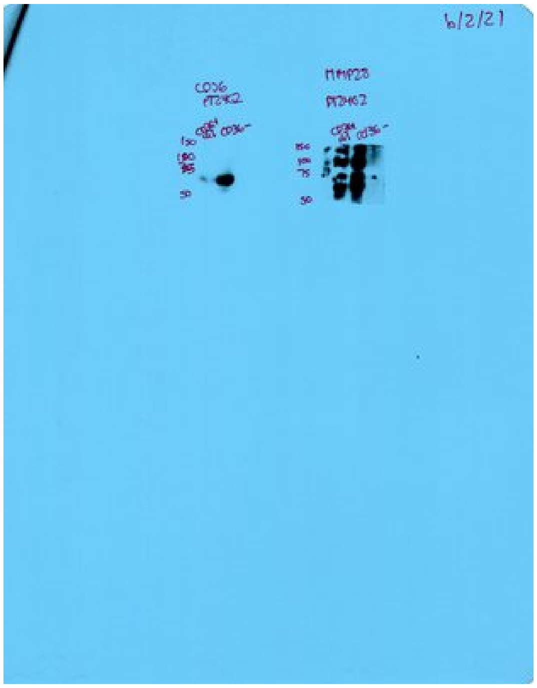

img06152021_118.jpg(581.5 KB) - download

James Drury (jmdr236@uky.edu) - Jul 12, 2021, 12:20 PM EDT

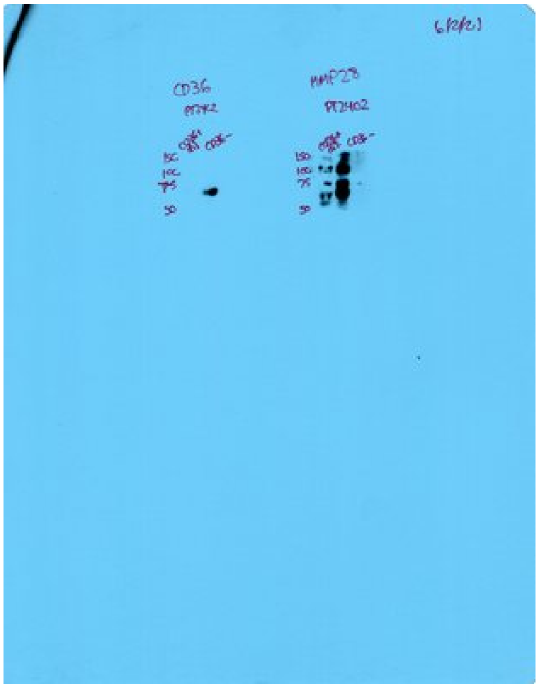

img06152021_119.jpg(589.7 KB) - download

James Drury (jmdr236@uky.edu) - Jul 12, 2021, 12:20 PM EDT

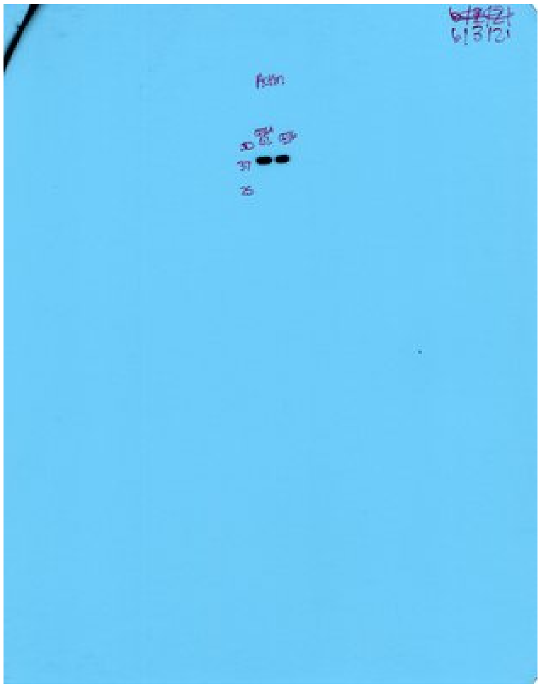

img06152021_120.jpg(727.1 KB) - download

James Drury (jmdr236@uky.edu) - Jul 12, 2021, 12:20 PM EDT

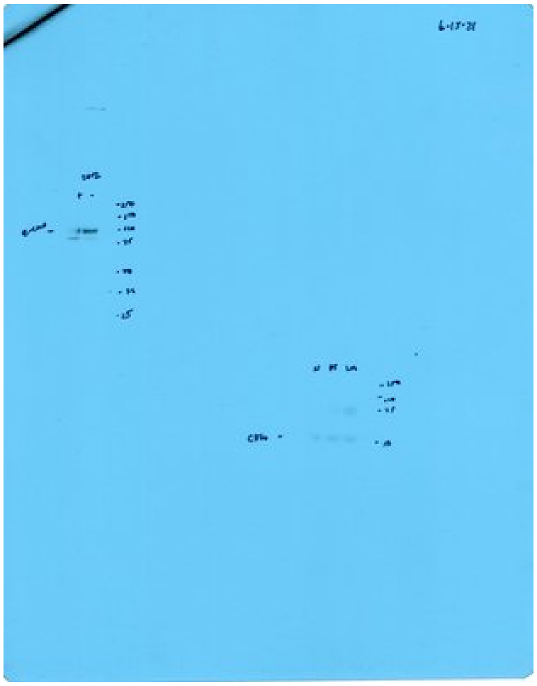

img06152021_121.jpg(497.1 KB) - download

James Drury (jmdr236@uky.edu) - Jul 12, 2021, 12:20 PM EDT

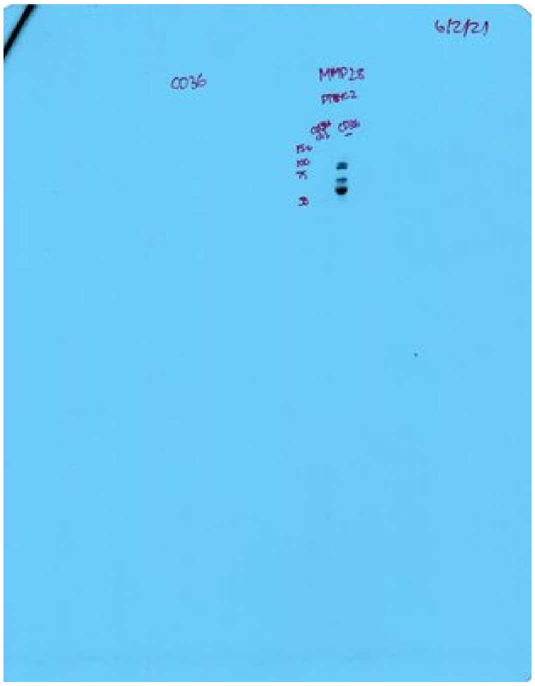

img06152021_122.jpg(569.3 KB) - download

Pt 2402 CD36 +/- 1-29-21

James Drury (jmdr236@uky.edu) - Jul 12, 2021, 12:20 PM EDT

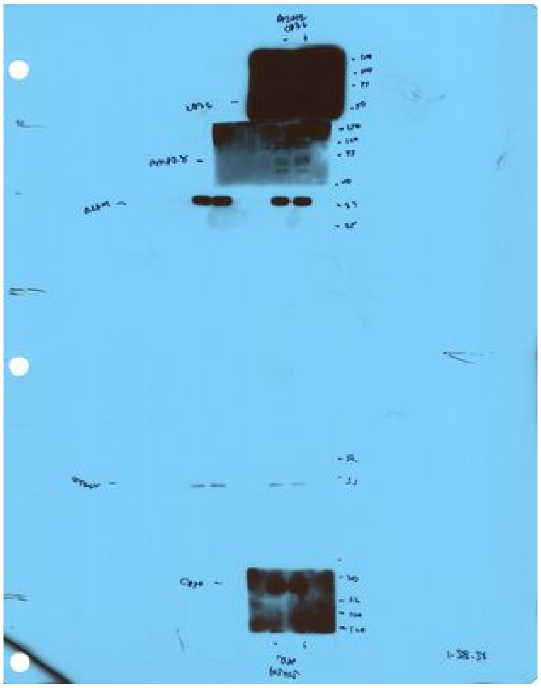

img01292021_010.jpg(622.7 KB) - download

James Drury (jmdr236@uky.edu) - Jul 12, 2021, 12:20 PM EDT

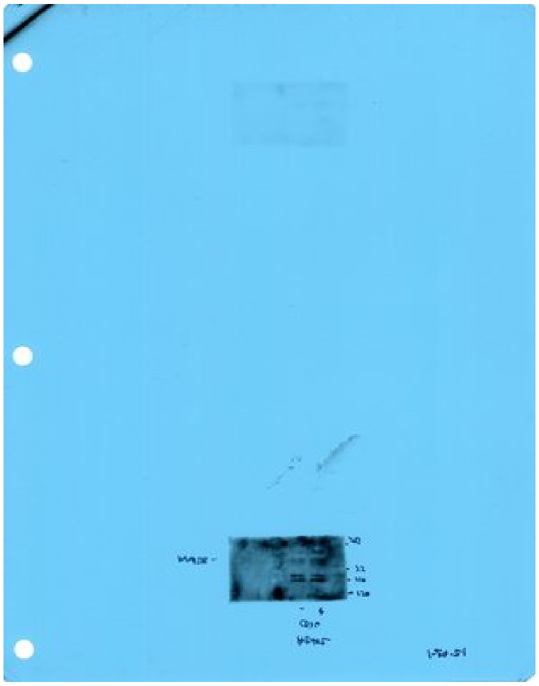

img01292021_011.jpg(489.4 KB) - download

James Drury (jmdr236@uky.edu) - Jul 12, 2021, 12:20 PM EDT

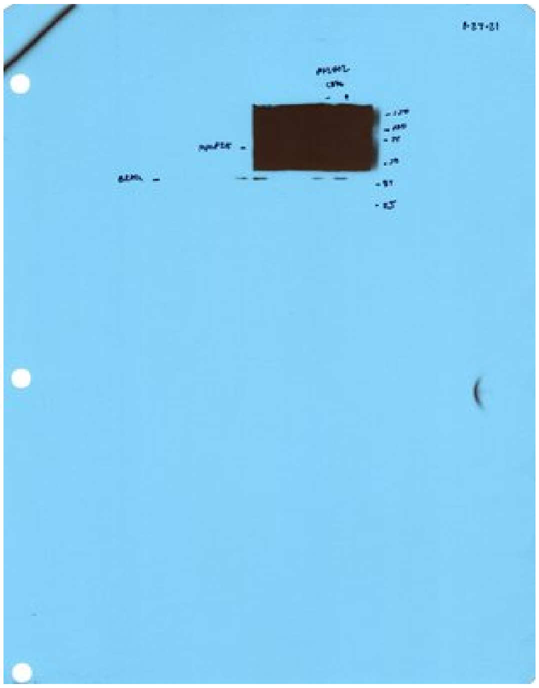

img01292021_012.jpg(614.1 KB) - download

James Drury (jmdr236@uky.edu) - Jul 12, 2021, 12:20 PM EDT

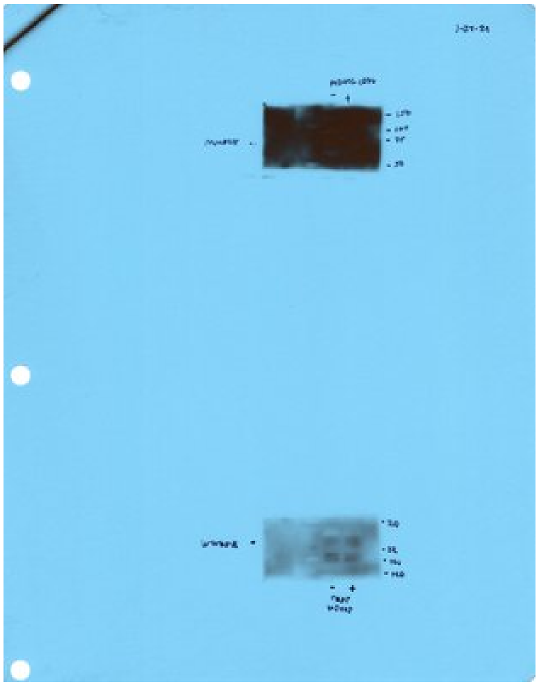

img01292021_013.jpg(587.1 KB) - download

James Drury (jmdr236@uky.edu) - Jul 12, 2021, 12:20 PM EDT

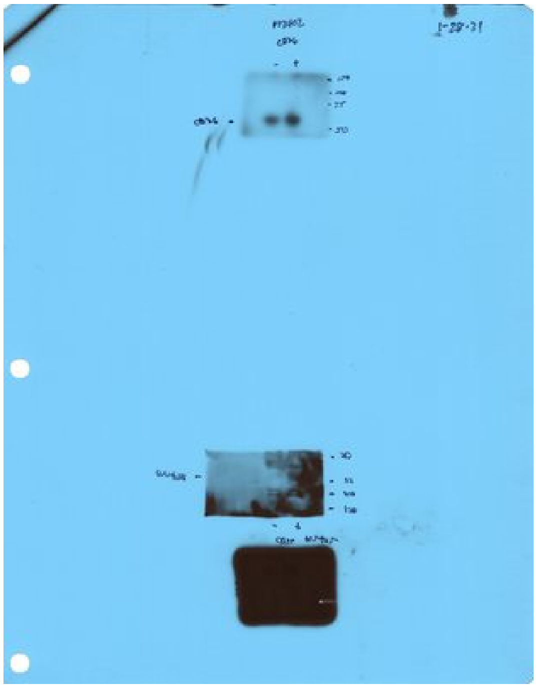

img01292021_014.jpg(462.1 KB) - download

James Drury (jmdr236@uky.edu) - Jul 12, 2021, 12:20 PM EDT

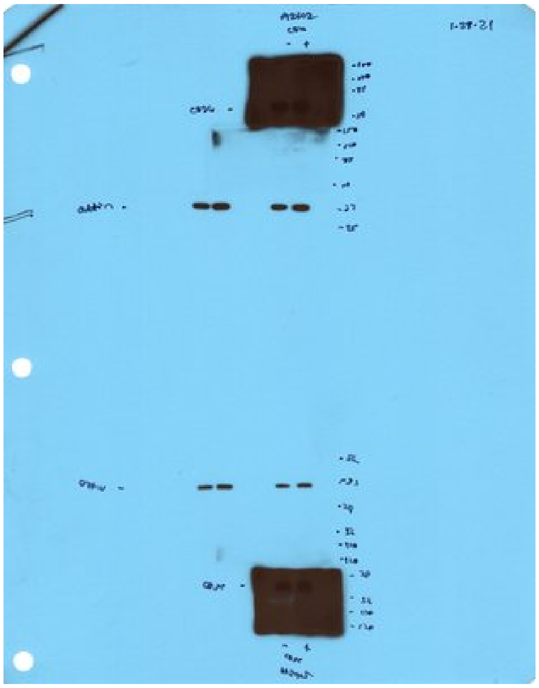

img01292021_015.jpg(604 KB) - download

James Drury (jmdr236@uky.edu) - Jul 12, 2021, 12:20 PM EDT

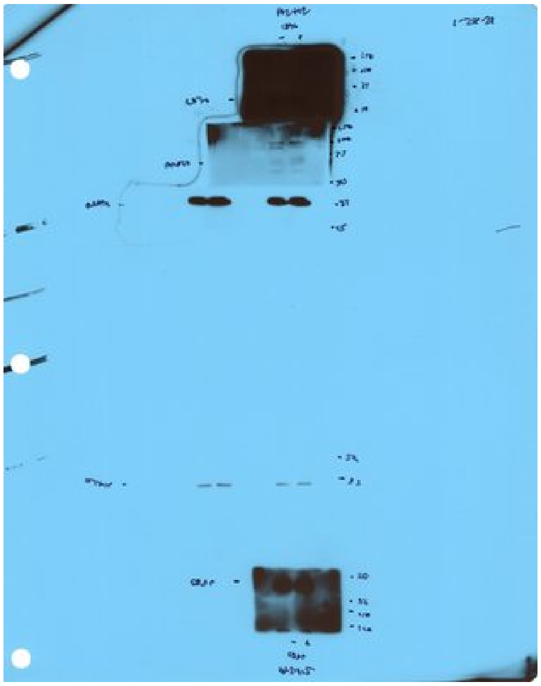

img01292021_016.jpg(467 KB) - download

James Drury (jmdr236@uky.edu) - Jul 12, 2021, 12:20 PM EDT

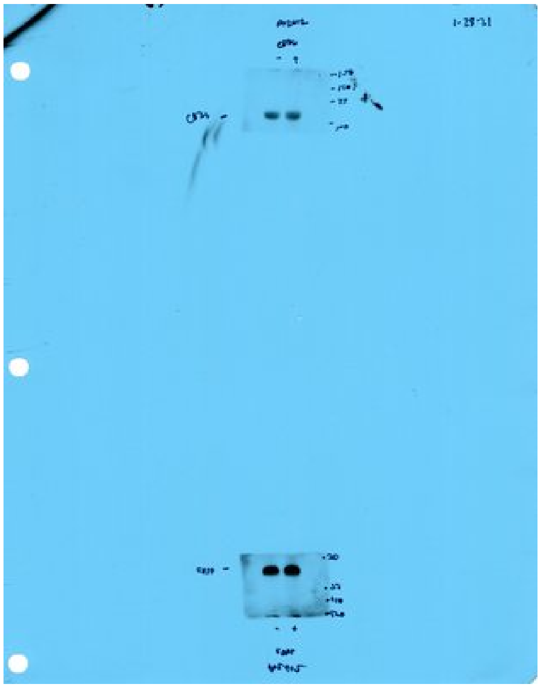

img01292021_017.jpg(631.1 KB) - download

Tissues with Pt 2377 6-29-21

James Drury (jmdr236@uky.edu) - Jul 12, 2021, 12:20 PM EDT

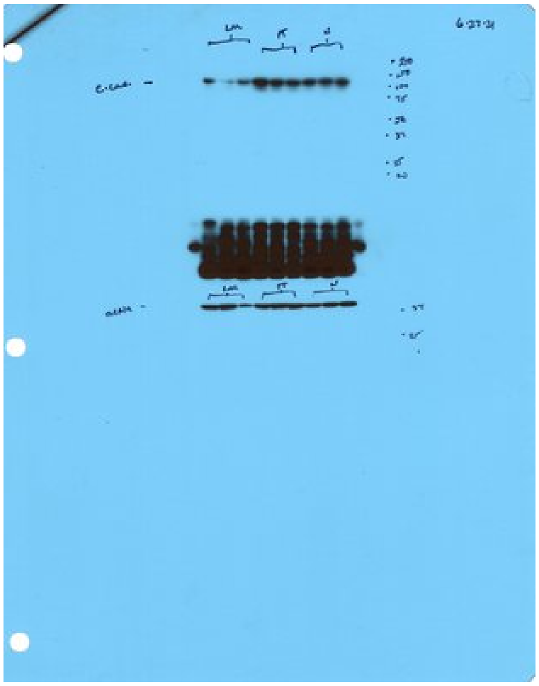

img06292021_151.jpg(524.7 KB) - download

James Drury (jmdr236@uky.edu) - Jul 12, 2021, 12:20 PM EDT

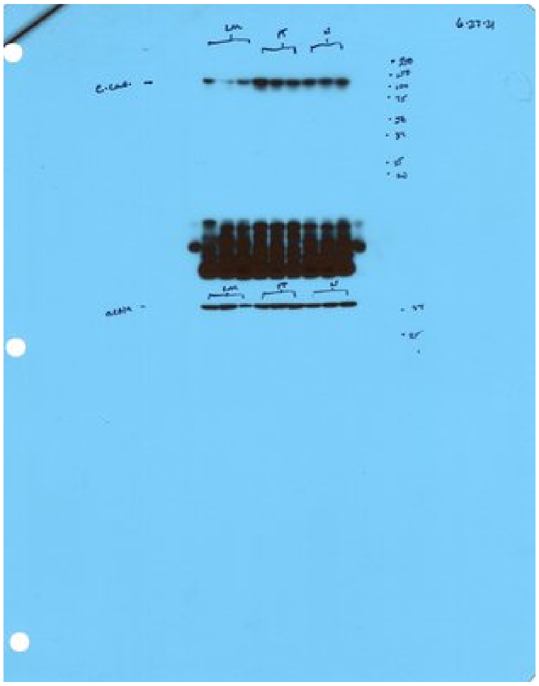

img06292021_152.jpg(524.7 KB) - download

James Drury (jmdr236@uky.edu) - Jul 12, 2021, 12:20 PM EDT

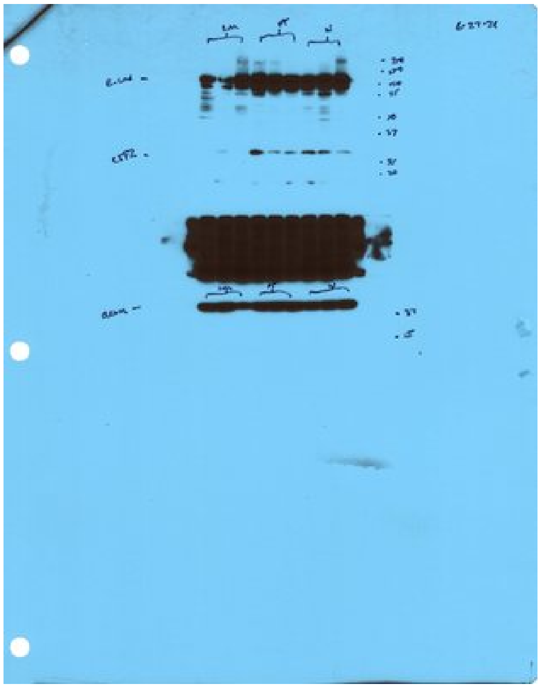

img06292021_153.jpg(535.6 KB) - download

James Drury (jmdr236@uky.edu) - Jul 12, 2021, 12:20 PM EDT

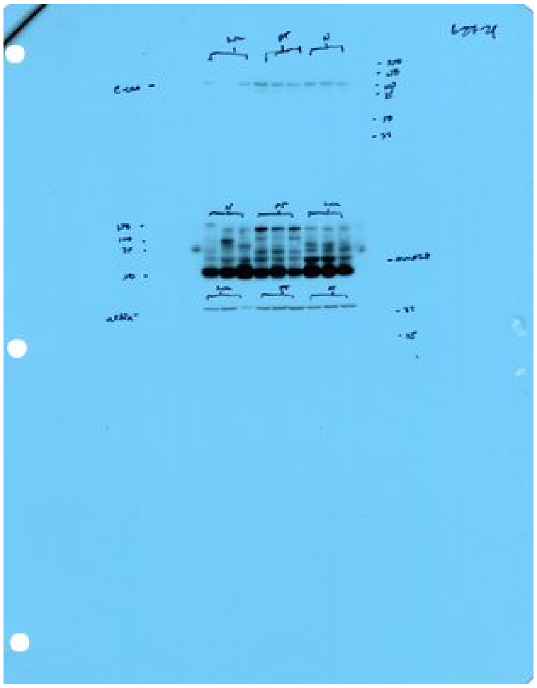

img06292021_154.jpg(554.4 KB) - download

James Drury (jmdr236@uky.edu) - Jul 12, 2021, 12:20 PM EDT

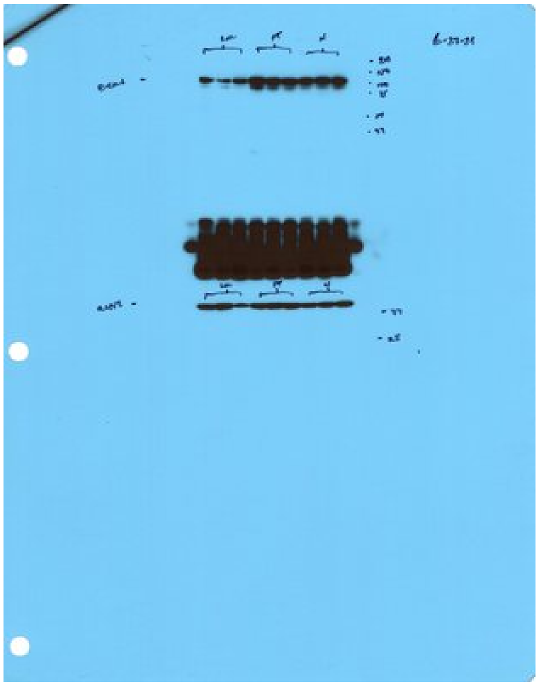

img06292021_155.jpg(524.7 KB) - download

James Drury (jmdr236@uky.edu) - Jul 12, 2021, 12:21 PM EDT

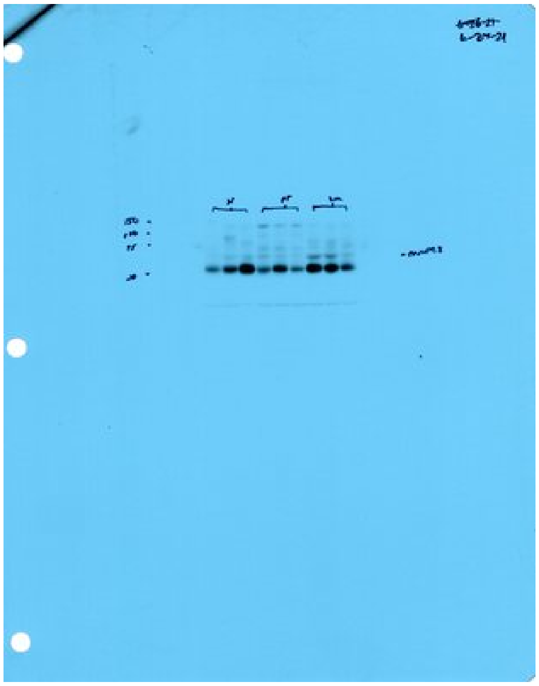

img06292021_156.jpg(524 KB) - download

James Drury (jmdr236@uky.edu) - Jul 12, 2021, 12:21 PM EDT

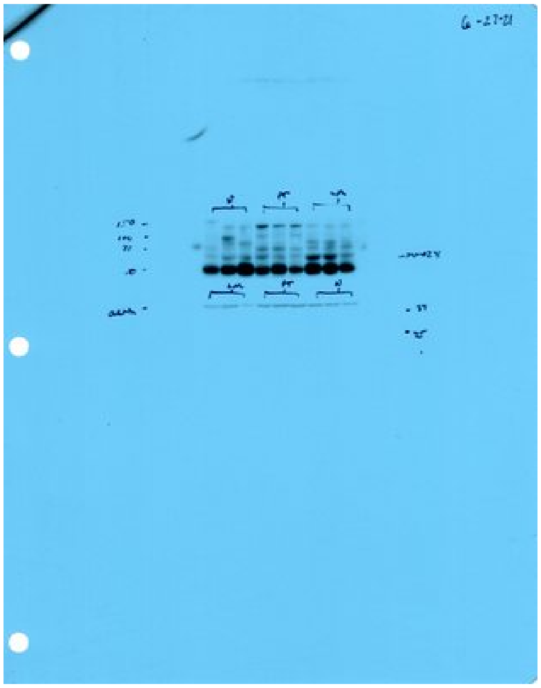

img06292021_157.jpg(532.6 KB) - download

James Drury (jmdr236@uky.edu) - Jul 12, 2021, 12:21 PM EDT

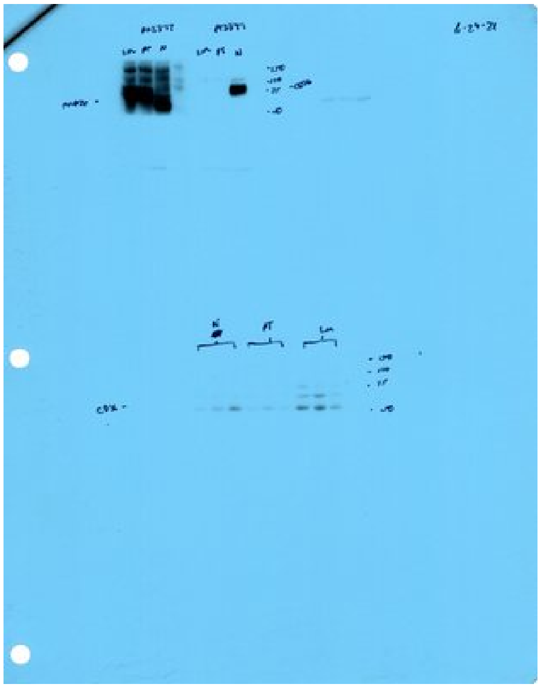

img06292021_159.jpg(527.9 KB) - download

James Drury (jmdr236@uky.edu) - Jul 12, 2021, 12:21 PM EDT

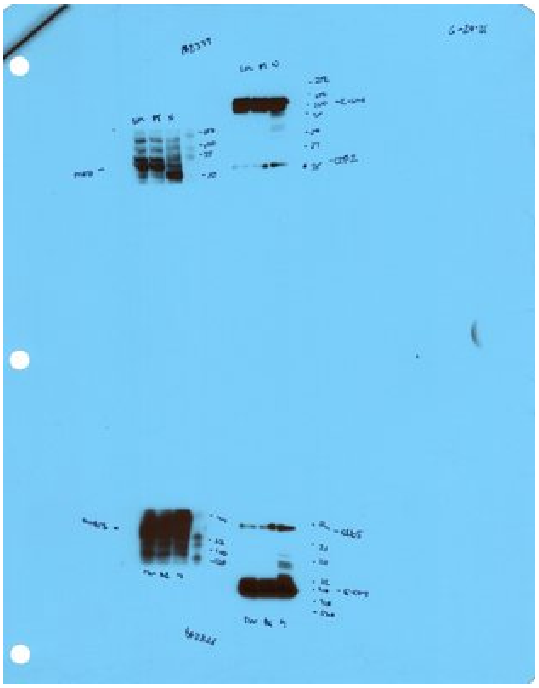

img06292021_161.jpg(536.6 KB) - download

James Drury (jmdr236@uky.edu) - Jul 12, 2021, 12:21 PM EDT

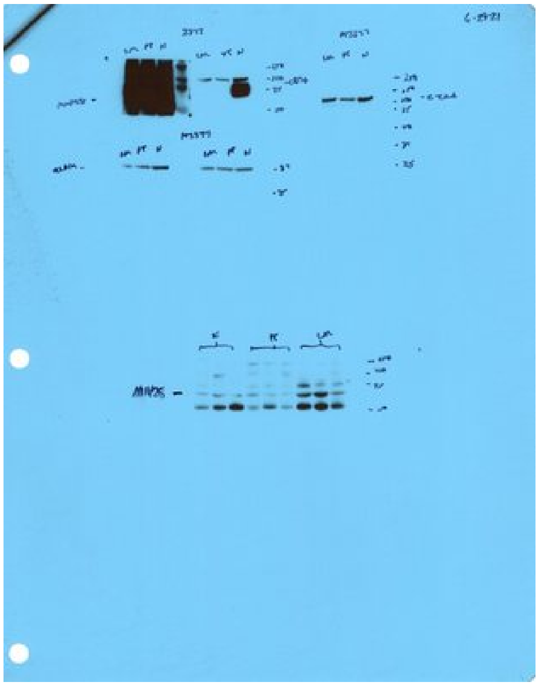

img07012021_162.jpg(515.8 KB) - download

James Drury (jmdr236@uky.edu) - Jul 12, 2021, 12:21 PM EDT

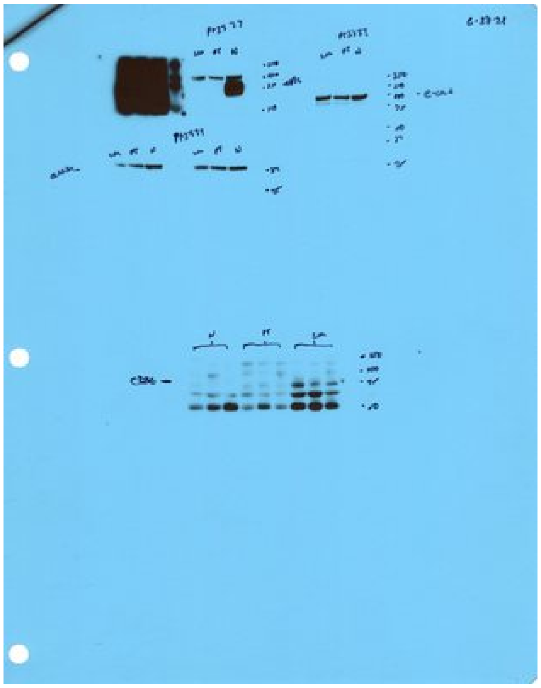

img07012021_164.jpg(662.3 KB) - download
