# Supplementary material for: Upregulation of CD36, a Fatty Acid Translocase, Promotes Colorectal Cancer Metastasis by Increasing MMP28 and Decreasing E-Cadherin Expression
Source: Cancers (Basel). 2022 Jan 5;14(1):252. doi: 10.3390/cancers14010252 (PMC8750155; doi:10.3390/cancers14010252)
Supplement: Supplementary file 1 [file cancers-14-00252-s001.zip › Figure S2.pdf]

My Notebook
Manuscripts/Revisions/Western blots

PDF Version generated by

Yekaterina Zaytseva (yyzayt2@uky.edu)

on

Dec 21, 2021 @04:16 PM EST

Table of Contents

Fig.5C 2
Fig. 5F 4
Fig. 5G 5
Supplemental_E-cadherin cleavage by MMP28 8
CD36 IgA evaluations 10
Fig. 1B 11

Fig.5C

Yekaterina Zaytseva (yyzayt2@uky.edu) - Dec 21, 2021, 3:58 PM EST

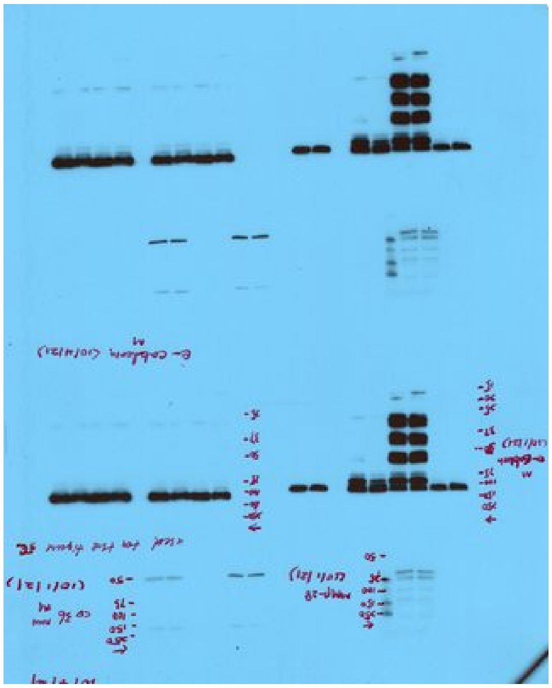

img022.jpg(752.9 KB) - download

Yekaterina Zaytseva (yyzayt2@uky.edu) - Dec 21, 2021, 3:58 PM EST

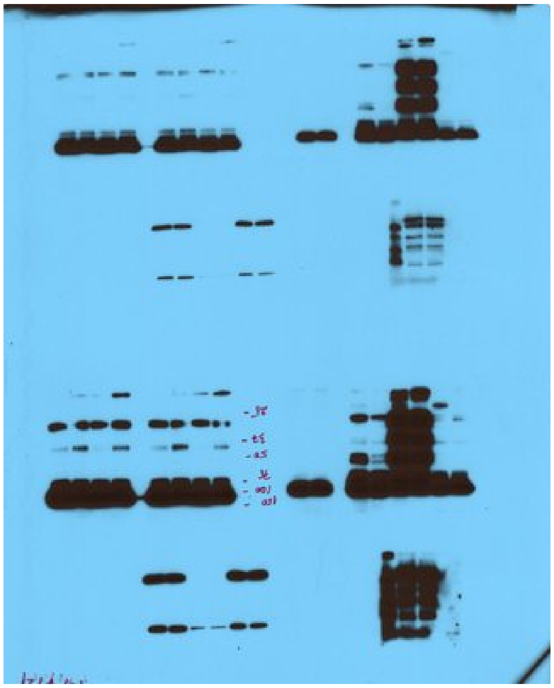

img023.jpg(562.3 KB) - download

Yekaterina Zaytseva (yyzayt2@uky.edu) - Dec 21, 2021, 3:58 PM EST

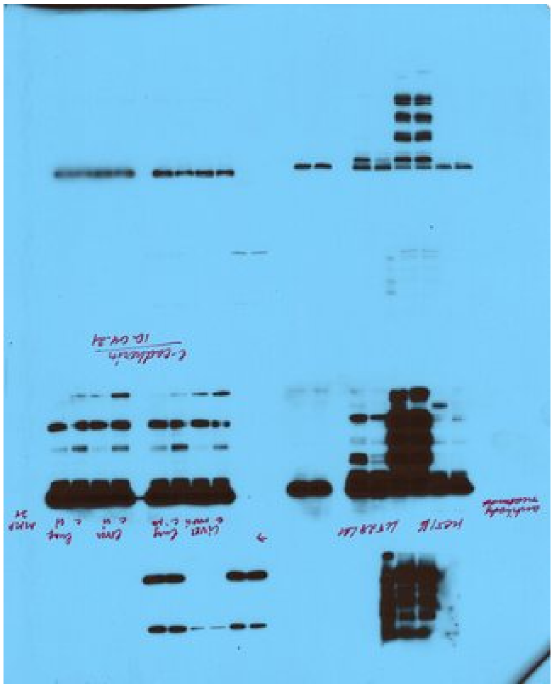

img024.jpg(729.2 KB) - download

Yekaterina Zaytseva (yyzayt2@uky.edu) - Dec 21, 2021, 3:58 PM EST

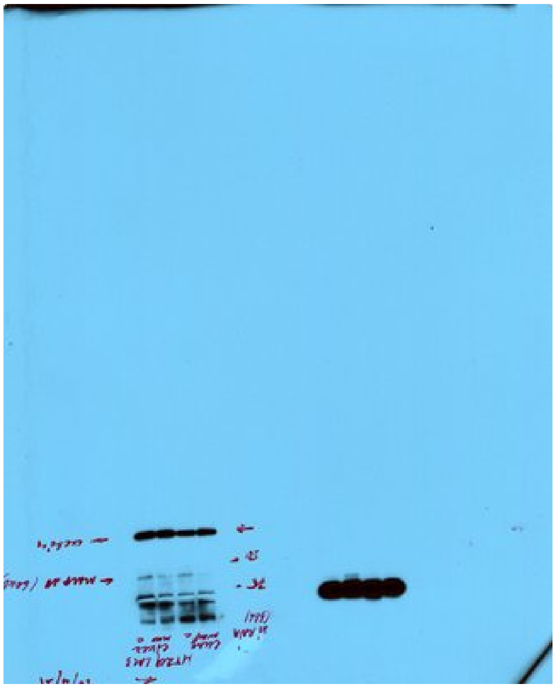

img025.jpg(557.8 KB) - download

Fig. 5F

Yekaterina Zaytseva (yyzayt2@uky.edu) - Dec 21, 2021, 3:53 PM EST

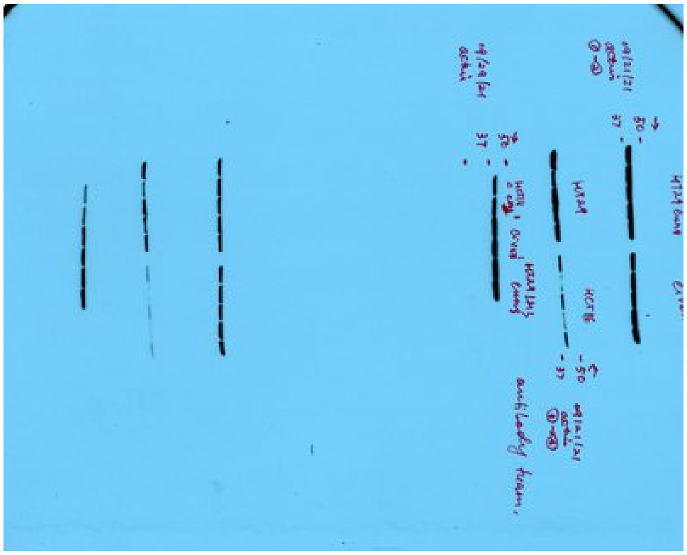

img008_1_.jpg(625.6 KB) - download

Yekaterina Zaytseva (yyzayt2@uky.edu) - Dec 21, 2021, 3:55 PM EST

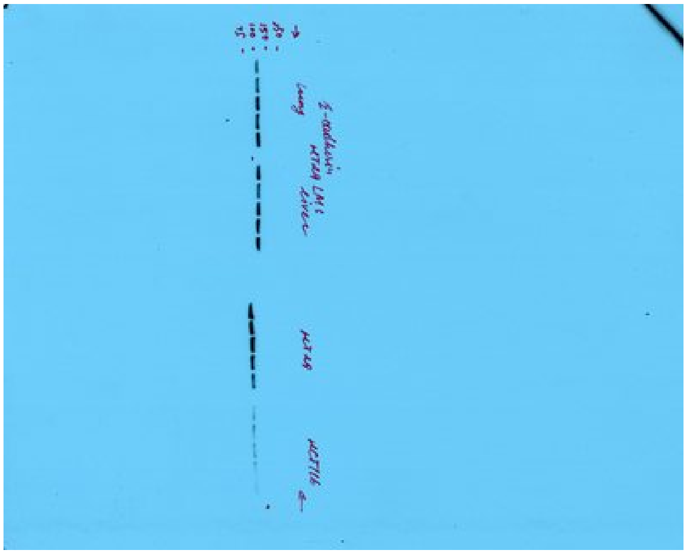

img007_1_.jpg(528.3 KB) - download

Fig. 5G

Yekaterina Zaytseva (yyzayt2@uky.edu) - Dec 21, 2021, 4:01 PM EST

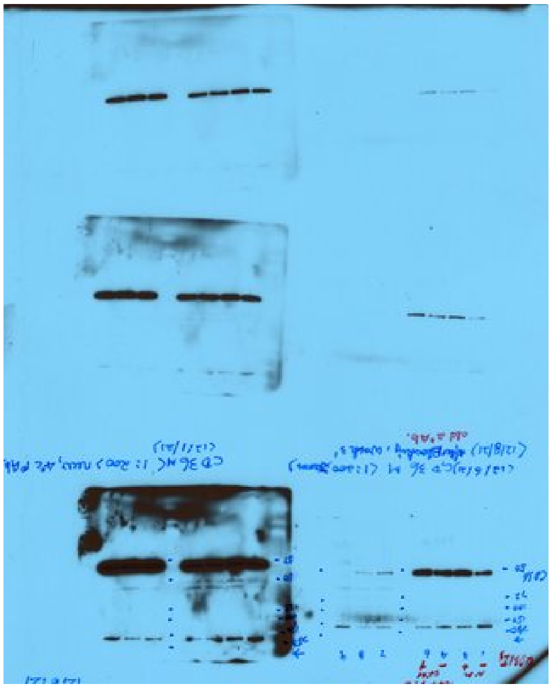

img026.jpg(548.9 KB) - download

Yekaterina Zaytseva (yyzayt2@uky.edu) - Dec 21, 2021, 4:01 PM EST

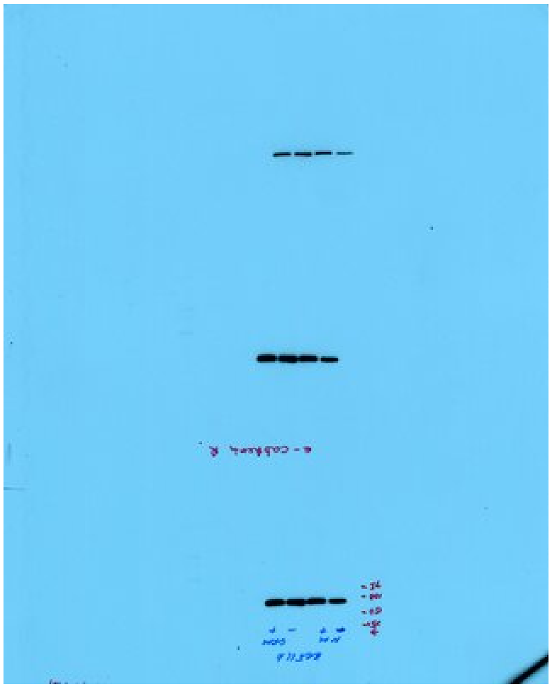

img027.jpg(501.9 KB) - download

Yekaterina Zaytseva (yyzayt2@uky.edu) - Dec 21, 2021, 4:01 PM EST

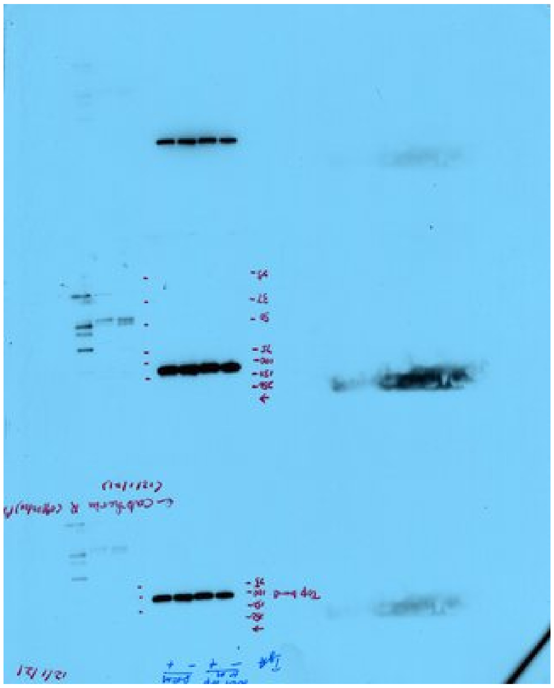

img028.jpg(653.7 KB) - download

Yekaterina Zaytseva (yyzayt2@uky.edu) - Dec 21, 2021, 4:01 PM EST

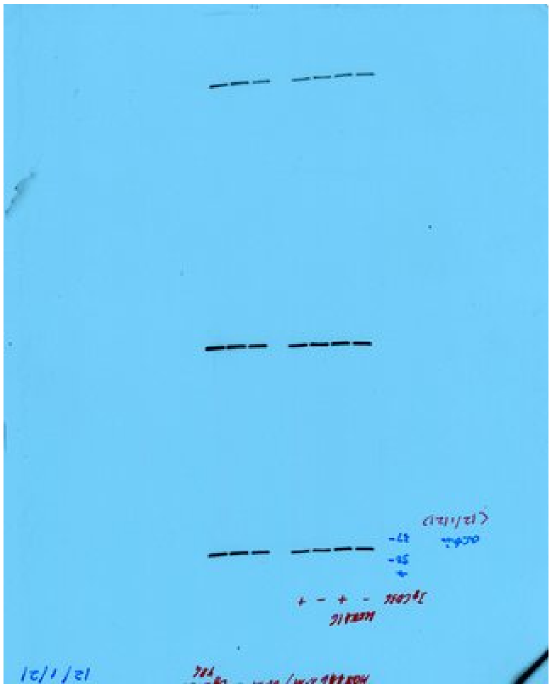

img029.jpg(658.8 KB) - download

Yekaterina Zaytseva (yyzayt2@uky.edu) - Dec 21, 2021, 4:01 PM EST

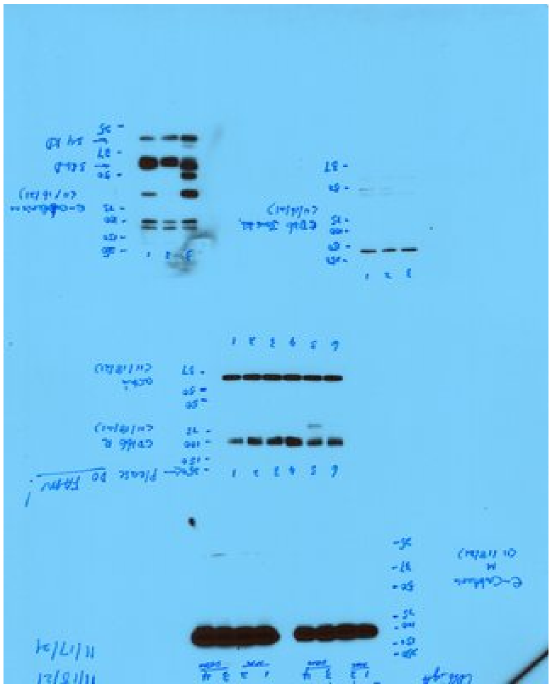

img030.jpg(491.6 KB) - download

Yekaterina Zaytseva (yyzayt2@uky.edu) - Dec 21, 2021, 4:01 PM EST

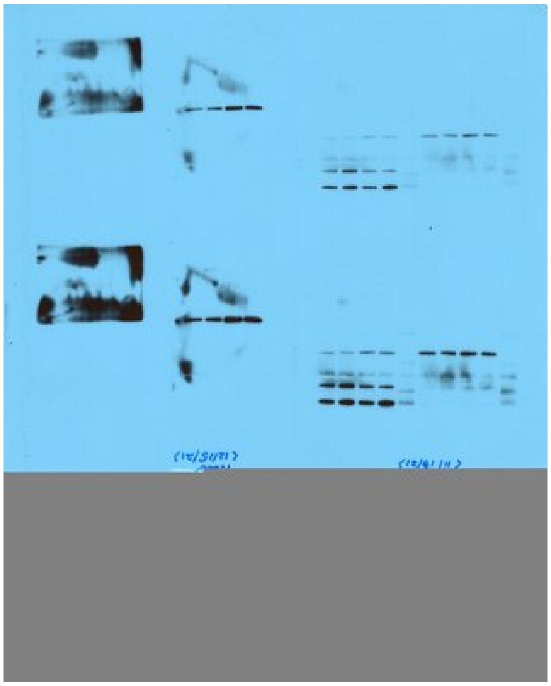

img031.jpg(530.7 KB) - download

Supplemental_E-cadherin cleavage by MMP28

Yekaterina Zaytseva (yyzayt2@uky.edu) - Dec 21, 2021, 4:02 PM EST

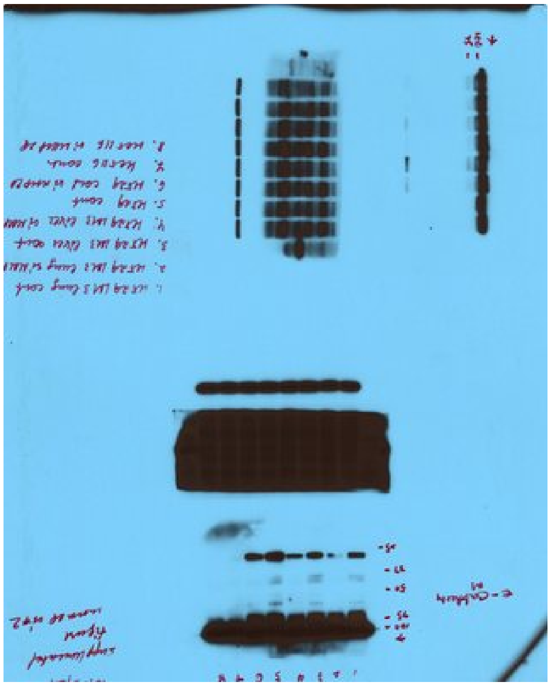

img032.jpg(627.8 KB) - download

Yekaterina Zaytseva (yyzayt2@uky.edu) - Dec 21, 2021, 4:02 PM EST

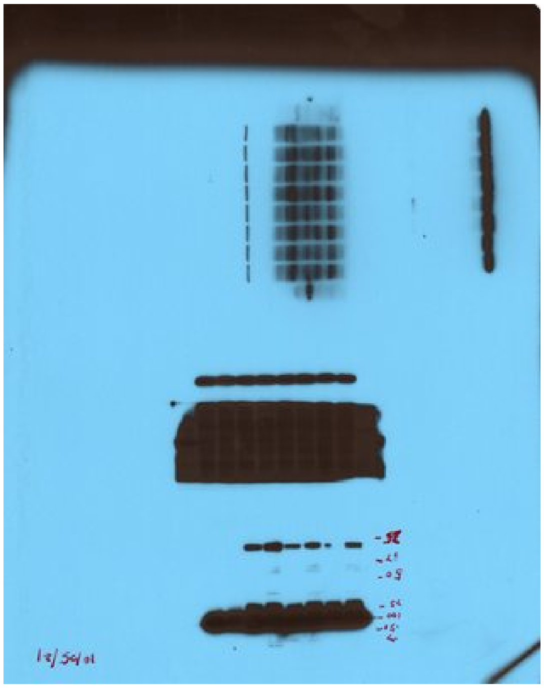

img033.jpg(584.3 KB) - download

Yekaterina Zaytseva (yyzayt2@uky.edu) - Dec 21, 2021, 4:02 PM EST

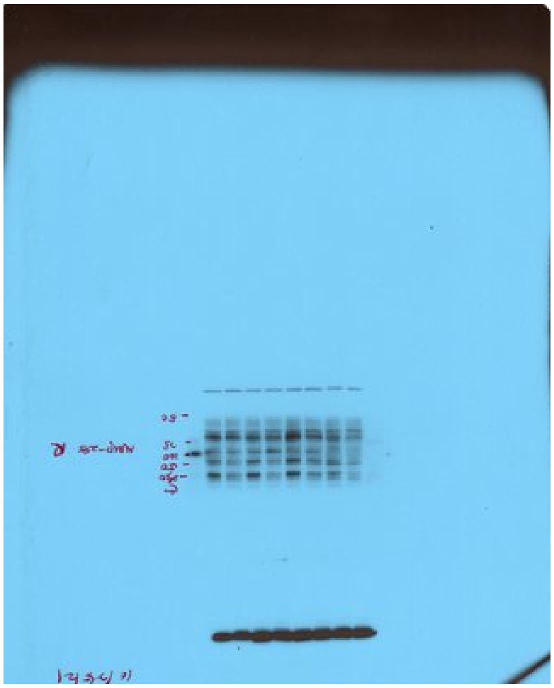

img034.jpg(565 KB) - download

Yekaterina Zaytseva (yyzayt2@uky.edu) - Dec 21, 2021, 4:02 PM EST

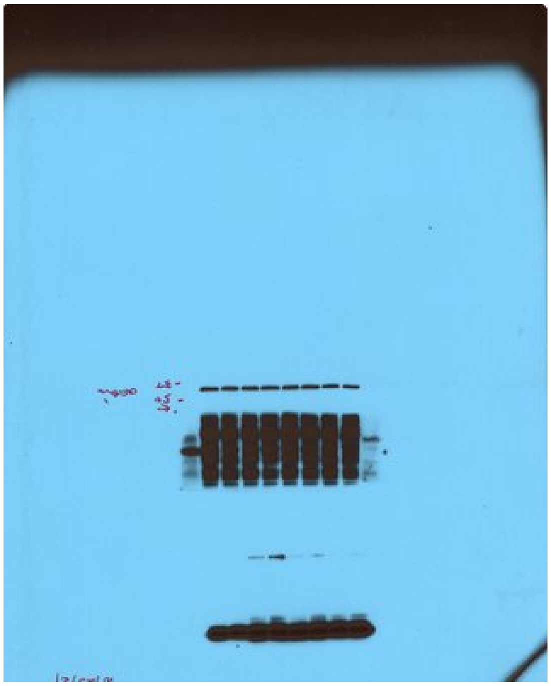

img035.jpg(557.3 KB) - download

CD36 IgA evaluations

Yekaterina Zaytseva (yyzayt2@uky.edu) - Dec 21, 2021, 4:16 PM EST

CD36_IgA_Ecadherin_quantification.xlsx(11.8 KB) - download

Fig. 1B

Yekaterina Zaytseva (yyzayt2@uky.edu) - Dec 21, 2021, 4:09 PM EST

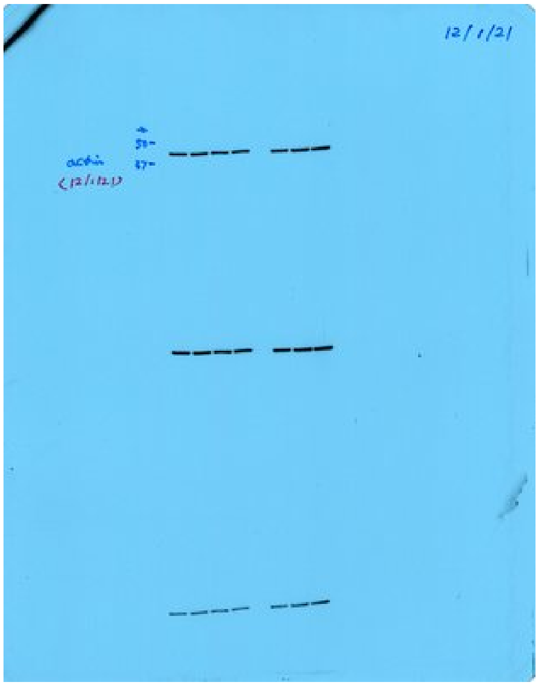

img004_1_.jpg(510.9 KB) - download

Yekaterina Zaytseva (yyzayt2@uky.edu) - Dec 21, 2021, 4:09 PM EST

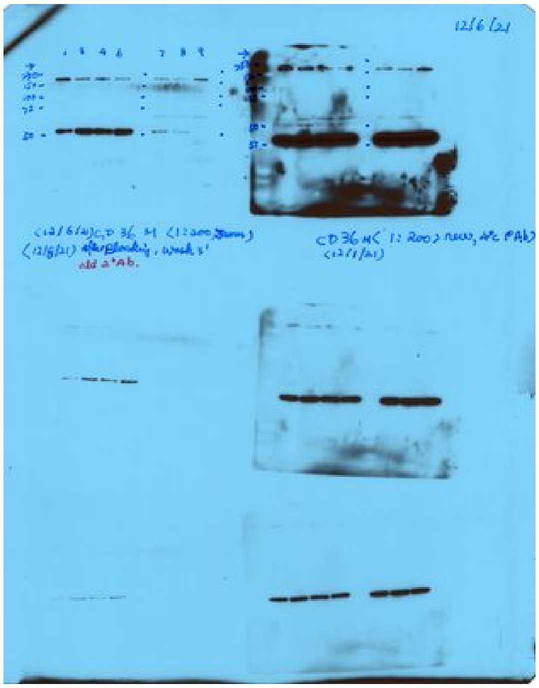

img005_1_.jpg(695.5 KB) - download

Yekaterina Zaytseva (yyzayt2@uky.edu) - Dec 21, 2021, 4:09 PM EST

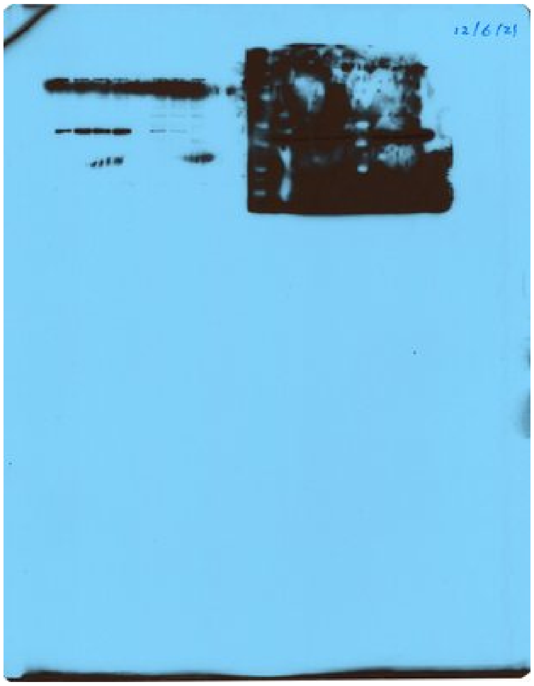

img006_1_.jpg(498 KB) - download
